# Neuroprotective Indole Diterpenoids from the Fungus *Tolypocladium album* DWS131

**DOI:** 10.3390/ph19060807

**Published:** 2026-05-22

**Authors:** Ai-Lin Liang, Chao Wang, Xing-Yi Chen, Yu-Feng Tan, Wen-Yu Lu, Peng-Ju Xu, Hong-Ping Long, Shao Liu, Jing Li, Wen-Xuan Wang, Xiaobo Xia

**Affiliations:** 1 Department of Pharmacy, National Clinical Research Center for Geriatric Disorders, Xiangya Hospital, Central South University, Changsha 410008, China; 237211030@csu.edu.cn (A.-L.L.);; 2 Xiangya School of Pharmaceutical Sciences, Central South University, Changsha 410083, China; tanyufen@csu.edu.cn (Y.-F.T.); luwenyu0417@163.com (W.-Y.L.);; 3 Eye Center of Xiangya Hospital, Central South University, Changsha 410083, China; wangchao_csu@163.com (C.W.); chen_xingyi@zju.edu.cn (X.-Y.C.); 4 Hunan Key Laboratory of Ophthalmology, Changsha 410008, China; 5 Center for Medical Research and Innovation, The First Hospital of Hunan University of Chinese Medicine, Changsha 410007, China; 310555@hnucm.edu.cn

**Keywords:** *Tolypocladium album*, *Ophiocordycipitaceae*, paxilline-type indole diterpenoids, neuroprotective activities, ferroptosis

## Abstract

**Context/Objective**: Fungi of the genus *Tolypocladium* are known for their diverse metabolic capabilities and medicinal potential. Indole diterpenoids (IDTs) represent a structurally unique class of fungal metabolites. Beyond their established roles as mycotoxins, these compounds have recently shown promise for neuroprotective effects. The objective of this study was to isolate and characterize novel IDTs from *Tolypocladium album* DWS131 and evaluate their neuroprotective activities and underlying mechanisms. **Methods**: IDTs were isolated through comprehensive chromatographic techniques. Their structures were elucidated using HRESIMS data, 1D/2D NMR spectra, and quantum chemical calculations. Neuroprotective effects were evaluated using glutamate (Glu)-induced R28 cells in vitro and N-methyl-D-aspartic acid-induced mouse models in vivo. A total of 48 mice were utilized for in vivo evaluations, divided into two separate experimental cohorts. In each cohort, mice were randomly assigned to four groups (*n* = 6 per group). Post-intravitreal injection, retinal survival and visual function were assessed via Brn3a-stained flat-mounts, H&E staining, f-VEP, f-ERG, and OptoDrum. Mechanisms involving the SLC7A11/GPX4/ACSL4 axis were investigated by Western blotting and immunofluorescence. **Results**: Seven previously undescribed paxilline-type IDTs, tolypindoles A–G (**1**–**7**), and two known analogues (**8**–**9**) were identified. Compounds **8** and **9** exhibited significant neuroprotection closely associated with the attenuation of oxidative stress and the modulation of ferroptosis-related pathways in Glu-induced R28 cells. In vivo, they preserved retinal ganglion cells, maintained retinal structure, and protected visual function, with compound **8** demonstrating superior efficacy. Mechanistic investigations revealed that both compounds modulate the SLC7A11/GPX4/ACSL4 signaling axis. **Conclusions**: This study expands the chemical diversity of *T. album* DWS131. Compounds **8** and **9**, characterized by isopentenyl moieties, highlight a promising therapeutic potential for retinal neurodegenerative diseases such as glaucoma.

## 1. Introduction

Fungi represent a vast and widely distributed biological group in nature, harboring abundant biosynthetic gene clusters for secondary metabolites in their genomes [1], thus serving as a crucial resource for the discovery of novel and bioactive natural products [2]. Among them, *Tolypocladium*, a core entomopathogenic genus of *Ophiocordycipitaceae*, has shown multifunctional agricultural and medicinal value [3]. It exhibits biocontrol capabilities against plant pathogens [4], pests [5], and nematodes [6], alongside broad ecological distribution [7,8]. Since the discovery of the famous immunosuppressive drug cyclosporine A from *Tolypocladium inflatum* [9], this genus has attracted increasing attention for its medicinal potential. Recent chemical investigations have yielded diverse metabolites such as peptaibols [8], pyridines [10], and indole diterpenoids (IDTs) [11], which display a wide range of bioactivities against major human diseases. Given the robust metabolic capacity of *Tolypocladium*, significant research interest has emerged in exploring potential health benefits from its fermentation-derived metabolites.

IDTs have emerged as a structurally distinctive and pharmaceutically significant class. These fungal metabolites feature a cyclic diterpenoid scaffold fused with an indole unit, biosynthetically derived from geranylgeranyl diphosphate (GGPP) and indole-3-glycerol phosphate [12]. With over 150 members reported to date, IDTs are generally categorized into paxilline-type and non-paxilline-type subgroups [13,14]. This family exhibits a broad spectrum of pharmacological activities, including cytotoxic effects [14], antiosteoclastogenic [15], antiadipogenic [16,17], antifungal [18], and agricultural pest-inhibitory [19]. Although historically recognized as neurotoxins [20], recent breakthroughs have revealed the significant neuroprotective [21] and anti-ferroptotic potential [22] of specific IDT scaffolds. For instance, 22,23-dehydro-shearinine A was shown to repress the 6-OHDA-induced apoptosis of PC12 cells via regulating the PI3K/Akt signaling pathway.

As part of an ongoing search for novel and bioactive metabolites from soil-derived fungi [10,23,24], our preliminary chemical investigation of *Tolypocladium album* DWS131 led to the isolation of a series of IDTs, among which paxilline demonstrated potent neuroprotective activity both in vitro and in vivo. Furthermore, the crude ethyl acetate extract of *T. album* DWS131 continued to exhibit distinct dose-dependent neuroprotective signals in a glutamate (Glu)-induced excitotoxicity model. While *T. album* lacks a traditional ethnopharmacological background, its identity as a soil-derived fungus positions it as a prolific source of structurally diverse and highly bioactive secondary metabolites [10,25,26]. Driven by these consecutive findings, a hypothesis-driven chemical investigation of *T. album* DWS131 was conducted to identify further responsible bioactive constituents.

**Figure 1:**
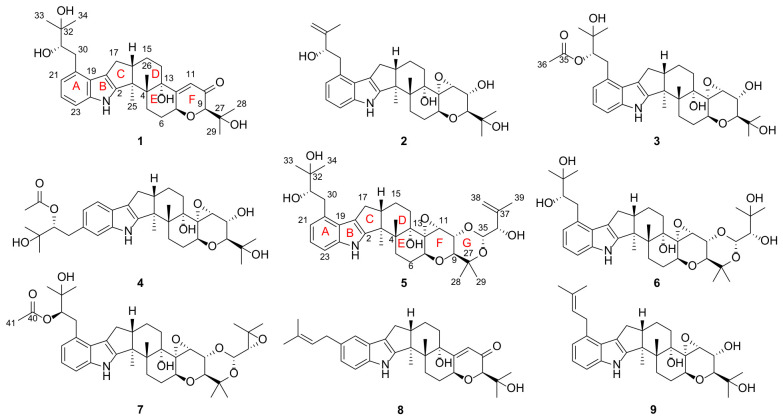
Structures of compounds **1**–**9**.

This targeted isolation led to the discovery of seven undescribed IDTs (**1**–**7**) and two known IDTs (**8** and **9**) from this fungus (Figure 1). Their structures were elucidated by 1D/2D NMR and HRESIMS data, as well as by comparison of experimental ^13^C NMR shifts with those calculated using GIAO and GFN2-NMR methods [27,28]. Biological evaluation revealed that compounds **2**, **5**, **6**, **8** and **9** displayed neuroprotective effects in a Glu-induced injury model in R28 cells. Due to the relatively weak in vitro activity of these novel metabolites (**1**–**7**), which restricted extensive biological profiling, the higher-yielding active known analogues **8** and **9** were strategically employed as representative model molecules. Notably, these two compounds were found to modulate the SLC7A11/GPX4/ACSL4 signaling axis and to inhibit ferroptosis in both in vitro and in vivo models. Herein, the details of the isolation, structural elucidation, and neuroprotective activities of these IDTs are described.

## 2. Results

### 2.1. Structural Identification

**Figure 2:**
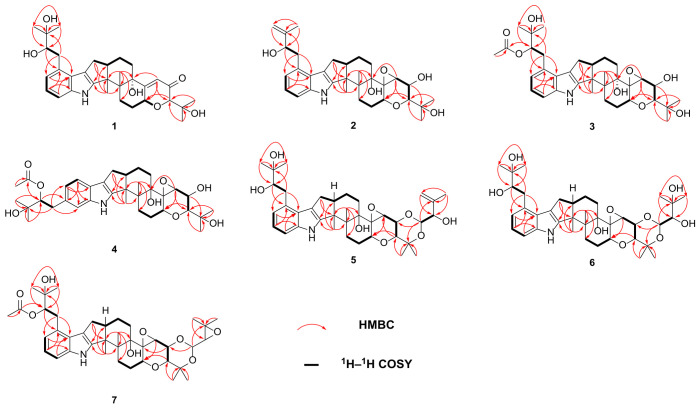
Key 2D NMR correlations of compounds **1**–**7**.

Compound **1** was obtained as a yellow amorphous powder. Its molecular formula was determined as C_32_H_43_NO_6_ on the basis of HRESIMS peak (*m*/*z* 538.3160 [M + H]^+^, calcd. for 538.3163), indicating 12 degrees of unsaturation. The UV spectrum exhibited characteristic peaks of an indole chromophore at *λ*_max_ 229 and 280 nm [29,30,31,32]. The ^1^H NMR spectrum (Table S1) of 1 displayed signals attributable to a 1,2,3-trisubstituted aromatic ring at *δ*_H_ 6.86 (1H, t, *J* = 7.9 Hz, H-21), 6.90 (1H, d, *J* = 7.9 Hz, H-22), and 7.15 (1H, t, *J* = 7.9 Hz, H-23), six methyl groups at *δ*_H_ 1.02 (3H, s, H-26), 1.27 (3H, s, H-28), 1.27 (3H, s, H-29), 1.29 (3H, s, H-33), 1.29 (3H, s, H-34), and 1.33 (3H, s, H-25), five methine protons at *δ*_H_ 2.87 (1H, m, H-16), 3.71 (1H, d, *J* = 10.3 Hz, H-31), 3.76 (1H, d, *J* = 10.3 Hz, H-9), 4.90 (1H, m, H-7), and 5.86 (1H, br s, H-11), and six methylene groups. The ^13^C NMR and DEPT data (Table S2) indicated 32 carbon signals, classified as six methyls (*δ*_C_ 16.6, 19.6, 25.3, 25.3, 26.1, and 26.3), six methylenes (*δ*_C_ 22.2, 27.9, 29.9, 30.6, 34.5, and 36.5), four sp^2^ methines (*δ*_C_ 110.8, 120.2, 121.0, and 121.1), four sp^3^ methines (*δ*_C_ 74.6, 79.9, 84.6, and 51.2), six sp^2^ nonprotonated carbons (*δ*_C_ 116.6, 126.2, 131.5, 141.9, 153.3, and 171.8), five sp^3^ nonprotonated carbons (*δ*_C_ 44.2, 51.8, 73.4, 74.1, and 77.7), and one carbonyl (*δ*_C_ 200.1). These spectroscopic features confirmed **1** as an IDT. Detailed comparison of the 1D/2D NMR data of **1** with those of paxilline, a known IDT obtained in our initial chemical investigation of this strain [33], revealed that a proton at C-20 in paxilline was replaced by a dihydroxylated isopentenyl group in **1**. Evidence for this included the absence of the C-21 methine signal and the presence of signals for five aliphatic carbons (*δ*_C_ 26.1, 26.3, 36.5, 79.9, and 74.1) in **1**. This deduction was further corroborated by the HMBC correlations from H-30 to C-19/C-21/C-32, H-33 to C-31/C-32/C-34, and H-34 to C-31/C-32/C-33 (Figure 2).

**Figure 3:**
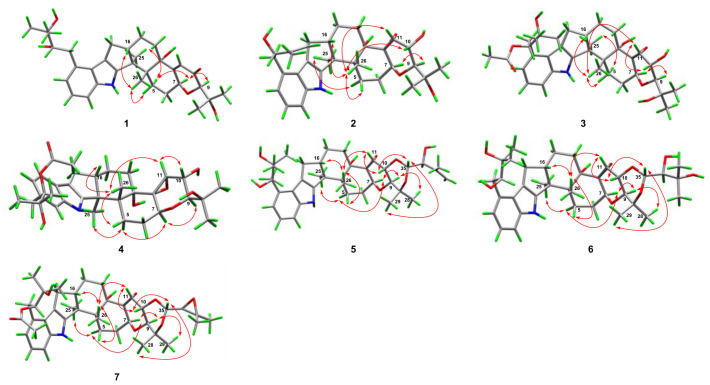
Key NOESY correlations of compounds **1**–**7**.

The relative configuration of rings A–C in **1** was determined to be identical to that of paxilline, based on analogous NOESY correlations and their shared biosynthetic origin. The NOESY correlations (Figure 3) of CH_3_-26 with H_b_-5/H-16, of H_a_-5 with H-7/CH_3_-25, and of H-7 with H-9, indicated the *α*-orientations of H-7, H-9, and CH_3_-25, and the *β*-orientations of H-16 and CH_3_-26. Consequently, the D/E ring junction was assigned as *trans* [34,35], positioning the 13-OH group opposite to CH_3_-26. The relative configuration of the cyclic framework in **1** was further confirmed by ^13^C NMR calculations, performed following previously reported methods in which the remote side chain at C-20 was simplified to a methyl group [33,36]. Comparison of calculated shifts for diastereomers **1a** and **1b** indicated that **1a** (3S*,4R*,7S*,9R*,13S*,16S*) provided a better match with the experimental data (Figure S68). This simplified computational approach was subsequently applied to assign the relative configurations of compounds **1**–**4** (Figures S68–S71). The absolute configuration of the core structure of **1** was assigned as 3*S*,4*R*,7*S*,9*R*,13*S*,16*S* based on a pronounced Cotton effect (CE) in the short-wavelength region (λ_max_ 210–250 nm) (Figure S8), attributed to π → π* transitions of the indole chromophore. This CE was consistent with those reported for known hexacyclic IDT analogues [37,38,39]. The stereochemistry at C-31 remained unresolved and was initially targeted for determination via the Mosher ester method. However, the desired esterified product was not obtained due to interference from other hydroxyl groups in the molecule. As an alternative, the GFN2NMR approach [28] was employed to achieve rapid and accurate prediction of ^13^C NMR chemical shifts. This method has been successfully applied in determining the stereochemical configuration of side chains in paxilline-type indole diterpenoids [33] or other compounds [40]. GFN2NMR-based predictions for the two possible C-31 epimers (31*R*-**1a** and 31*S*-**1a**) established the complete absolute configuration of **1** as 3*S*,4*R*,7*S*,9*R*,13*S*,16*S*,31*S*, supported by a P_mean_ value of 59.25%, and a DP4 probability of 98.92% (Table S3).

Compound **2** was isolated as a white amorphous powder. Its molecular formula was established as C_32_H_43_NO_6_ based on the HRESIMS peak (*m*/*z* 538.3168 [M + H]^+^, calcd. for 538.3163), indicating 12 degrees of unsaturation. Comparison with the NMR data of drechmerin F, an analogue from *Drechmeria* sp. [39], revealed that **2** shares the same IDT skeleton, with the exception of deshielded chemical shifts for C-32 and C-33 (*δ*_C_ 148.9 and *δ*_C_ 111.7, respectively; Table S4). This difference indicated that the dihydroxylated isopentenyl group in drechmerin F had undergone dehydration to form a *Δ*^32^ terminal double bond in **2**, which was further supported by HMBC correlations from H-33 to C-30/C-31/C-34, H-34 to C-31/C-32/C-33, and H-30 to C-19/C-21/C-32 (Figure 2). The NOESY cross peaks (Figure 3) of H_a_-5 with H-25, of CH_3_-26 with H_b_-5/H-10/H-11/H-16, and of H-7 with H-9, along with the large coupling constant *J*_9,10_ = 9.1 Hz, and the biosynthetic background of IDTs with a hydroxy group at C-13 [13,41], allowed the determination of the *α*-orientations of H-7, H-9, CH_3_-25, and 13-OH, and the *β*-orientations of H-10, H-11, H-16, and CH_3_-26. These orientations were further confirmed by GIAO ^13^C NMR calculations using STS protocol (Figure S69). The absolute configuration of the core structure in **2** was assigned as 3*S*,4*R*,7*R*,9*S*,13*S*,16*S* according to negative CE at 230 nm and positive CE at 300 nm in the ECD spectrum (Figure S17) [39,42,43]. Similar to **1**, the absolute configuration of C-31 was deduced as *S* by GFN2NMR methods with a 41.35% P_mean_ value and a 90.83% DP4 probability (Table S2). In conclusion, the absolute configuration of **2** was determined to be 3*S*,4*R*,7*R*,9*S*,13*S*,16*S*,31*S*.

Compound **3** was also obtained as a white amorphous powder. On the basis of positive HRESIMS, **3** was assigned the molecular formula of C_34_H_47_NO_8_ (*m*/*z* 598.3372 [M + H]^+^, calcd. for 598.3374), with 42 more mass units (CH_2_CO) than that of drechmerin F. Comparison of its ^1^H and ^13^C NMR data (Tables S1 and S2) with those of drechmerin F suggested that **3** shares the same indole-diterpene core scaffold. The key structural difference was the presence of an acetoxy group located at C-31 (*δ*_C_ 81.9) in **3** instead of a hydroxyl group in drechmerin F. This assignment was confirmed by HMBC correlations from H-31/CH_3_-36 to C-35 (Figure 2). Analysis of NOESY correlations (Figure 3) and GIAO ^13^C NMR calculations using the STS protocol (Figure S70) revealed that **3** possesses the same relative configurations as **2** and drechmerin F. Furthermore, their presumed shared biosynthetic origin as IDTs [13,34,37,44,45] and similar NMR data (Tables S1 and S2) collectively supported that **2**, **3**, and drechmerin F possess the same absolute configuration at the core structure. The configuration at C-31 was determined to be *S* based on GFN2NMR predictions (Table S5). Accordingly, the absolute configuration of **3** was established as 3*S*,4*R*,7*R*,9*S*,13*S*,16*S*,31*S* (Figure 1).

Compound **4** was obtained as white crystals. It presented the same molecular formula C_34_H_47_NO_8_ as that of **3** by HRESIMS peak (*m*/*z* 598.3373 [M + H]^+^, calcd. for 598.3374), suggesting they are a pair of isomers. The ^1^H NMR spectrum of **4** (Table S1) revealed aromatic proton coupling constants that differ from those of **3**, indicating a distinct substitution pattern on the benzene ring. The HMBC correlations (Figure 2) of H-30 with C-21/C-23, H-31 with C-22/C-33/C-34/C-35, CH_3_-33 with C-31/C-32, and CH_3_-36 with C-35 revealed the presence of a 3-hydroxy-3-methyl-1-butan-2-yl acetate moiety, which was attached to C-22. The relative configuration of **4** was assigned as identical to that of **3** on the basis of the NOESY data (Figure 3) and analysis of NMR data (Table S2), which was further validated by the GIAO ^13^C NMR calculation (Figure S71). Based on the negative CE in the short-wavelength region (210–250 nm) and the shared biosynthetic pathway [13,34,37,44,45], the absolute configuration of **4** at the core structure was determined as 3*S*,4*R*,7*R*,9*S*,13*S*,16*S*. The absolute configuration at position C-31 was determined to be *R* by the results of ^13^C NMR chemical shift prediction at GFN2NMR level (Table S6). Therefore, the absolute configuration of **4** was confirmed as 3*S*,4*R*,7*R*,9*S*,13*S*,16*S*,31*R*.

Compound **5** was isolated as a white amorphous powder. Its molecular formula was determined to be C_37_H_51_NO_8_ based on the HRESIMS peak (*m*/*z* 638.3689 [M + H]^+^, calcd for 638.3687), indicating 13 degrees of unsaturation. The UV spectrum exhibited absorptions at approximately 230 and 280 nm, suggesting the presence of typical indole chromophores. Investigation of the ^1^H NMR data (Table S1) and the HSQC data of **5** indicated the characteristic pattern of a 6/5/5/6/6/6/6 polycyclic IDT skeleton, including three aromatic protons, one olefinic proton, eight methines, six methylenes, and five methyl groups. In addition, analysis of the ^13^C NMR data (Table S2) showed extra resonances of twelve non-hydrogenated carbons, containing six sp^2^ carbons. The aforementioned NMR characteristics closely resembled those of the known analogue tolypocladin F [42]. The main differences were the presence of an additional hydroxy at C-36 in **5** instead of a proton in tolypocladin F, and migration of the terminal double bond from *Δ*^36^ to *Δ*^37^. These structural variations were secured by ^1^H–^1^H COSY correlation of H-35/H-36, along with the HMBC correlations from H-35 to C-37, H-38 to C-36/C-39, and H-39 to C-36/C-38 (Figure 2). The relative configuration of **5** was assigned similarly to tolypocladin F, except for the chiral centers at C-31 and C-36, based on analysis of the NOESY data (Figure 3). The NOESY correlations from H_a_-5 to CH_3_-25/H-7, H-9 to H-7/CH_3_-28, H-10 to H-11/H-35, CH_3_-26 to H_b_-5/H-11/H-16, and H-35 to CH_3_-29, indicated that H-7, H-9, and CH_3_-25 were in the *α*-orientation, while H-10, H-11, H-16, CH_3_-26, and H-35 were in the *β*-orientation. Based on the common biosynthetic pathway [13,34,37,44,45] and similar NMR data, the absolute configuration at the core structure of **5** was supposed to be consistent with that of tolypocladin F. Moreover, the absolute configurations at C-31 and C-36 were determined to be 31*S* and 36*S*, respectively, based on the DP4 probability in ^13^C NMR chemical shift calculation at GFN2NMR level (Table S7). As shown in Figure 1, the absolute configuration of **5** was determined to be 3*S*,4*R*,7*S*,9*S*,10*R*,11*R*,12*S*,13*S*,16*S*,31*S*,35*S*,36*S*.

Compound **6** was obtained as a white amorphous powder. It presented the molecular formula C_37_H_54_NO_9_, determined by analysis of HRESIMS data (*m*/*z* 656.3792 [M + H]^+^, calcd. for 656.3793), with one degree of unsaturation less than **5**. The main difference was the hydration of the *Δ*^37^ double bond in **5** to afford the 1,2-dihydroxy-2-methyl unit at C-35 in **6**, which was supported by the ^1^H–^1^H COSY correlations (Figure 2) of H-35/H-36, and the HMBC correlations (Figure 2) from H-35/CH_3_-38/CH_3_-39 to C-37, and from CH_3_-38/CH_3_-39 to C-36. The similar NOESY correlations (Figure 3) and NMR data (Tables S1 and S2) suggested that **6** and **5** shared the same relative configurations. The absolute configuration of **6** was assigned as identical to that of **5** and tolypocladin F, based on their shared structural skeleton, biogenetic origin, and closely comparable ECD curves (Figure S55).

Compound **7** was isolated as a white amorphous powder. The molecular formula of **7** was established as C_39_H_53_NO_9_ based on the pseudomolecular ion peak (*m*/*z* 680.3807 [M + H]^+^, cald. for 680.3793) in the HRESIMS spectrum, accounting for 14 degrees of unsaturation. The ^1^H and ^13^C NMR data of **7** (Tables S1 and S2) closely resembled those of terpendole N, an analogue derived from *Pleurotus ostreatus* [46], except for the additional acetyl group signals resonating at *δ*_H_ 1.76 (3H, s) and *δ*_C_ 20.9, 170.7. The HMBC correlations (Figure 2) from H-31 to C-40/C-20, CH_3_-33/CH_3_-34 to C-31, and CH_3_-41 to C-40 suggested the position of the extra acetyl group at C-31, which was in agreement with the addition of one degree of unsaturation compared with terpendole N. This difference implied that **7** was an acetylation product of terpendole N. The relative configuration of rings D–E in compound **7** was determined to be identical to that of **5**, **6**, and terpendole N, as evidenced by the NOESY cross-peaks of H_a_-5 with H-7/CH_3_-25, H-9 with H-7/CH_3_-29, H-10 with H-11/H-35, CH_3_-26 with H_b_-5/H-16/H-11, and H-35 with CH_3_-28 (Figure 3). Subsequently, the absolute configuration of **7** was established by comparison of its experimental CD spectrum with that of terpendole N, both displaying negative CE at approximately 243 and 279 nm, and positive CE near 333 nm (Figure S65). Additionally, GFN2NMR calculations also indicated that the absolute configurations at C-31 and C-36 were 31*R* and 36*S* (Table S8), respectively, validating the reliability of this computational approach.

In addition to the above compounds, two known paxilline-type IDTs, 21-isopentenylpaxilline (**8**) [31] and tolypocladin A (**9**) [42], were purified and determined by comparing their NMR and MS data with those reported.

### 2.2. Neuroprotective Effects of Compounds ***1***–***9*** Against Glu-Induced Excitotoxicity in R28 Cells

**Figure 4:**
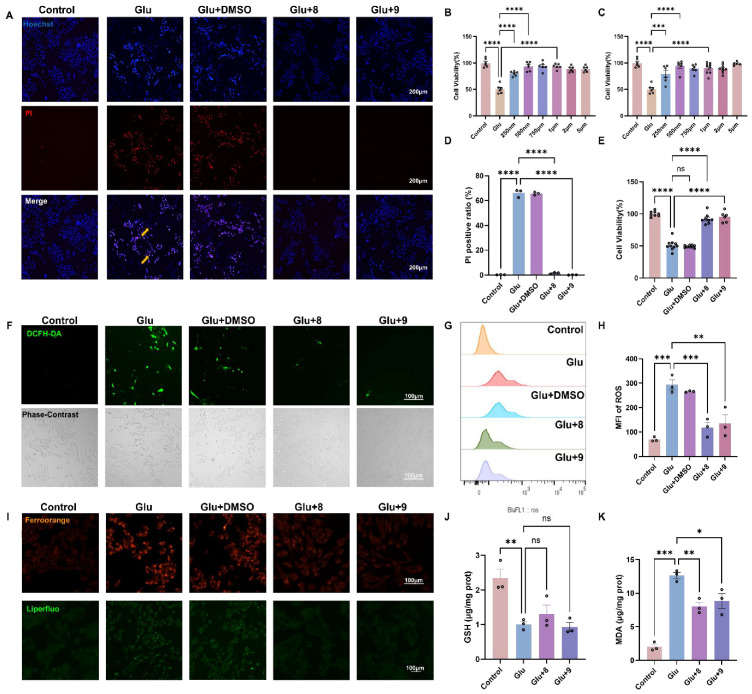
Compounds **8** and **9** protected against Glu-induced R28 cell death. (**A**) Representative images of PI (red) and Hoechst (blue) staining of R28 cells treated with Glu alone or Glu with DMSO, compound **8**, or compound **9**. Yellow arrows indicate Glu-induced excitotoxicity in R28 cells. Scale bar: 200 μm. (**B**,**C**) Effects of different concentrations of compounds **8** and **9** on R28 cell viability. (**D**) Statistical analysis of the proportion of PI-positive cells among Hoechst-stained cells. (**E**) CCK-8 assay results showing the viability of R28 cells treated with Glu and compounds **8** and **9** for 24 h (*n* = 3). (**F**) Representative images of DCFH-DA fluorescence (green) in R28 cells. Scale bar: 100 μm. (**G**–**K**) Flow cytometric analysis of intracellular levels of ROS, GSH, and MDA in R28 cells following treatment with Glu and compound **8** or **9** (*n* = 3). (**I**) Images illustrating the measurement of intracellular ferrous ion levels and lipid peroxides in R28 cells treated as indicated (Scale bar: 100 µm). FerroOrange produces an orange fluorescent signal upon reacting irreversibly with intracellular Fe^2+^ in live cells. Liperfluo selectively reacts with lipid peroxides, producing strong green fluorescence at the cell membrane upon oxidation. Data were presented as mean ± SEM; **** *p* < 0.0001, *** *p* < 0.001, ** *p* < 0.01, * *p* < 0.05, ns: not significant, compared with NMDA group.

Our previous investigation demonstrated that the crude extract of *T. album* DWS131, as well as specific monomeric constituents isolated from it, exhibited significant neuroprotective properties [33]. Guided by the distinct cytoprotective signals of the crude extract, compounds **1**–**9** were evaluated for their neuroprotective activity against Glu-induced excitotoxicity in R28 cells. Initially, cell viability was assessed by CCK-8 assay following 24 h treatment of R28 cells with 10 mM Glu alone or in combination with different concentrations of all test compounds. Compared with the Glu group, compounds **2**, **5**, **6**, **8**, and **9** significantly increased cell viability, whereas the remaining compounds showed no protective effect (Figure 4B,C and Figure S72). Although the newly identified compounds **2**, **5**, and **6** exhibited moderate neuroprotective effects, the known analogues **8** and **9** demonstrated remarkably superior efficacy, restoring viability to 95.2 ± 4.2% and 94.5 ± 5.6% (*p* < 0.001, *n* = 6), respectively (Figure 4E).

Crucially, to establish baseline safety, the intrinsic cytotoxicity of the highly active compounds **8** and **9** was evaluated on normal R28 cells. The results demonstrate that neither compound exhibited significant cytotoxicity when applied alone at concentrations up to 10 μM, with only mild toxicity appearing at 20 μM (Figure S73). This confirmed a safe therapeutic window for their application. Consistently, Hoechst/PI staining was utilized to visualize the neuroprotective effect of compounds **8** and **9**. The results demonstrate that treatment with compound **8** or **9** significantly reduced the proportion of PI-positive cells among Hoechst-positive R28 cells compared with the Glu group (Figure 4A,D), indicating a reduction in Glu-induced cell death. Collectively, these findings confirmed that compounds **8** and **9** exerted potent neuroprotective effects in an in vitro glaucoma model.

### 2.3. Compounds ***8*** and ***9*** Inhibited Ferroptosis Signaling in Glu-Induced Excitotoxicity Model of R28 Cells

Ferroptosis is driven by Fe^2+^- and reactive oxygen species (ROS)-dependent accumulation of lipid peroxides, and is morphologically, biochemically and genetically distinct from apoptosis, necrosis, and autophagy [47]. Mechanistically, this process is frequently triggered by glutathione (GSH) depletion and the subsequent inactivation of GPX4, resulting in the failure to clear lipid peroxides. This allows Fe^2+^ to drive lipid oxidation, generating ROS that execute cell death. Thus, intracellular levels of ROS, malondialdehyde (MDA), GSH, Fe^2+^ and lipid peroxides are closely associated with ferroptosis and serve as key biomarkers for its detection. Given that ferroptosis plays a critical role in retinal ganglion cell (RGC) death under Glu-induced excitotoxicity [48], ferroptosis-related markers in R28 cells treated with 10 mM Glu alone or co-treated with 500 nM compounds **8** and **9** were further examined to elucidate the neuroprotective mechanisms. As depicted in Figure 4F, DCFH-DA fluorescence staining assays revealed that Glu treatment markedly increased ROS levels compared with the control group, while compounds **8** and **9** reduced intracellular ROS accumulation. Flow cytometry analysis further confirmed these findings, showing that the mean fluorescence intensity (MFI) of ROS in the Glu + **8** and Glu + **9** groups decreased to 40.25% and 46.28% of that observed in the Glu group, respectively (Figure 4G,H). Additionally, Figure 4I–K illustrated that compounds **8** and **9** counteracted the Glu-induced effects by suppressing the elevation of MDA, Fe^2+^ and lipid peroxides. However, neither compound **8** nor compound **9** significantly restored the decreased GSH levels. Collectively, these results indicate that compounds **8** and **9** effectively alleviated Glu-induced oxidative stress and ferroptosis signaling in R28 cells.

### 2.4. Compounds ***8*** and ***9*** Protected Against N-Methyl-D-Aspartic Acid (NMDA)-Induced RGC Injury and Visual Dysfunction in Mice

**Figure 5:**
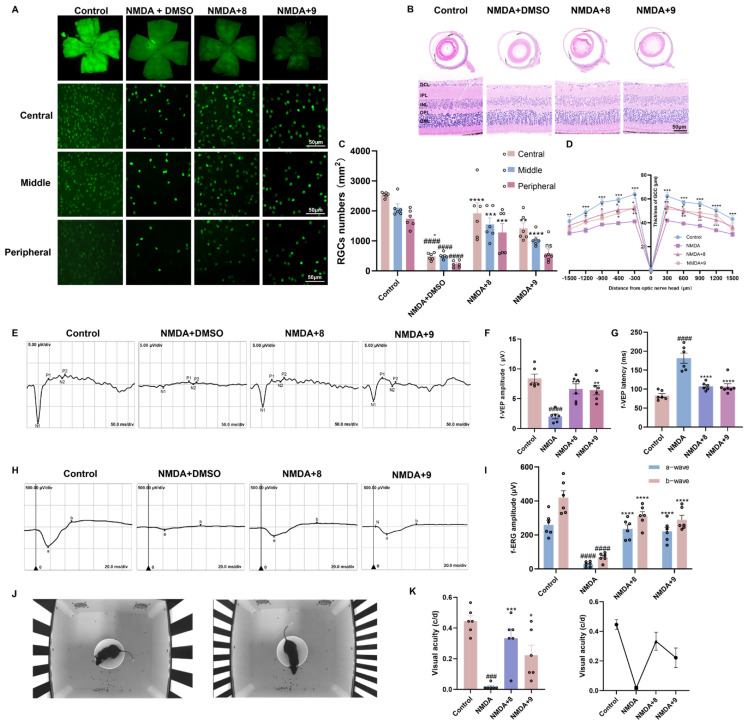
(**A**,**C**) Representative images staining with Brn3a of whole, central, middle, and peripheral regions of mouse retinal flat-mounts treated with NMDA alone or in combination with compound **8** or **9** for 5 days. green fluorescence indicates RGCs (*n* = 6, Scale bar: 50 µm). (**B**,**D**) Effects of compound **8** or **9** on retinal morphology in NMDA-induced retinopathy in mice. H&E-stained sections of mouse retina 5 days after intravitreal injection (Scale bar: 50 µm) show the following layers: GCL, INL (Inner nuclear layer), IPL (Inner plexiform layer), and ONL (Outer nuclear layer). The GCC includes the GCL and IPL. GCC thickness was measured at ±300, ±600, ±900, ±1200, and ±1600 μm from the optic nerve head compared with the NMDA group (*n* = 6). (**E**–**G**) f-VEPs were recorded 3 days post-injection of NMDA or NMDA versus compound **8** or **9** group. Changes in P1 latency and N1-P1 amplitude were recorded (*n* = 6). (**H**,**I**) f-ERGs were recorded 5 days post-injection of NMDA or NMDA versus compound **8** or **9** group. Changes in a-wave and b-wave for 3.0 cd·s/m^2^ stimulus intensities were recorded (*n* = 6). (**J**,**K**) Schematic illustration of detection of visual acuity utilizing OptoDrum used in mice treated as indicated. Visual acuity was quantified based on the optokinetic tracking response to rotating gratings of varying spatial frequencies with fixed contrast (*n* = 3). Data were presented as mean ± SEM; **** *p* < 0.0001, *** *p* < 0.001, ** *p* < 0.01, * *p* < 0.05, ns: not significant, compared with NMDA group. ^####^ *p* < 0.0001, ^###^ *p* < 0.001, compared with control group.

The neuroprotective effects of compounds **8** and **9** on RGCs were further investigated using an in vivo glaucoma model of NMDA-induced retinal injury. Retinal flat-mount immunofluorescence analysis showed that intravitreal injection of 20 μM compound **8** or **9** significantly reversed the NMDA-driven loss of RGCs in the central and middle retina (Figure 5A,C). Notably, compound **8** further safeguarded peripheral RGCs, whereas compound **9** conferred limited protection in this region (Figure 5C). To evaluate the effects of compounds **8** and **9** on NMDA-induced histological alterations of the ganglion cell complex (GCC), retinal sections were stained with Hematoxylin-eosin (H&E) (Figure 5B,D). Both compounds alleviated the NMDA-induced reduction in GCC thickness, with compound **8** demonstrating a stronger effect.

Building on the observed protection of RGCs in vivo, the potential of compounds **8** and **9** to preserve visual function and visual acuity in NMDA-injured mice was examined. The visual evoked potentials (VEPs) are a series of bioelectric potentials recorded from the surface of the occipital cortex. They are generated after a visual stimulus and assess the function of the visual pathway from the retina to the visual cortex [49]. A dysfunction at the level of the optic nerve or RGCs may alter the amplitude or latency of the VEPs. Electroretinography (ERG) is an objective measure of retinal function that records the electrical responses of retinal cells [50]. Flash visual evoked potential (f-VEP) and flash electroretinogram (f-ERG) recordings (Figure 5E–G) demonstrated that NMDA treatment prolonged P1 latency and markedly reduced N1-P1 amplitude compared with the control group. f-ERG recording revealed that the NMDA group exhibited significant reductions in both the a-wave and b-wave amplitudes at the 3.0 cd·s/m^2^ stimulus intensity compared with the control group (Figure 5H,I). Intravitreal injection of compound **8** or **9** mitigated these abnormalities. In addition, optokinetic response testing with the OptoDrum system revealed that NMDA treatment significantly reduced visual acuity, whereas treatment with compound **8** or **9** substantially reversed this decline (Figure 5J,K).

### 2.5. Modulation of the SLC7A11-GPX4/ACSL4 Ferroptosis Pathway by Compounds ***8*** and ***9*** Protected RGCs In Vitro and In Vivo

**Figure 6:**
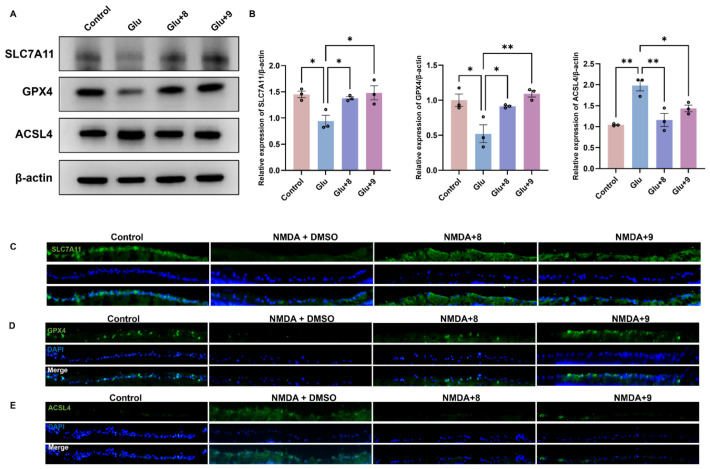
(**A**,**B**) Western blot analysis showing SLC7A11, GPX4 and ACSL4 protein expression in R28 cells treated with Glu or in combination with compound **8** or **9** for 24 h versus the control group (*n* = 3). (**C**–**E**) Fluorescence microscopy of mouse retinal sections stained for SLC7A11, GPX4 and ACSL4, 5 days after intravitreal injection of NMDA alone or in combination with compound **8** or **9**. Changes in fluorescence intensity were observed. Data were presented as mean ± SEM; ** *p* < 0.01, * *p* < 0.05.

SLC7A11 and GPX4 play crucial roles in ferroptosis by regulating the cysteine/GSH/GPX4 axis, where SLC7A11 transports cystine into cells to support GSH synthesis, and GPX4 utilizes GSH to reduce lipid peroxides, thereby preventing ferroptosis [51]. Meanwhile, ferroptosis is also tightly regulated by long-chain fatty acid metabolism via the enzyme ACSL4, which catalyzes the conversion of arachidonic acid and adrenic acid into acyl-CoA derivatives that are incorporated into phospholipids and undergo lipid peroxidation, thereby triggering ferroptosis [52]. To explore the role of GPX4 and SLC7A11 in the protection of compounds **8** and **9** on Glu-exposed R28 cells, Western blot was conducted. The results reveal that both compounds significantly restored the Glu-induced downregulation of SLC7A11 and GPX4 expression (Figure 6A,B). In addition, compounds **8** and **9** markedly suppressed the Glu-induced upregulation of ACSL4 (Figure 6A,B). To further investigate whether these compounds acted through the same ferroptosis-related pathway in vivo, retinal sections were examined by immunofluorescence. Cryosection staining (Figure 6C–E) revealed that in NMDA-treated mice, expression of GPX4 and SLC7A11 in the retinal ganglion cell layer (GCL) was markedly reduced, whereas ACSL4 expression in the GCL was significantly upregulated. Notably, intravitreal injection of compound **8** or **9** (20 μM) significantly reversed these alterations. Taken together, these results indicate that the neuroprotection conferred by compounds **8** and **9** in vitro and in vivo is closely associated with the modulation of ferroptosis in RGCs. This highlights the potential of IDTs as promising lead compounds for the development of potent neuroprotective agents.

## 3. Discussion

IDTs, with their highly complex and diverse chemical structures along with their broad and significant biological activities, have become a key focus in the field of natural product drug discovery [53]. Their biological activities are notably diverse, with cytotoxic effects constituting a predominant frontier in current research. Extensive in vitro cytotoxicity evaluations have demonstrated that these compounds can effectively inhibit the proliferation of multiple tumor cell lines. Their mechanisms of action involve key pathways such as the induction of apoptosis, cell cycle arrest, and disruption of microtubule function [11,54,55,56]. Additionally, they exhibit considerable potential in antiviral [37], antibacterial [39], anti-inflammatory [15], and *α*-glucosidase inhibitory applications [57]. It is noteworthy that although some studies have begun to explore the neuroprotective and ferroptosis inhibitory activity of IDTs, current research remains largely confined to cellular models, with systematic elucidation of their in vivo activity lacking [21,22].

In this study, all isolated compounds (**1**–**9**) were evaluated for their neuroprotective activities in an in vitro model of Glu-induced excitotoxic glaucoma. Among these, compounds **8** and **9** exhibited the highest activity, improving cell viability to 95.2 ± 4.2% and 94.5 ± 5.6%, respectively. Furthermore, compounds **2**, **5**, and **6** showed moderate activity, whereas compounds **1**, **3**, **4**, and **7** were weakly active or inactive. The preliminary structure-activity relationship (SAR) analysis revealed that the preservation of an isopentenyl moiety at C-20/C-21 is highly beneficial for maintaining potency. Specifically, the extensive oxidation of this side chain—a feature common to compounds **1**–**7**—appears to directly attenuate neuroprotective efficacy, leading to their weaker activity compared with compounds **8** and **9**. It is plausible that this modification increases hydrophilicity and may compromise critical hydrophobic interactions within the putative target binding pocket, resulting in diminished activity. Furthermore, comparisons among these analogues suggest that the introduction of a bulky acetate group on the oxidized side chain (as seen in the inactive compounds **3**, **4**, and **7**) severely blunts the neuroprotective activity, potentially due to unfavorable steric hindrance.

Further in vivo experiments revealed that intravitreal administration of compounds **8** and **9** preserved RGC numbers, maintained retinal structure, and alleviated NMDA-induced visual dysfunction, with compound **8** showing more pronounced protective efficacy. An in-depth study of their mechanism of action suggested that compounds **8** and **9** exert their neuroprotective effects by regulating the SLC7A11/GPX4 axis and ACSL4-related lipid peroxidation. While the neuroprotective mechanisms of structural analogues have been validated using inhibitors in our previous studies [33], future research incorporating rescue experiments or genetic models is still warranted to establish a stricter causal link between ferroptosis inhibition and the observed protection. This would further substantiate the precise regulatory role of IDTs and support their clinical potential.

It should be noted that although compounds **8** and **9** are known analogues, their neuroprotective activities and the underlying ferroptosis-regulatory mechanisms are reported here for the first time. Despite these promising therapeutic effects, a limitation of the current study should be acknowledged regarding comprehensive safety evaluations. Given the known neuroactive and potentially toxic nature of indole diterpenoids, systemic safety is a critical parameter. While our in vitro cytotoxicity data indicate a favorable safety profile for the active compounds at the concentrations tested, in vitro cell viability does not fully equate to systemic in vivo safety. Therefore, comprehensive in vivo toxicological evaluations, including acute toxicity and long-term tolerability assessments, are required in subsequent preclinical investigations to rigorously evaluate their safety for potential clinical translation.

## 4. Materials and Methods

### 4.1. General Experimental Procedures

Optical rotations were detected in MeOH using a Rudolph Research Analytical Autopol IV automatic polarimeter (Hackettstown, NJ, USA) operating at a wavelength of 589 nm. Infrared (IR) spectra were recorded on a Fourier Transform Infrared Spectrometer (PerkinElmer, Waltham, MA, USA) using potassium bromide pellets. Ultraviolet (UV) and experimental ECD spectra were collected on a Chirascan™-plus circular dichroism spectrometer (Applied Photophysics, Leatherhead, UK). HRESIMS data were obtained with an Agilent 6500 Q-TOF mass spectrometer (Agilent Technologies, Singapore). NMR spectra, including ^1^H, ^13^C, and 2D experiments, were measured on 600 MHz Bruker spectrometers (Bruker BioSpin, Rheinstetten, Germany) using DMSO-*d*_6_, CD_3_OD, or CDCl_3_ as solvents, with tetramethylsilane as an internal standard. The fungal extract was fractionated by column chromatography (CC) over Macroporous resin D101 system (Tianjin Haoju Resin Technology Co., Ltd., Tianjin, China), followed by 200–300 mesh silica gel (Qingdao Marine Chemical Factory, Qingdao, China) and Sephadex LH-20 (GE Healthcare, Uppsala, Sweden). Further purification was performed by preparative high-performance liquid chromatography (prep-HPLC) on either an Agilent 1260 liquid chromatography system or an *E*-Classical P3500 prep-HPLC system, both equipped with a DAD detector. The separations utilized Zorbax SB-C18 column: 9.5 × 150 mm, 5 μm (Agilent, Santa Clara, CA, USA); Supersil ODS-2 column: 10 × 250 mm, 5 μm, (Elite, Dalian, China); and Phenyl-Hexyl column: 10 × 250 mm, 5 μm (Shimadzu, Kyoto, Japan). Fractions were monitored by TLC on silica gel GF 254 plates (Qingdao Haiyang Chemical Co., Ltd., Qingdao, China), with spots visualized under UV light (254 or 365 nm) or by heating after spraying with 10% H_2_SO_4_ in ethanol. All solvents were analytical grade.

### 4.2. Fungal Material

The fungal strain *T. album* DWS131 was isolated from a humus soil sample collected from the Daweishan Forest Park in Hunan Province, China, in 2021. Strain identification was conducted by Prof. Shao Liu of Xiangya Hospital, Central South University. Its ITS sequence analysis is described in Table S9. The strain was preserved at −80 °C in glycerol stocks (20–30% *v*/*v*). A voucher specimen (No. DWS131) was deposited at Xiangya Hospital, Central South University, China.

### 4.3. Fermentation and Extraction

The strain was cultivated on potato dextrose agar medium for 8 days. The resulting culture was then fragmented into small pieces and incubated on potato dextrose liquid medium for spore production. For solid-state fermentation, a substrate of sterile water (80 mL) and rice (100 g) was prepared in Erlenmeyer flasks (500 mL) and autoclaved at 121 °C for 30 min. Each flask was inoculated with 10.0 mL of the spore suspension (50 flasks total) and incubated at 30 °C for 54 days. The fermented rice medium was fragmented and exhaustively extracted with 10 L of ethyl acetate eight times. The extract was concentrated under vacuum using a rotary evaporator (IKA, Staufen, Germany), yielding 51.0 g of dark brown residue.

### 4.4. Isolation

The isolation strategy was designed based on our prior chemical investigation of *Tolypocladium album* DWS131, which revealed that this strain produces indole diterpenoids with diverse polarities and structural features [33]. Accordingly, an orthogonal multi-stage strategy was performed combining normal-phase silica gel column chromatography, Sephadex LH-20 gel filtration, and reversed-phase preparative HPLC, with the elution conditions empirically optimized at each stage based on the polarity, molecular size, specific π–π interactions, and chromatographic behavior of the target fractions.

The extract (51.0 g) was subjected to CC on silica gel (petroleum ether/ethyl acetate/methyl alcohol, step gradient elution 50:1:0, 40:1:0, 30:1:0, 20:1:0, 10:1:0, 8:1:0, 5:1:0, 3:1:0, 2:1:0, 1:1:0, 0:0:1 to obtain nine fractions (Fr.A1–Fr.A9). Fr.A4 was separated by Sephadex LH-20 (MeOH) to give four fractions Fr.A4.1–Fr.A4.4. Fr.A4.2 was purified by preparative HPLC to yield compound **1** (4.0 mg, 40% MeCN, 3 mL/min, *t*_R_ = 40.5 min, Elite Supersil ODS-2: 10 × 250 mm, 5 μm) and **2** (4.0 mg, 66% MeOH, 3 mL/min, *t*_R_ = 41.3 min, Elite Supersil ODS-2: 10 × 250 mm, 5 μm). Fr.A4.4 was purified by preparative HPLC to yield compound **5** (2.8 mg, 70% MeOH, 3 mL/min, *t*_R_ = 23.2 min, Elite Supersil ODS-2: 10 × 250 mm, 5 μm). Separation of subfraction Fr.A5 (74.6 mg) by Sephadex LH-20 (MeOH) and preparative HPLC led to the isolation of compounds **3** (1.0 mg, 60% MeOH, 3 mL/min, *t*_R_ = 27.3 min, Elite Supersil ODS-2: 10 × 250 mm, 5 μm), **4** (1.1 mg, 67% MeOH, 3 mL/min, *t*_R_ = 40.0 min, Elite Supersil ODS-2: 10 × 250 mm, 5 μm), and **6** (2.2 mg, 44% MeCN, 3 mL/min, *t*_R_ = 21.5 min, Phenyl-Hexyl: 10 × 250 mm, 5 μm). Fr.A8 (6.5 g) was separated by Sephadex LH-20 (MeOH) to afford seven fractions (Fr.A8.1–Fr.A8.7). Fr.A8.3 and Fr.A8.5 were purified by semipreparative HPLC to give compounds **7** (3.0 mg, 33% MeCN, 3 mL/min, *t*_R_ = 32.9 min, Elite Supersil ODS-2: 10 × 250 mm, 5 μm) and **9** (5.5 mg, 65% MeCN, 3 mL/min, *t*_R_ = 26.1 min, Phenyl-Hexyl: 10 × 250 mm, 5 μm), respectively. Recrystallization of fraction Fr.A8.7 afforded compound **8** (20.0 mg) as colorless needle-shaped crystals.

*Tolypindole A* (**1**). Yellow amorphous powder; ${\left[\alpha \right]}_{D}^{25}$ −7.5 (*c* 0.04, CH_3_OH); HPLC-UV (CH_3_CN-H_2_O) *λ*_max_: 230, 281 nm; ^1^H and ^13^C NMR data, see Tables S1 and S2; (+)-HRESIMS *m*/*z* 538.3160 [M + H]^+^ (calcd for C_32_H_44_NO_6_, 538.3163).

*Tolypindole B* (**2**). White amorphous powder; ${\left[\alpha \right]}_{D}^{25}$ −58.0 (*c* 0.05, CH_3_OH); HPLC-UV (CH_3_CN-H_2_O) *λ*_max_: 230, 281 nm; IR (KBr) ν_max_: 3401, 2934, 1631, 1451, 1371, 1098, 1047, 929, 894 cm^−1^; ^1^H and ^13^C NMR data, see Tables S1 and S2; (+)-HRESIMS *m*/*z* 538.3168 [M + H]^+^ (calcd for C_32_H_44_NO_6_, 538.3163).

*Tolypindole C* (**3**). White amorphous powder; ${\left[\alpha \right]}_{D}^{25}$ −27.0 (*c* 1.0, CH_3_OH); HPLC-UV (CH_3_CN-H_2_O) *λ*_max_: 230, 281 nm; IR (KBr) ν_max_: 3404, 2980, 1725, 1453, 1376, 1250, 1116, 969, 819, 749 cm^−1^; ^1^H and ^13^C NMR data, see Tables S1 and S2; (+)-HRESIMS *m*/*z* 598.3372 [M + H]^+^ (calcd for C_34_H_48_NO_8_, 598.3374).

*Tolypindole D* (**4**). White crystal; ${\left[\alpha \right]}_{D}^{25}$ −28.9 (*c* 1.0, CH_3_OH); HPLC-UV (CH_3_CN-H_2_O) *λ*_max_: 230, 281 nm; IR (KBr) ν_max_: 3398, 2977, 1724, 1454, 1373, 1254, 1120, 930, 816, 609 cm^−1^; ^1^H and ^13^C NMR data, see Tables S1 and S2; (+)-HRESIMS *m*/*z* 598.3373 [M + H]^+^ (calcd for C_34_H_48_NO_8_, 598.3374).

*Tolypindole E* (**5**). White amorphous powder; ${\left[\alpha \right]}_{D}^{25}$ −60.6 (*c* 0.5, CH_3_OH); HPLC-UV (CH_3_CN-H_2_O) *λ*_max_: 230, 281 nm; IR (KBr) ν_max_: 3429, 2930, 1630, 1455, 1383, 1126, 928, 619 cm^−1^; ^1^H and ^13^C NMR data, see Tables S1 and S2; (+)-HRESIMS *m*/*z* 638.3689 [M + H]^+^ (calcd for C_37_H_52_NO_8_ 638.3687).

*Tolypindole F* (**6**). White amorphous powder; ${\left[\alpha \right]}_{D}^{25}$ −59.4 (*c* 0.5, CH_3_OH); HPLC-UV (CH_3_CN-H_2_O) *λ*_max_: 230, 281 nm; IR (KBr) ν_max_: 3434, 2933, 1632, 1456, 1383,1124, 1049, 925, 618 cm^−1^; ^1^H and ^13^C NMR data, see Tables S1 and S2; (+)-HRESIMS *m*/*z* 656.3792 [M + H]^+^ (calcd for C_37_H_54_NO_9_ 656.3793).

*Tolypindole G* (**7**). White amorphous powder; ${\left[\alpha \right]}_{D}^{25}$ −90.0 (*c* 1.0, CH_3_OH); HPLC-UV (CH_3_CN-H_2_O) *λ*_max_: 232, 283 nm; IR (KBr) ν_max_: 3453, 2980, 1718, 1640, 1455, 1397, 1242, 1126, 1051, 915, 604 cm^−1^; ^1^H and ^13^C NMR data, see Tables S1 and S2; (+)-HRESIMS *m*/*z* 680.3807 [M + H]^+^ (calcd for C_39_H_54_NO_9_, 680.3793).

### 4.5. Quantum Chemical Calculation Methods

The GIAO ^13^C NMR calculations for the compounds were conducted in accordance with previously described methods [27,28]. The GFN2NMR prediction of ^13^C NMR chemical shifts was performed using GFN2NMR version 1.4.2. [28]. To ensure the complete sampling of molecular conformations, the conformers of each structure for GFN2NMR prediction were obtained by combining the low-energy conformers generated by CREST version 3.0 at the GFN0-xTB level and RDKit 2025-03-2 (Q1 2025) release [58]. The energy window of GFN2NMR was set to 3 kcal/mol.

### 4.6. Cell Culture

All in vitro experiments were performed using R28 cells, a rat retinal progenitor cell line widely applied in studies of oxidative stress and cell death in RGCs [59]. The R28 cell line (Cat# CVCL_5I35) was provided by the Department of Anatomy and Neurobiology at Central South University (Changsha, China). Cells were cultured in low-glucose Dulbecco’s Modified Eagle Medium (DMEM; Gibco, Grand Island, NY, USA) supplemented with 10% fetal bovine serum (FBS; Gibco, USA) and 1% penicillin–streptomycin (Gibco, USA) at 37 °C in a humidified atmosphere containing 5% CO_2_. To establish the Glu-induced excitotoxicity model, cells were first cultured under standard conditions for 24 h, followed by treatment with Glu (ab120049; Abcam, Cambridge, UK) for an additional 24 h.

### 4.7. Cell Viability Assay and Hoechst 33342/PI Staining

R28 cells were seeded at 8 × 10^4^ cells/well in 96-well plates and incubated overnight. Then, cells were allocated into different treatment groups: the control group (cells were maintained in complete culture medium), the Glu group (cells were treated with 10 mM Glu), and the tested compounds groups (cells were co-treated with 10 mM Glu and each of the compounds **1**–**9** at 1 μM in complete culture medium). Following another 24 h incubation, the culture medium was discarded, and the cells were washed twice with PBS. CCK-8 solution (10% per well) was added for 3 h, and absorbance was measured at 450 nm. For PI/Hoechst staining, cells were seeded in 12-well plates and treated similarly. Staining was performed according to the manufacturer’s instructions (Hoechst 33342/PI kit, Beyotime, Shanghai, China) by incubating cells with staining buffer mixed with Hoechst 33342 and PI (200:1) at 4 °C for 30 min in the dark. Cells were then washed with PBS and imaged by fluorescence microscopy.

### 4.8. ROS Detection

Intracellular ROS levels were measured using a ROS Assay Kit (Beyotime, China). Cells were digested with trypsin and incubated with 10 μM DCFH-DA at 37 °C for 30 min in the dark. After PBS washes, samples were analyzed by flow cytometry (excitation 488 nm, emission 535 nm), and MFI was quantified with FlowJo 10.8.1. For fluorescence imaging, R28 cells were incubated with 10 μM DCFH-DA under the same conditions and observed by fluorescence microscopy (Leica, Wetzlar, Germany).

### 4.9. Detection of GSH and MDA

Cells were collected by centrifugation, and intracellular GSH levels were measured using a GSH assay kit (Solarbio, Beijing, China) according to the manufacturer’s instructions. Cells were lysed with GSH extraction buffer through two freeze-thaw cycles (liquid nitrogen and 37 °C water bath). The lysates were centrifuged at 8000× *g* for 10 min at 4 °C, and the supernatants were collected for GSH measurement. Cells were collected and the cell numbers or protein concentration were determined. Subsequently, MDA working solution was added at the specified ratio according to the manufacturer’s instructions (Boxbio, Beijing, China). The samples were incubated in a boiling water bath for 60 min, then cooled to room temperature in an ice bath. Subsequently, they were centrifuged at 10,000× *g* for 10 min at room temperature, and the supernatants were collected. Absorbance was measured at 450 nm, 532 nm, and 600 nm, and intracellular MDA levels were calculated accordingly.

### 4.10. Detection of Intracellular Fe^2+^ and Lipid Peroxidation

Intracellular Fe^2+^ levels were detected using FerroOrange (Dojindo, Kumamoto, Japan), and lipid peroxidation was assessed using Liperfluo (Dojindo, Kumamoto, Japan) according to the manufacturer’s instructions. Briefly, cells were incubated with FerroOrange (1 μM) or Liperfluo (2 μM) in serum-free medium at 37 °C for 30 min in the dark. After washing three times with PBS, cells were either directly imaged under a confocal fluorescence microscope or fixed with 4% paraformaldehyde for 10 min for subsequent immunofluorescence staining. Fluorescence signals were captured using excitation/emission wavelengths of 561/580 nm for FerroOrange and 488/520 nm for Liperfluo. Mean fluorescence intensity was quantified using ImageJ software version 1.54k (National Institute of Health, Bethesda, MD, USA).

### 4.11. Establishment of the NMDA Mouse Model

All animal procedures were conducted in accordance with protocols approved by the Institutional Animal Care and Use Committee (IACUC) of Central South University (Approval No. CSU-2023-0297). Animal experiments were conducted from 1 July 1 2023, to 1 December 2025. Eight-week-old C57BL/6J mice (SJA Laboratory Animal Co., Ltd., Changsha, China) were housed under a 12 h light/dark cycle with free access to food and water. The NMDA-induced excitotoxicity model was selected as it is a well-established pharmacological model that effectively mimics the core pathological process of glutamate excitotoxicity-mediated RGC degeneration, which is critically involved in conditions such as glaucoma [60].

Mice were anesthetized with sodium pentobarbital (50 mg/kg, intraperitoneally). Adequate anesthesia depth was confirmed by the absence of reflex responses before surgical manipulation. At the end of experiments, animals were euthanized by overdose of sodium pentobarbital (≥150 mg/kg, intraperitoneally). For downstream tissue collection and histological evaluation, the deeply anesthetized mice were transcardially perfused with phosphate-buffered saline. For animals not requiring perfusion, euthanasia was confirmed by cervical dislocation. Procedures followed American Veterinary Medical Association guidelines. To minimize animal suffering, humane endpoints were established and monitored daily throughout the study. In the present study, no mice reached the humane endpoints or showed signs of clinical distress prior to the scheduled experimental time points.

A total of 48 mice were divided into two independent experimental cohorts. In each cohort, mice were randomly divided into four groups (*n* = 6 per group): control (needle puncture only), NMDA (20 mM NMDA), NMDA + **8** (20 mM NMDA + 20 μM compound **8**), and NMDA + **9** (20 mM NMDA + 20 μM compound **9**). The first cohort of 24 mice was dedicated to retinal flat-mount analysis. The remaining cohort of 24 mice was utilized for visual function assessments, followed by H&E staining and mechanistic immunofluorescence analyses. In adherence to the 3R (Replace, Reduce, Refine) principles, the minimum number of mice required to achieve statistical significance was used as the sample size.

For the intravitreal injection procedure, mice were anesthetized by intraperitoneal administration of 1% sodium pentobarbital (50 mg/kg), and mydriasis was induced with tropicamide. Proparacaine and levofloxacin eye drops were applied to provide surface anesthesia and reduce infection risk. Under a stereomicroscope, a 32G needle was inserted at the corneal limbus to establish an injection channel, and 1 μL of the designated solution was injected into the vitreous cavity with a microsyringe. Based on the estimated physiological fluid volume of the mouse eye (approximately 5 μL) [61], this 1 μL injection of a 20 μM compound solution yields a final intraocular drug concentration of approximately 4 μM. This administered amount was rationally selected based on our in vitro findings, ensuring optimal neuroprotective efficacy without obvious toxicity. The needle was left in place for 30 s before withdrawal. Tobramycin–dexamethasone ointment was applied to the operated eye. Experiments were performed 5 days post-injection.

### 4.12. Retinal Flat-Mount

Five days after injection, mice were anesthetized and perfused with pre-cooled saline. Eyeballs were enucleated and post-fixed in 4% PFA for 1 h. Retinas were dissected, blocked in PBS with 5% BSA and 0.5% Triton X-100 for 2 h, and incubated overnight at 4 °C with rabbit anti-Brn3a (1:100, Abcam, Cambridge, UK). After washing with PBS, retinas were incubated with Alexa Fluor 488-conjugated goat anti-rabbit IgG (1:1000, Abcam, Cambridge, UK) for 1 h. The retinal flat-mount was imaged by fluorescence microscopy. Brn3a-positive RGCs were quantified in central, middle, and peripheral regions using ImageJ.

### 4.13. Immunofluorescence of Retinal Cryosections

Following fixation, eyeballs were cryoprotected in 30% sucrose solution overnight, embedded in OCT compound, and sectioned at 10 μm thickness. Cryosections were blocked in PBS with 5% BSA and 0.5% Triton X-100 for 2 h. Sections were incubated overnight at 4 °C with anti-GPX4, anti-SLC7A11, and anti-ACSL4 antibodies, followed by Alexa Fluor 488-conjugated goat anti-rabbit IgG (1:200, Abcam, Cambridge, UK) for 1 h. Nuclei were counterstained with DAPI (Servicebio, Wuhan, China) for 7 min. Images were acquired by fluorescence microscopy (Leica, Wetzlar, Germany) with identical exposure settings.

### 4.14. f-VEP and f-ERG

For f-VEP, mice were dark-adapted for 15 min after anesthesia. Electrodes were positioned as follows: cathode at bregma, anode at occiput, and ground electrode subcutaneously in the back, with the contralateral eye covered. Recordings were obtained using an f-VEP system with a Ganzfeld stimulator (Roland Consult, Brandenburg an der Havel, Germany), and P1 latency and N1-P1 amplitude were analyzed. For f-ERG, mice were dark-adapted overnight. After anesthesia and tropicamide-induced mydriasis, animals were maintained on a heating pad. A gold ring electrode was placed on the cornea, with the ground electrode at the tail base and a reference electrode at the canthus. Responses were recorded with the Reti-scan system (Roland Consult, Brandenburg an der Havel, Germany) under flash stimuli of 0.01, 3.0, and 10.0 cd·s/m^2^, with the 3.0 cd·s/m^2^ response analyzed as the main parameter. All recordings were performed under dim red light, and a- and b-wave amplitudes were quantified.

### 4.15. Automated Visual Acuity Testing (OptoDrum)

Visual acuity was assessed using the OptoDrum (Striatech, Tübingen, Germany) system by recording the optokinetic reflex. Mice were acclimated one day prior to testing. During testing, each mouse was placed on a central platform surrounded by four monitors displaying vertical black-and-white stripes at 12°/s and 100% contrast. Tracking responses were recorded when the eyes followed the stripes. The system automatically adjusted parameters and determined the visual threshold as the highest spatial frequency (cycles per degree, cpd) eliciting tracking. Visual acuity was measured for both eyes and compared across groups.

### 4.16. H&E Staining

Five days after injection, mice were euthanized and eyeballs were fixed in ocular fixative for 24 h, dehydrated, cleared, and paraffin-embedded. Sagittal sections (3 μm) were prepared and stained with H&E. GCC thickness was measured at ±300, 600, 900, 1200, and 1600 μm from the optic nerve head using CaseViewer (3DHISTECH, Sysmex, Budapest, Hungary).

### 4.17. Western Blotting

R28 cells were seeded at 1 × 10^6^ per 60 mm dish and divided into four groups: control, Glu (10 mM), Glu + **8** (10 mM Glu + 500 nM compound **8**), and Glu + **9** (10 mM Glu + 500 nM compound **9**). After 24 h treatment, proteins were extracted with RIPA buffer (Beyotime, Shanghai, China), quantified by BCA assay, separated on 4–20% SDS-PAGE gels, and transferred to PVDF membranes (Millipore, Burlington, MA, USA). Membranes were blocked with 5% nonfat milk in TBST for 90 min and incubated overnight at 4 °C with primary antibodies against β-actin (1:10,000, Huabio, Hangzhou, China, ET1702-67), GPX4 (1:1000, Proteintech, Rosemont, IL, USA, 67763-1-Ig), SLC7A11 (1:1000, Thermo Fisher Scientific, Waltham, MA, USA, PA1-16893), and ACSL4 (1:1000, Abcam, Cambridge, UK, ab155282). After washing, membranes were incubated with HRP-conjugated goat anti-rabbit IgG (1:10,000, Proteintech, Rosemont, IL, USA) for 1 h, and signals were detected by enhanced chemiluminescence (NCM Biotech, Suzhou, China). Intensities were quantified with ImageJ, and expression levels were normalized to β-actin.

### 4.18. Statistical Analysis

All data are expressed as mean ± SEM. Statistical analyses were performed with GraphPad Prism 9.0.0. One-way or two-way ANOVA was applied as appropriate, and all tests were two-tailed. Differences were considered statistically significant at *p* < 0.05. Significance levels were indicated as * (or #) *p* < 0.05, ** (##) *p* < 0.01, *** (###) *p* < 0.001, and **** (####) *p* < 0.0001. The # symbol indicates significant differences compared with the control group; the * symbol indicates significant differences compared with the Glu- or NMDA-treated model group. All experiments were independently repeated at least three times.

## 5. Conclusions

In summary, nine paxilline-type IDTs, including seven previously undescribed compounds designated as tolypindoles A–G (**1**–**7**) and two known compounds (**8** and **9**), were isolated from *T. album* DWS131. Biological evaluations demonstrated that compounds **2**, **5**, **6**, **8**, and **9** possess potent neuroprotective activities against glutamate-induced excitotoxicity, with compounds **8** and **9** effectively attenuating oxidative stress and modulating ferroptosis-associated markers, including the SLC7A11/GPX4 axis and ACSL4. This work not only expands the structural diversity and pharmacological scope of IDTs but also addresses the gap in in vivo neuroprotective model studies for this class of compounds, laying an important foundation for further exploration of their mechanisms of action and the development of related lead compounds.

## Data Availability

The original contributions presented in this study are included in the article/Supplementary Materials. Further inquiries can be directed to the corresponding authors.

## Supplementary Materials

The following supporting information can be downloaded at: https://www.mdpi.com/article/10.3390/ph19060807/s1. Figures S1–S9: NMR spectra (^1^H NMR, ^13^C NMR, HSQC, HMBC, COSY, NOESY), HRESIMS, ECD and UV of **1**; Figures S10–S19: NMR spectra (^1^H NMR, ^13^C NMR, HSQC, HMBC, COSY, NOESY), HRESIMS, ECD, UV and IR of **2**; Figures S20–S28: NMR spectra (^1^H NMR, ^13^C NMR, HSQC, HMBC, COSY), HRESIMS, ECD, UV and IR of **3**; Figures S29–S38: NMR spectra (^1^H NMR, ^13^C NMR, HSQC, HMBC, COSY, NOESY), HRESIMS, ECD, UV and IR of **4**; Figures S39–S47: NMR spectra (^1^H NMR, ^13^C NMR, HSQC, HMBC, COSY, NOESY), HRESIMS, UV and IR of **5**; Figures S48–S57: NMR spectra (^1^H NMR, ^13^C NMR, HSQC, HMBC, COSY, NOESY), HRESIMS, ECD (shared with **5**), UV and IR of **6**; Figures S58–S67: NMR spectra (^1^H NMR, ^13^C NMR, HSQC, HMBC, COSY, NOESY), HRESIMS, ECD, UV and IR of **7**; Figures S68–S71: ^13^C NMR chemical shifts calculation results of two isomers of **1**–**4**; Figure S72: The viability of R28 cells treated with glutamate and 1 μM compounds **1**–**9** for 24 h; Figure S73: Cytotoxicity evaluation of compounds **8** and **9** on normal R28 cells; Figures S74–S76: Original Western blot membranes for ACSL4, GPX4 and SLC7a11 protein expression; Table S1: ^1^H NMR data for compounds **1**–**7**; Table S2: ^13^C NMR data for compounds **1**–**7**; Tables S3–S6: Experimental ^13^C NMR chemical shifts and GFN2-NMR predicted data of two possible isomers of **1**–**4**; Table S7: Experimental ^13^C NMR chemical shifts and GFN2-NMR predicted data of four possible isomers of **5**; Table S8: Experimental ^13^C NMR chemical shifts and GFN2-NMR predicted data of four possible isomers of **7**; Table S9: ITS sequence of *Tolypocladium album* DWS131.
