# Supplementary material for: Neuroprotective Indole Diterpenoids from the Fungus Tolypocladium album DWS131
Source: Pharmaceuticals (Basel). 2026 May 22;19(6):807. doi: 10.3390/ph19060807 (PMC13304572; doi:10.3390/ph19060807)
Supplement: Supplementary file 1 [file pharmaceuticals-19-00807-s001.zip › Supplementary Materials.pdf]

Supplementary materials for

Neuroprotective Indole Diterpenoids from the Fungus *Tolypocladium album* DWS131

Ai-Lin Liang[a,b,†], Chao Wang[c,d,†], Xing-Yi Chen[c,d], Yu-Feng Tan[b], Wen-Yu Lu[b], Peng-Ju Xu[b], Hong-Ping Long[e], Shao Liu[a], Jing Li[a,b,*], Wen-Xuan Wang[b,*], Xiaobo Xia[c,d*]

[a]Department of Pharmacy, National Clinical Research Center for Geriatric Disorders, Xiangya Hospital, Central South University, Changsha, Hunan 410008, PR China;

[b]Xiangya School of Pharmaceutical Sciences, Central South University, Changsha 410083, PR China

[c]Eye Center of Xiangya Hospital, Central South University, Changsha, Hunan 410083, PR China

[d]Hunan Key Laboratory of Ophthalmology, Changsha, Hunan 410008, PR China

[e]Center for Medical Research and Innovation, The First Hospital of Hunan University of Chinese Medicine, Changsha, Hunan, 410007, PR China

[†]These authors contributed equally this work.

Corresponding Authors：

[*]E-mail: lijingliyun@csu.edu.cn (Jing Li)

[*]E-mail: wangwenxuan@csu.edu.cn (Wen-Xuan Wang),

[*]E-mail: xbxia21@csu.edu.cn (Xiaobo Xia)

Table of contents

Figure S1. ^{1}H NMR spectrum of **1** (600 MHz, CD_3OD). ... 6

Figure S2. ^{13}C NMR spectrum of **1** (150 MHz, CD_3OD). ... 7

Figure S3. HSQC spectrum of **1** (600 MHz, CD_3OD). ... 7

Figure S4. HMBC spectrum of **1** (600 MHz, CD_3OD). ... 8

Figure S5. ^{1}H-^{1}H COSY spectrum of **1** (600 MHz, CD_3OD). ... 8

Figure S6. NOESY spectrum of **1** (600 MHz, CD_3OD). ... 9

Figure S7. HRESIMS spectrum of **1**. ... 9

Figure S8. Experimental ECD spectrum of **1**. ... 9

Figure S9. UV spectrum of **1**. ... 10

Figure S10. ^{1}H NMR spectrum of **2** (600 MHz, CD_3OD). ... 10

Figure S11. ^{13}C NMR spectrum of **2** (150 MHz, CD_3OD). ... 11

Figure S12. HSQC spectrum of **2** (600 MHz, CD_3OD). ... 11

Figure S13. HMBC spectrum of **2** (600 MHz, CD_3OD). ... 11

Figure S14. ^{1}H-^{1}H COSY spectrum of **2** (600 MHz, CD_3OD). ... 12

Figure S15. NOESY spectrum of **2** (600 MHz, CD_3OD). ... 13

Figure S16. HRESIM spectrum of **2** (600 MHz, CD_3OD). ... 13

Figure S17. Experimental ECD spectrum of **2**. ... 13

Figure S18. UV spectrum of **2**. ... 14

Figure S19. IR spectrum of **2**. ... 14

Figure S20. ^{1}H spectrum of **3** (600 MHz, CD_3OD). ... 15

Figure S21. ^{13}C spectrum of **3** (150 MHz, CD_3OD). ... 15

Figure S22. HSQC spectrum of **3** (600 MHz, CD_3OD). ... 15

Figure S23. HMBC spectrum of **3** (600 MHz, CD_3OD). ... 16

Figure S24. ^{1}H-^{1}H COSY spectrum of **3** (600 MHz, CD_3OD). ... 17

Figure S25. HRESIMS spectrum of **3**. ... 17

Figure S26. Experimental ECD spectrum of **3**. ... 17

Figure S27. UV spectrum of **3**. 18

Figure S28. IR spectrum of **3**. 18

Figure S29. ^{1}H spectrum of **4** (600 MHz, CD_3OD). 19

Figure S30. ^{13}C spectrum of **4** (150 MHz, CD_3OD). 19

Figure S31. HSQC spectrum of **4** (600 MHz, CD3OD). 20

Figure S32. HMBC spectrum of **4** (600 MHz, CD_3OD). 20

Figure S33. ^{1}H-^{1}H spectrum of **4** (600 MHz, CD_3OD). 21

Figure S34. NOESY spectrum of **4** (600 MHz, CD_3OD). 21

Figure S35. HRESIMS spectrum of **4** 21

Figure S36. Experimental ECD spectrum of **4**. 22

Figure S37. UV spectrum of **4**. 22

Figure S38. IR spectrum of **4** 23

Figure S39. ^{1}H spectrum of **5** (600 MHz, CD_3OD). 23

Figure S40. ^{13}C spectrum of **5** (150 MHz, CD_3OD). 24

Figure S41. HSQC spectrum of **5** (600 MHz, CD_3OD). 24

Figure S42. HMBC spectrum of **5** (600 MHz, CD_3OD). 25

Figure S43. ^{1}H-^{1}H spectrum of **5** (600 MHz, CD_3OD). 25

Figure S44. NOESY spectrum of **5** (600 MHz, CD_3OD). 26

Figure S45. HRESIMS spectrum of **5**. 26

Figure S46. UV spectrum of **5**. 26

Figure S47. IR spectrum of **5**. 26

Figure S48. ^{1}H spectrum of **6** (600 MHz, CD_3OD). 27

Figure S49. ^{13}C spectrum of **6** (150 MHz, CD_3OD). 28

Figure S50. HSQC spectrum of **6** (600 MHz, CD_3OD). 28

Figure S51. HMBC spectrum of **6** (600 MHz, CD_3OD). 29

Figure S52. ^{1}H-^{1}H spectrum of **6** (600 MHz, CD_3OD). 29

Figure S53. NOESY spectrum of **6** (600 MHz, CD_3OD). 30

Figure S54. HRESIMS spectrum of **6**. 30

Figure S55. Experimental ECD spectra of compounds **5** and **6**. 30

Figure S56. UV spectrum of **6**. 31

Figure S57. IR spectrum of **6**. 31

Figure S58. ^{1}H spectrum of **7** (600 MHz, $CDCl_3$). 32

Figure S59. ^{13}C spectrum of **7** (150 MHz, $CDCl_3$). 32

Figure S60. HSQC spectrum of **7** (600 MHz, $CDCl_3$). 33

Figure S61. HMBC spectrum of **7** (600 MHz, $CDCl_3$). 33

Figure S62. ^{1}H-^{1}H spectrum of **7** (600 MHz, $CDCl_3$). 34

Figure S63. NOESY spectrum of **7** (600 MHz, $CDCl_3$). 34

Figure S64. HRESIMS spectrum of **7.** 34

Figure S65. Experimental ECD of **7.** 35

Figure S66. UV spectrum of **7**. 35

Figure S67. IR spectrum of **7**. 35

Figure S68. The ^{13}C NMR chemical shifts calculation results of two isomers of **1** (flexible side chain was simplified) 35

Figure S69. The ^{13}C NMR chemical shifts calculation results of two isomers of **2** (flexible side chain was simplified) 36

Figure S70. The ^{13}C NMR chemical shifts calculation results of two isomers of **3** (flexible side chain was simplified) 36

Figure S71. The ^{13}C NMR chemical shifts calculation results of two isomers of **4** (flexible side chain was simplified) 36

Figure S72. The viability of R28 cells treated with glutamate and 1 μM compounds **1**–**9** for 24 h (n = 3). 37

Figure S73. Cytotoxicity evaluation of compounds **8** and **9** on normal R28 cells (n = 3). 37

Figure S74. Original Western blot membranes for ACSL4 protein expression. 37

Figure S75. Original Western blot membranes for GPX4 protein expression. 38

Figure S76. Original Western blot membranes for SLC7a11 protein expression. 38

Table S1. ^{1}H data for compounds **1**–**7** (600 MHz, *δ* in ppm, *J* in Hz). 39

Table S2. ^{13}C NMR data for compounds **1**–**7** (150 MHz, *δ* in ppm). 41

Table S3. The experimental ^{13}C NMR chemical shifts of compound **1** and the GFN2NMR-predicted data of two possible isomers *31S*-**1a** and *31R*-**1a**. 41

Table S4. The experimental ^{13}C NMR chemical shifts of compound **2** and the GFN2NMR-

predicted data of two possible isomers *31S*-**2a** and *31R*-**2a**. 42

Table S5. The experimental ^{13}C NMR chemical shifts of compound **3** and the GFN2NMR-predicted data of two possible isomers *31S*-**3a** and *31R*-**3a**. 43

Table S6. The experimental ^{13}C NMR chemical shifts of compound **4** and the GFN2NMR-predicted data of two possible isomers *31S*-**4a** and *31R*-**4a**. 44

Table S7. The experimental ^{13}C NMR chemical shifts of compound **5** and the GFN2NMR-predicted data of four possible isomers 31*S*,36*S*-**5**, 31*S*,36*R*-**5,** 31*R*,36*R*-**5,** and 31*R*,36*S*-**5,** respectively. 44

Table S8. The experimental ^{13}C NMR chemical shifts of compound **7** and the GFN2NMR-predicted data of four possible isomers 31*S*,36*S*-**7**, 31*S*,36*R*-**7,** 31*R*,36*R*-**7,** and 31*R*,36*S*-**7,** respectively. 45

Table S9. ITS sequence of *Tolypocladium album* DWS131 46

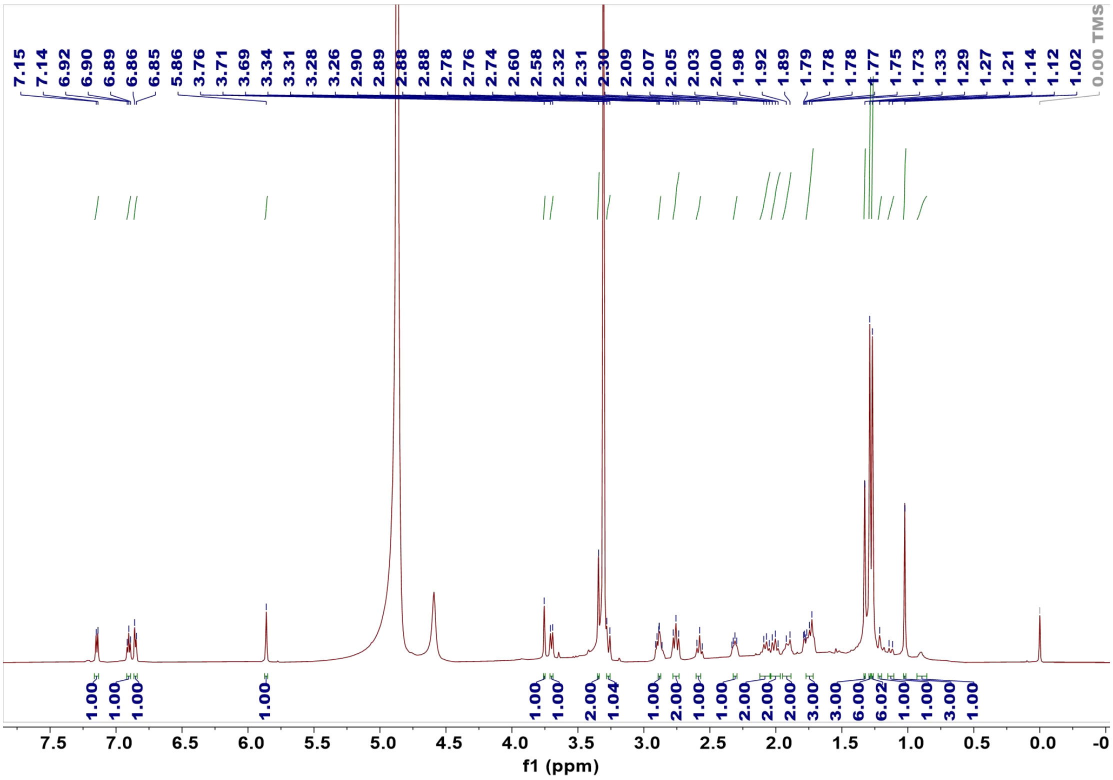

Figure S1. ^{1}H NMR spectrum of **1** (600 MHz, CD_3OD).

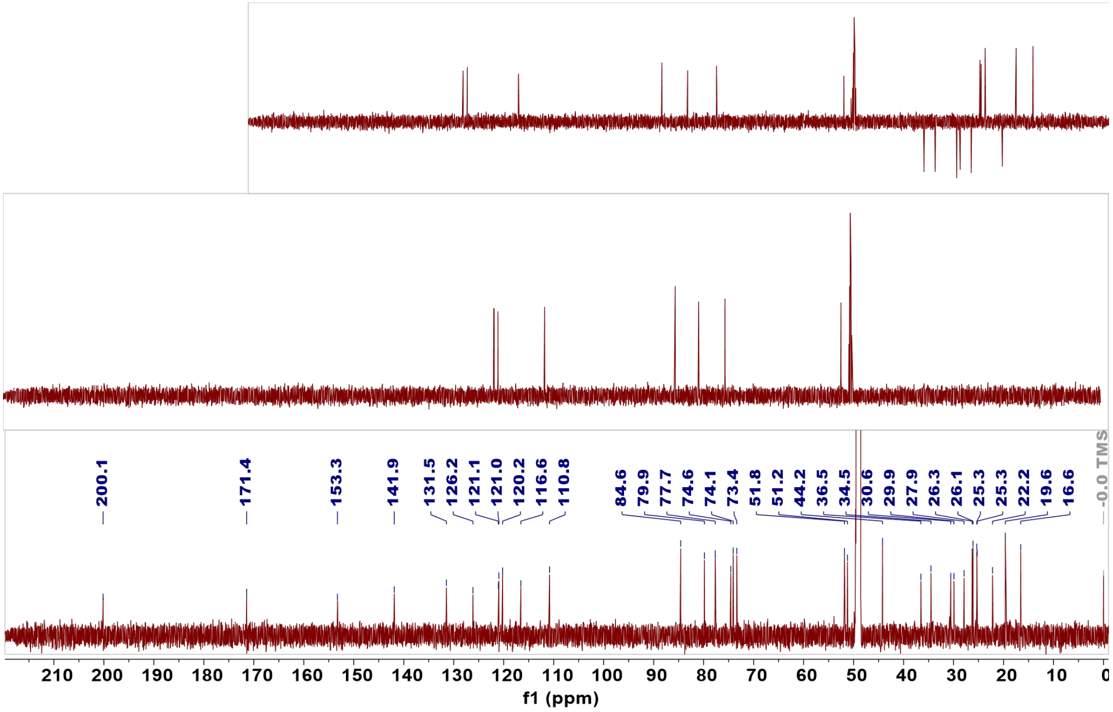

Figure S2. ^{13}C NMR spectrum of **1** (150 MHz, CD_3OD).

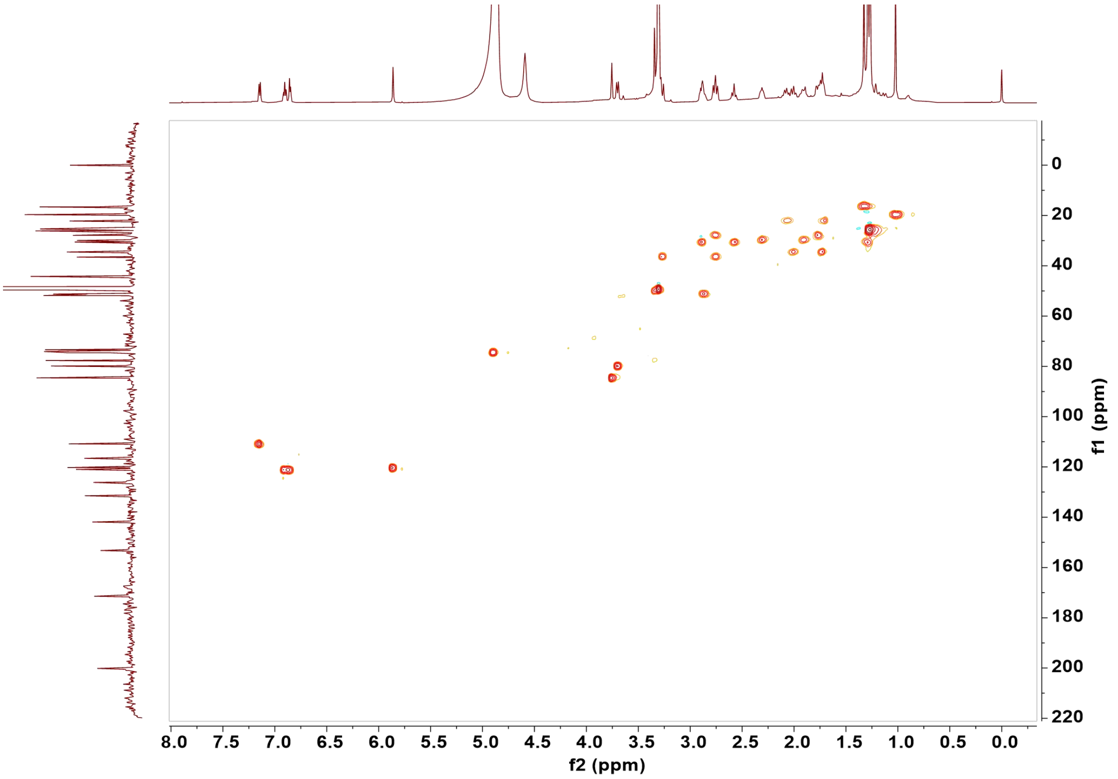

Figure S3. HSQC spectrum of **1** (600 MHz, CD_3OD).

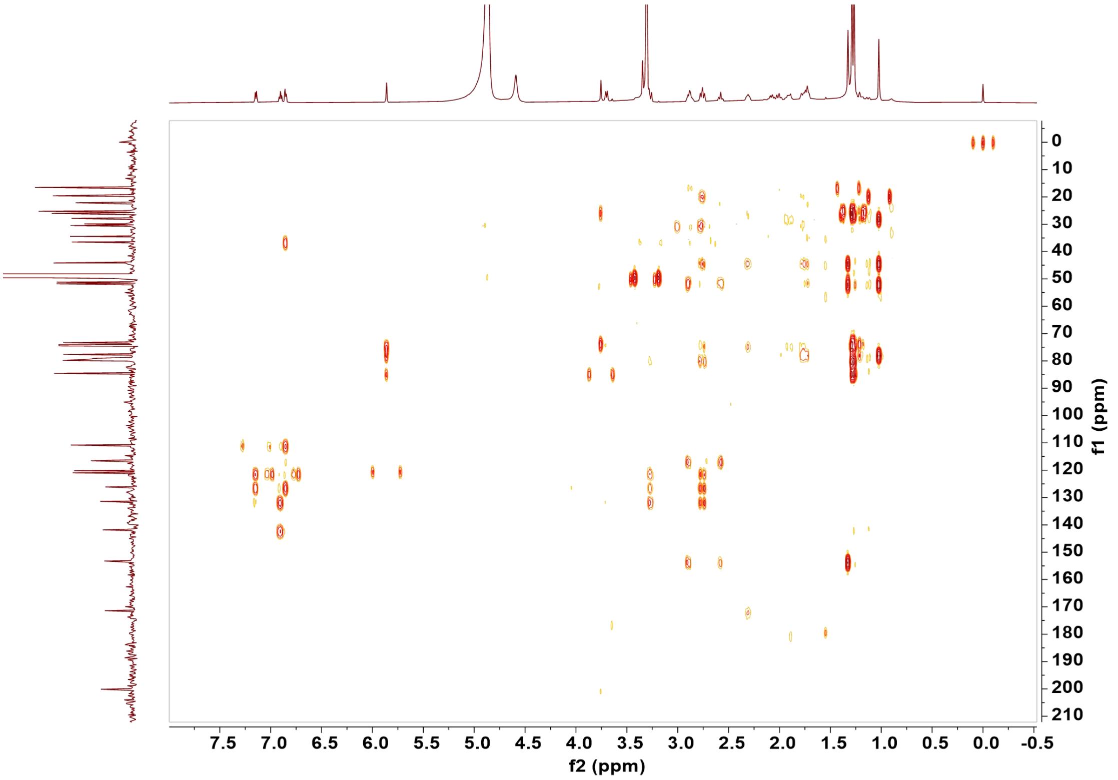

Figure S4. HMBC spectrum of **1** (600 MHz, CD_3OD).

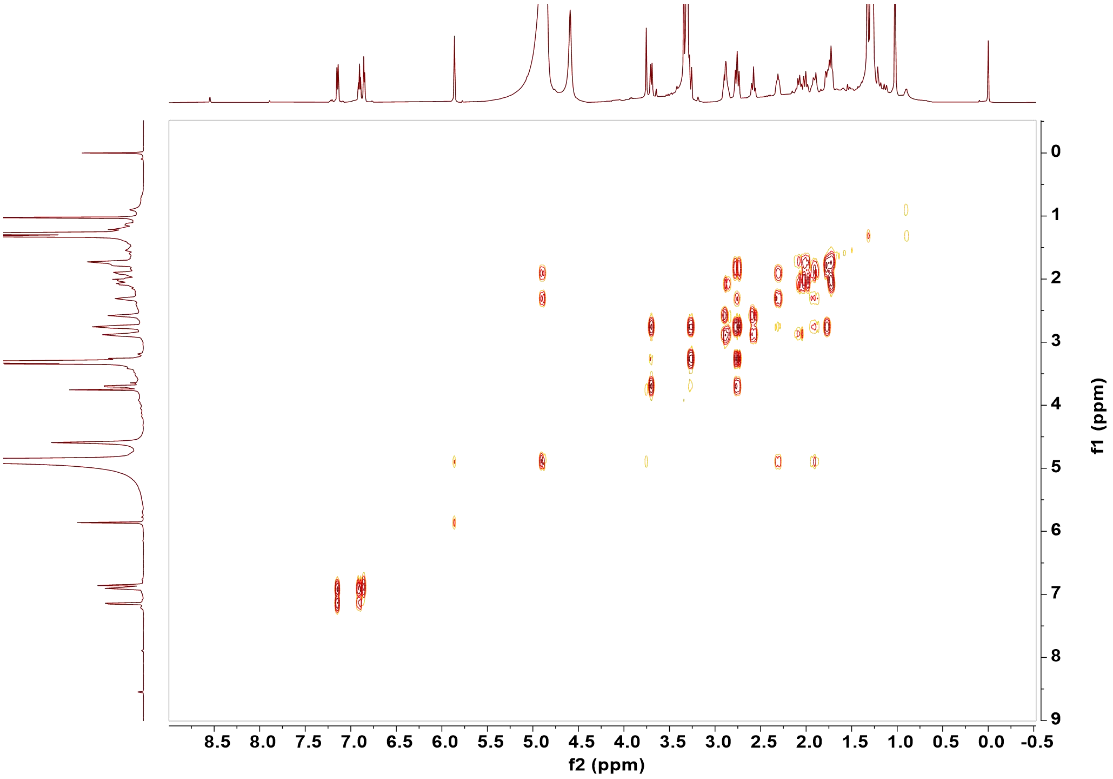

Figure S5. ^1H-^1H COSY spectrum of **1** (600 MHz, CD_3OD).

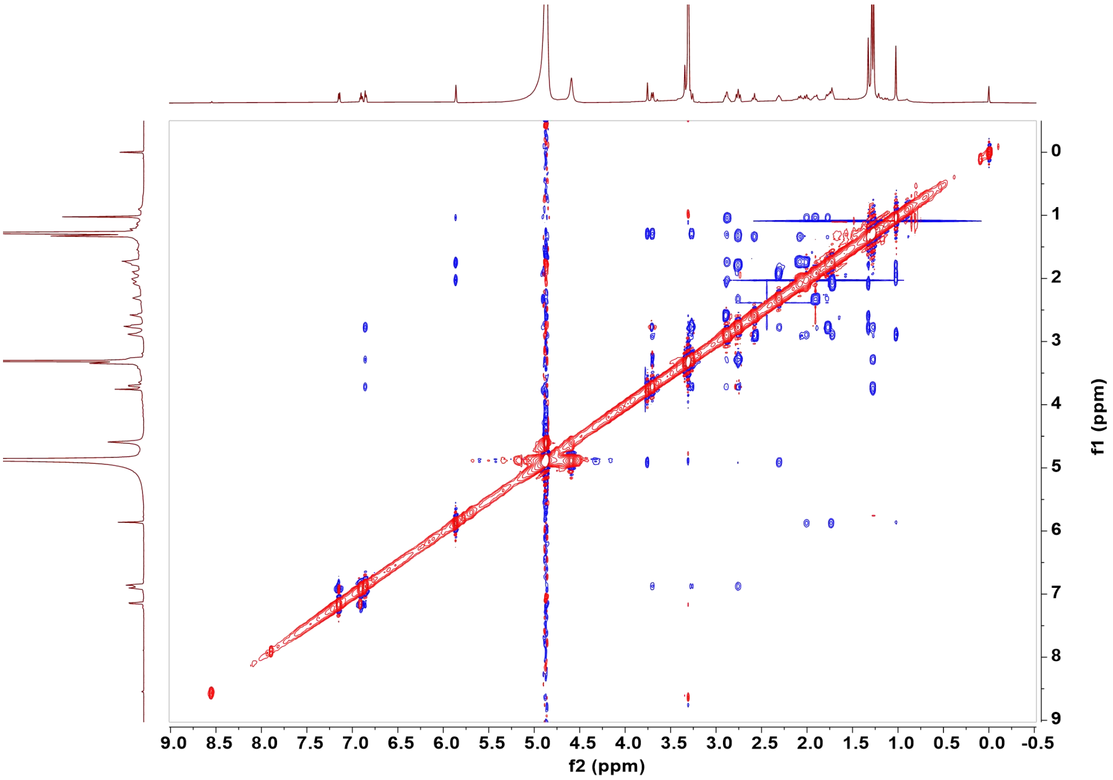

Figure S6. NOESY spectrum of **1** (600 MHz, CD_3OD).

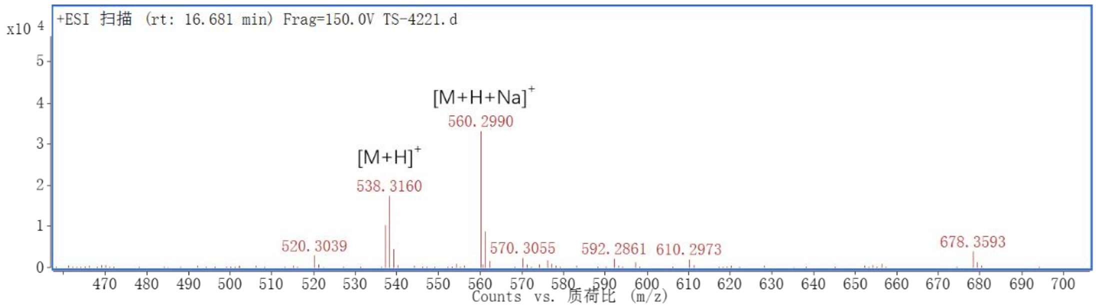

Figure S7. HRESIMS spectrum of **1**.

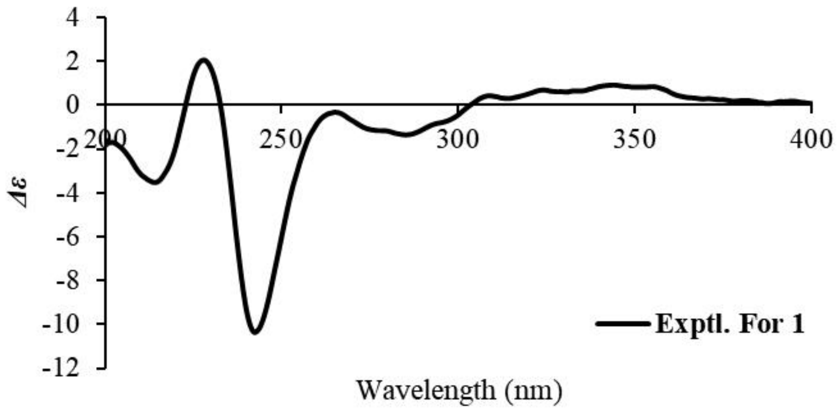

Figure S8. Experimental ECD spectrum of **1**.

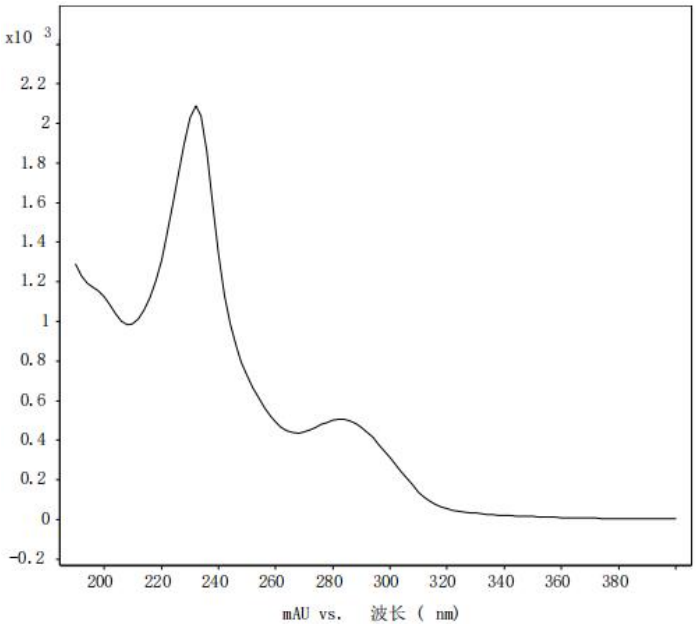

Figure S9. UV spectrum of **1**.

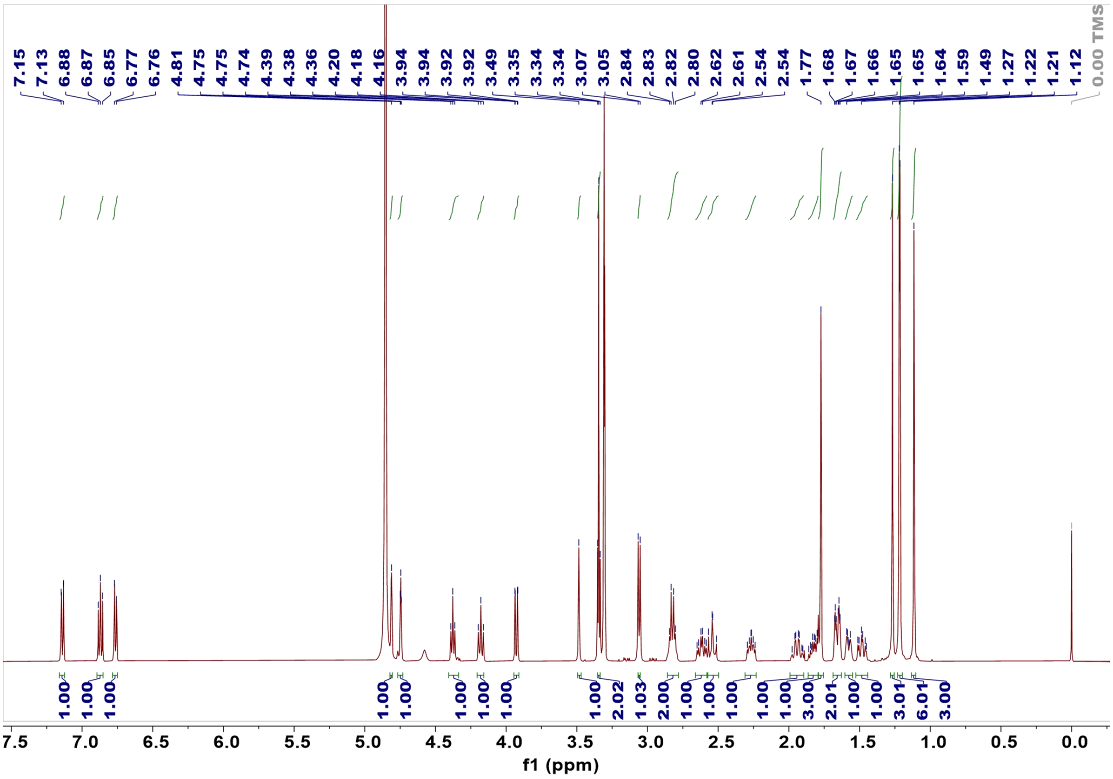

Figure S10. ^{1}H NMR spectrum of **2** (600 MHz, CD_3OD).

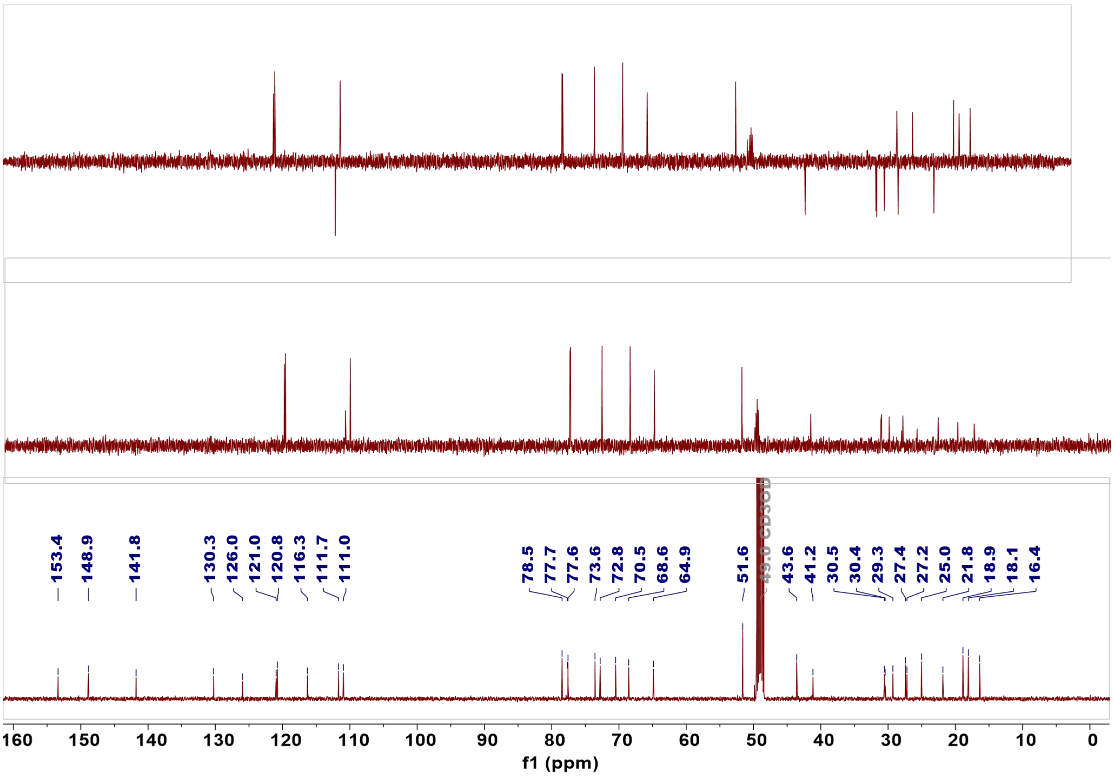

Figure S11. ^{13}C NMR spectrum of **2** (150 MHz, CD_3OD).

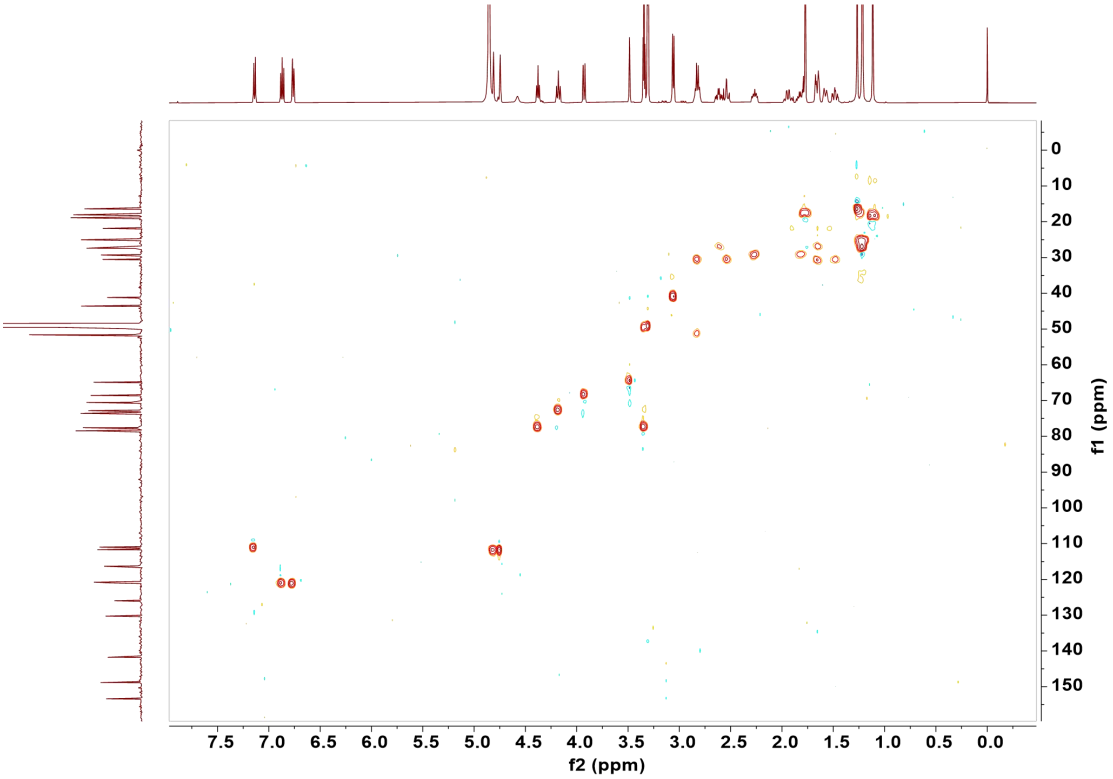

Figure S12. HSQC spectrum of **2** (600 MHz, CD_3OD).

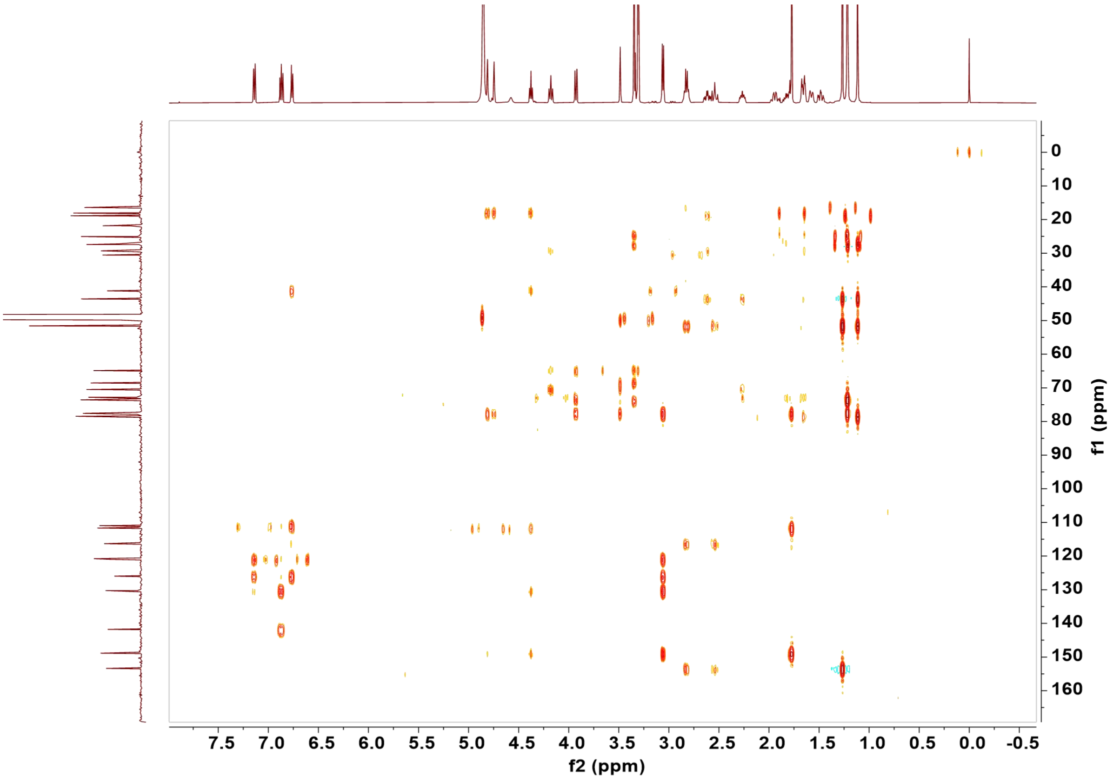

Figure S13. HMBC spectrum of **2** (600 MHz, CD_3OD).

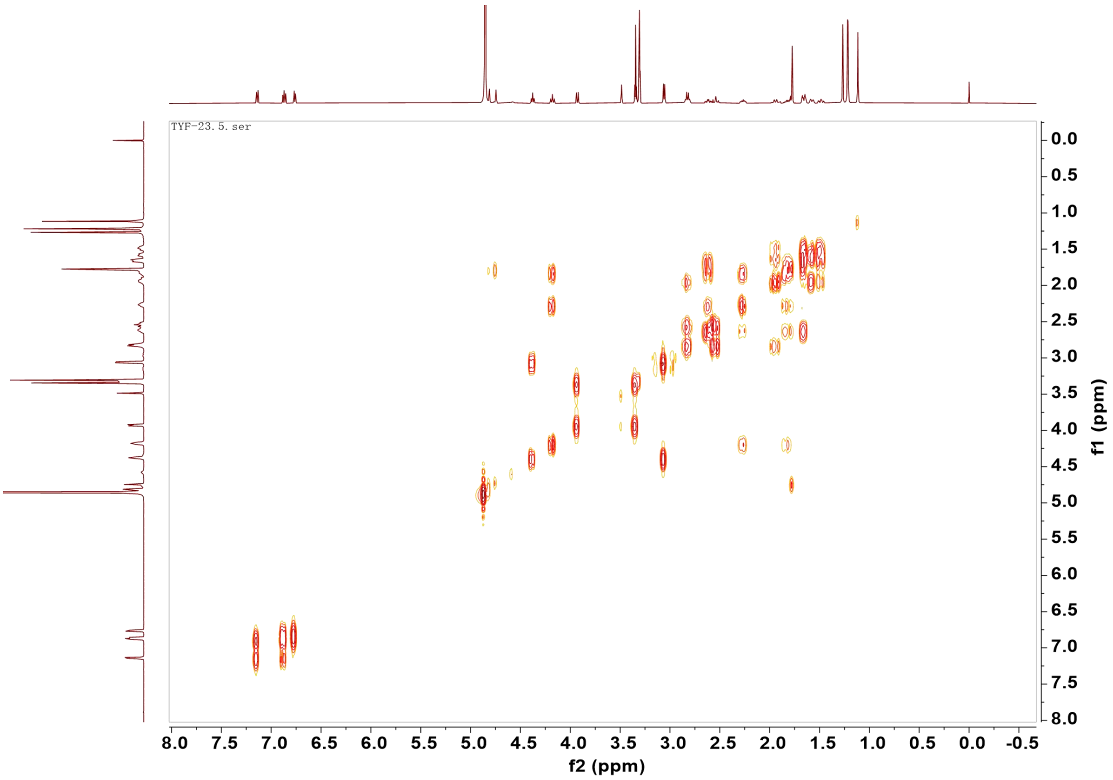

Figure S14. ^{1}H-^{1}H COSY spectrum of **2** (600 MHz, CD_3OD).

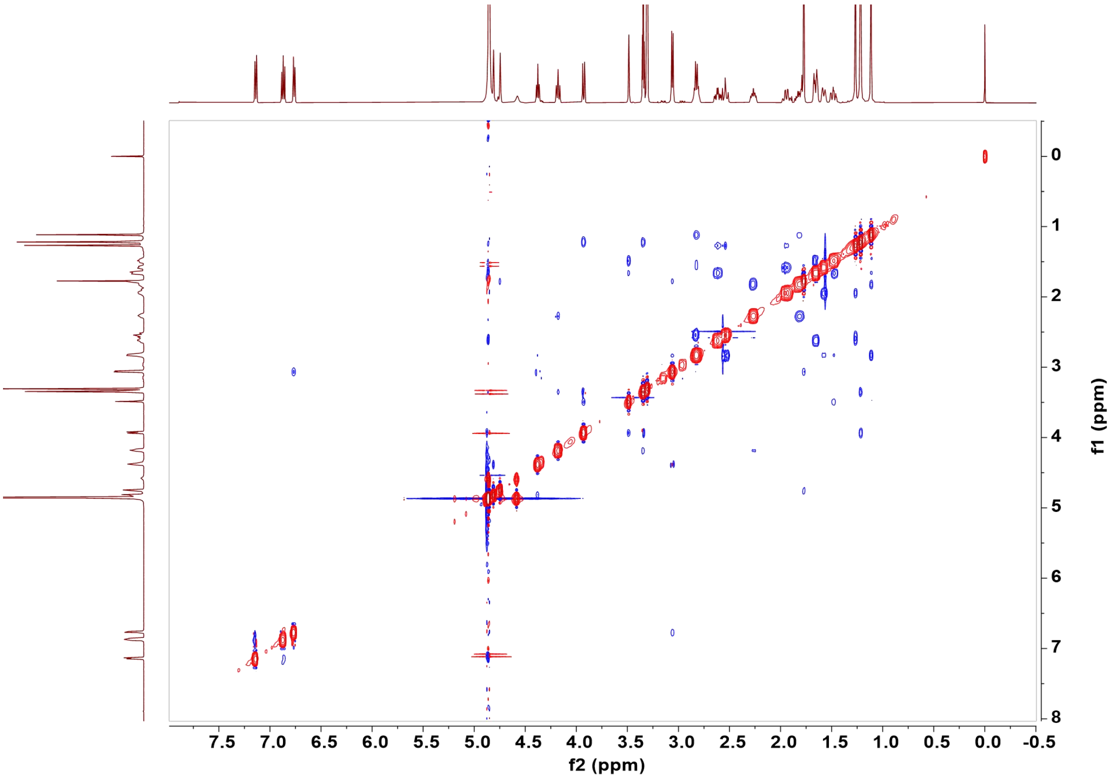

Figure S15. NOESY spectrum of **2** (600 MHz, CD_3OD).

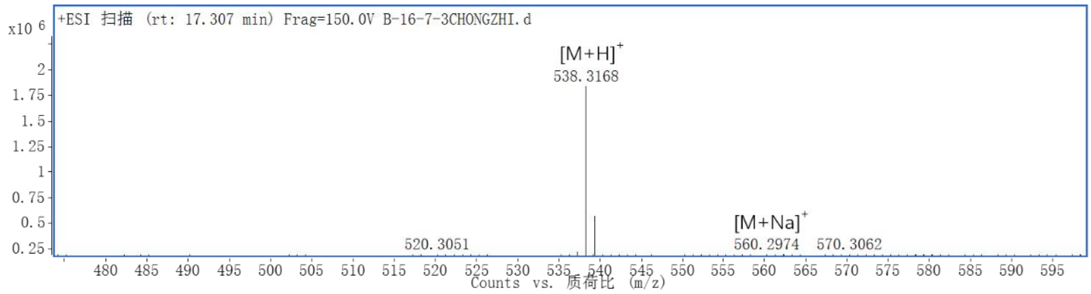

Figure S16. HRESIM spectrum of **2** (600 MHz, CD_3OD).

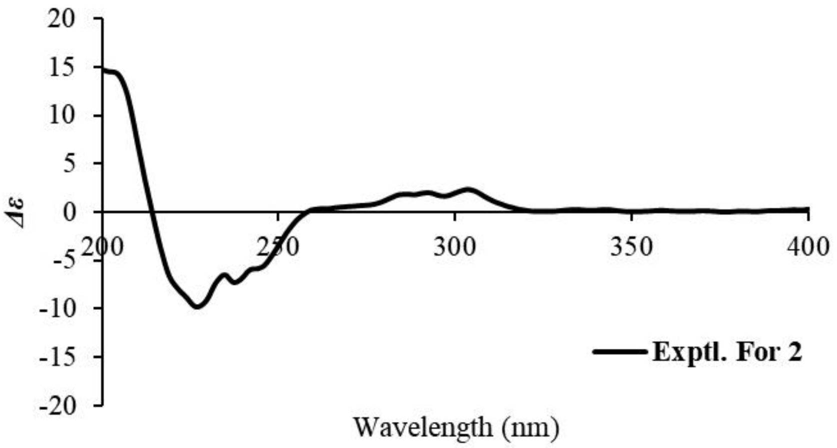

Figure S17. Experimental ECD spectrum of **2**.

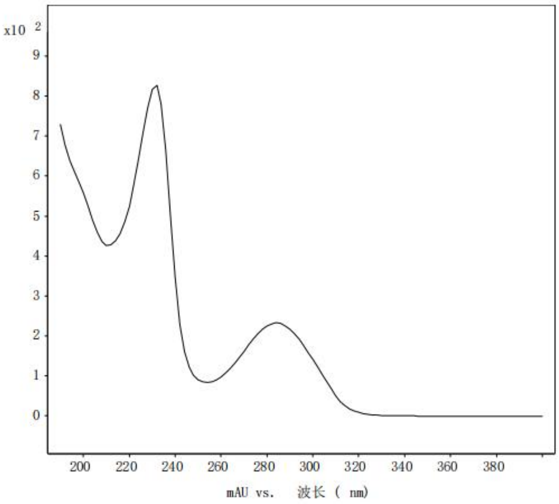

Figure S18. UV spectrum of **2**.

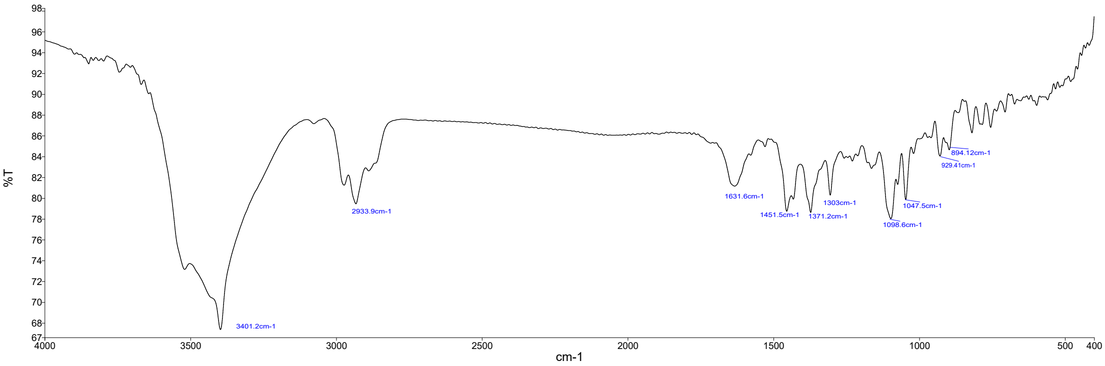

Figure S19. IR spectrum of **2**.

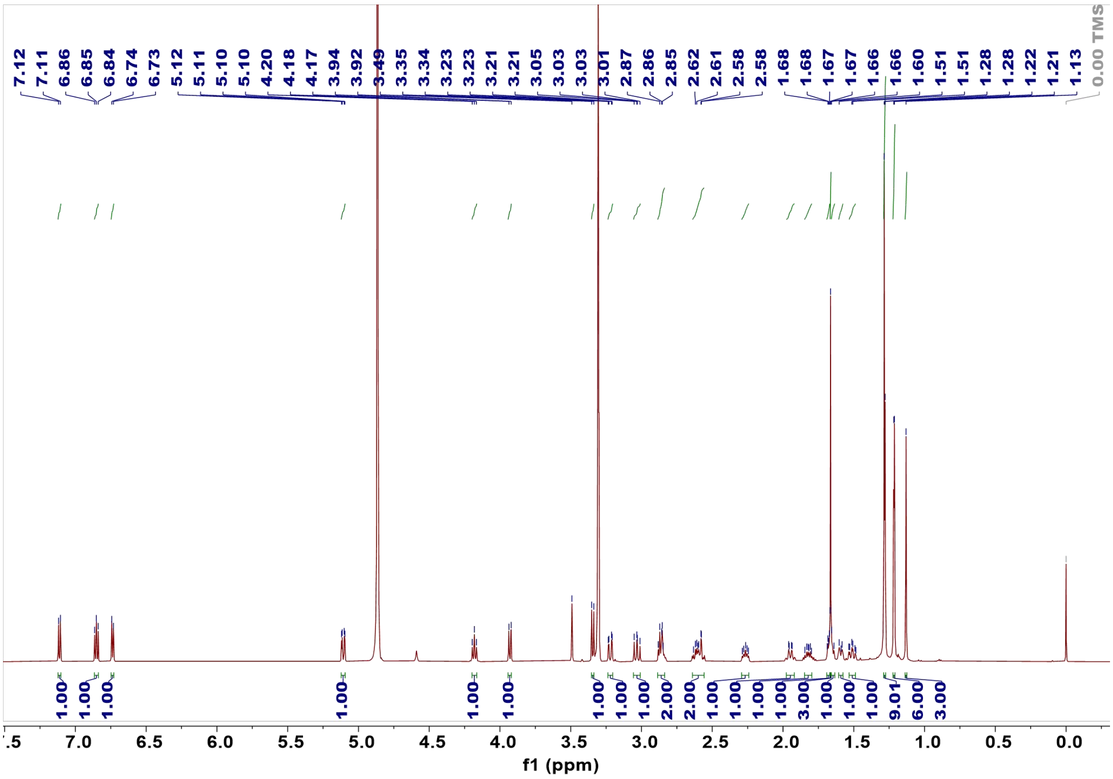

Figure S20. ^{1}H spectrum of **3** (600 MHz, CD_3OD).

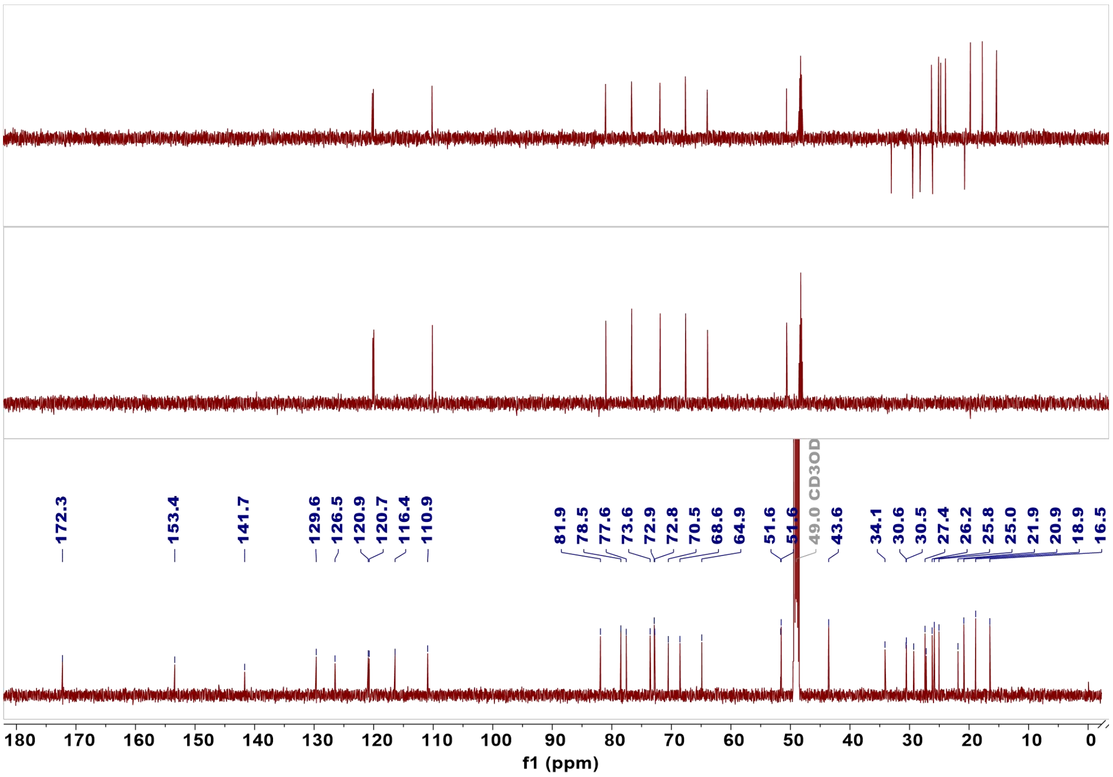

Figure S21. ^{13}C spectrum of **3** (150 MHz, CD_3OD).

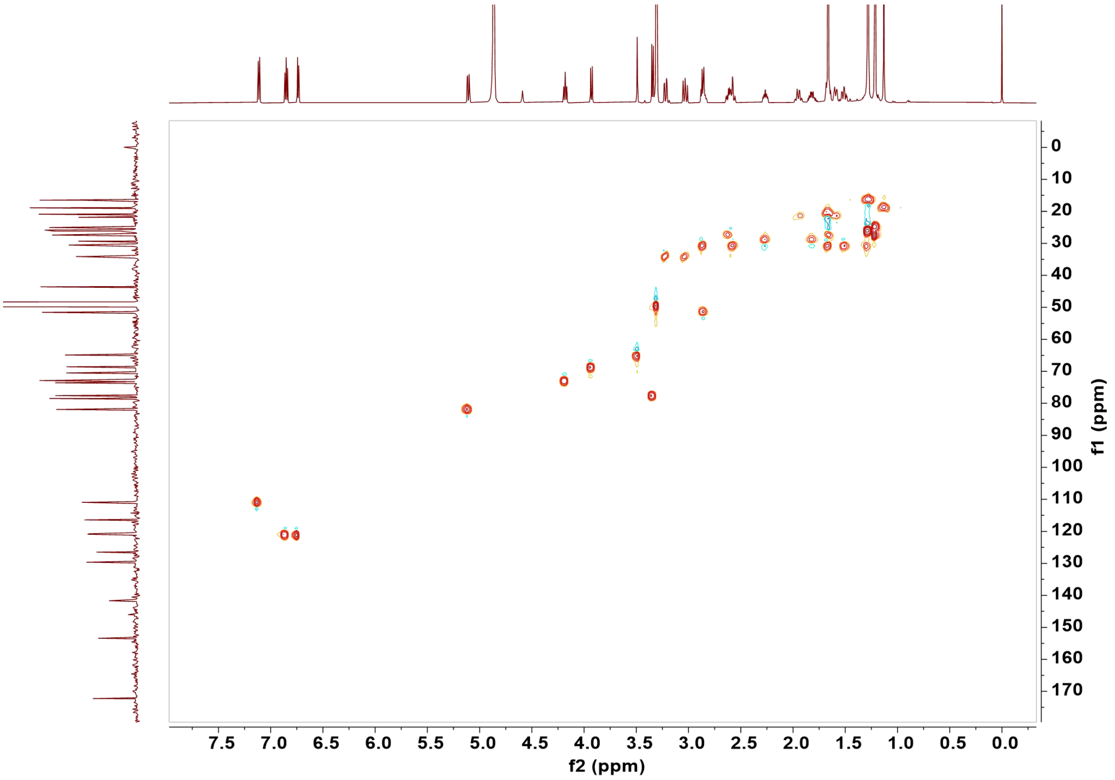

Figure S22. HSQC spectrum of **3** (600 MHz, CD_3OD).

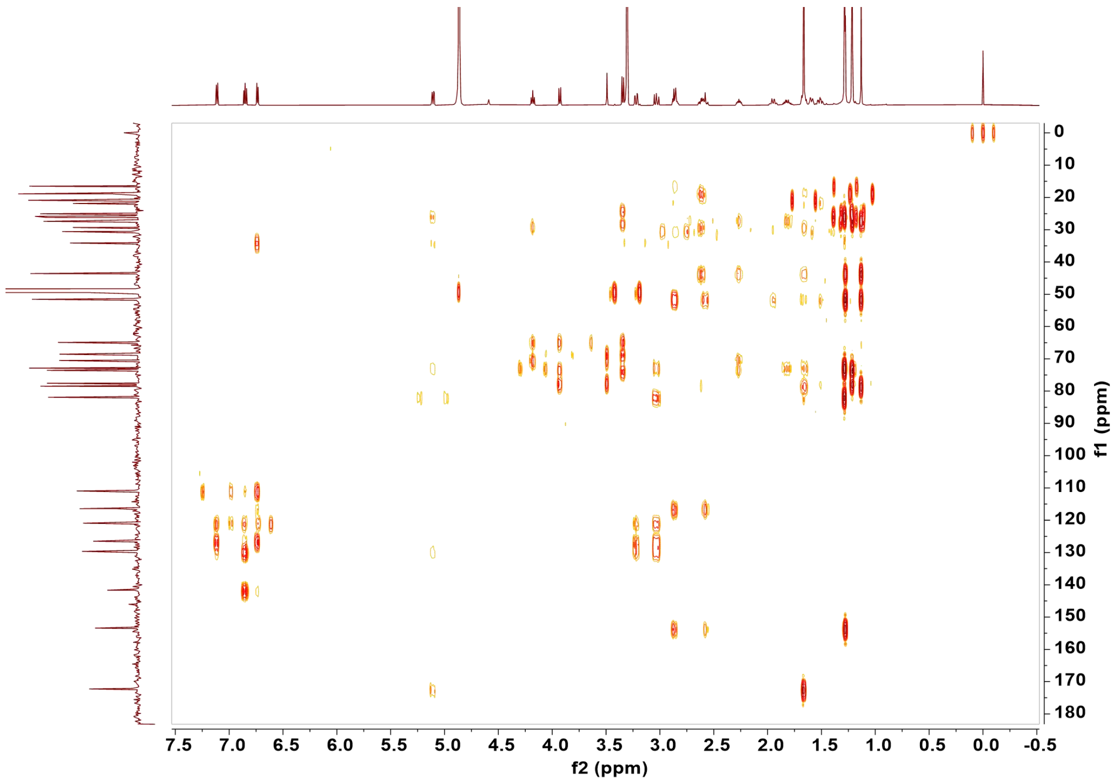

Figure S23. HMBC spectrum of **3** (600 MHz, CD_3OD).

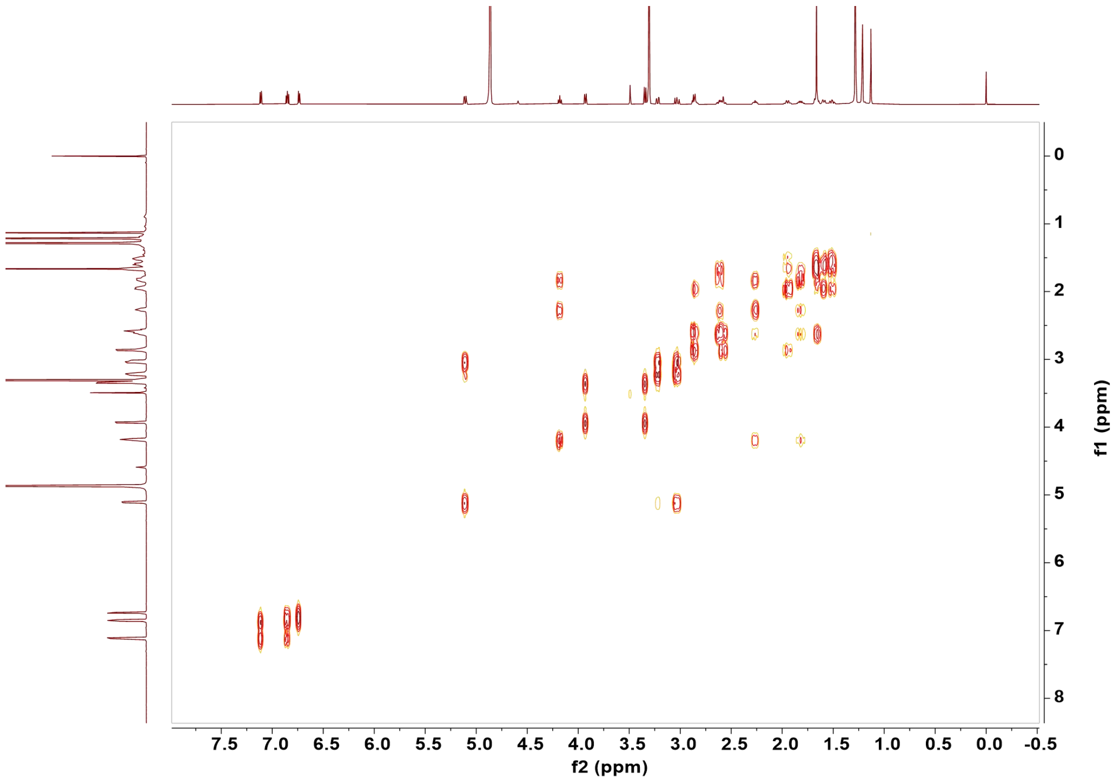

Figure S24. ^1H-^1H COSY spectrum of **3** (600 MHz, CD_3OD).

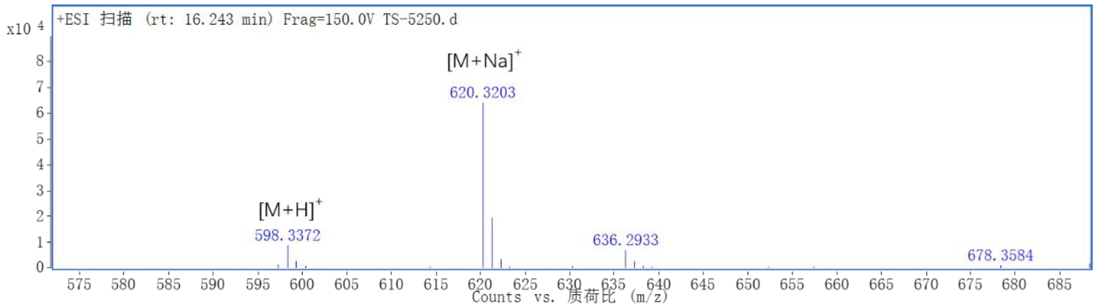

Figure S25. HRESIMS spectrum of **3**.

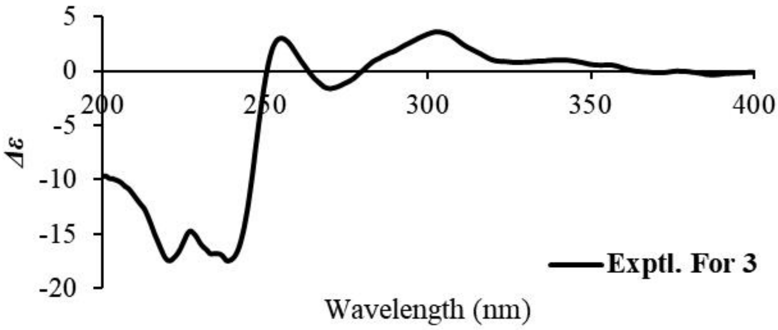

Figure S26. Experimental ECD spectrum of **3**.

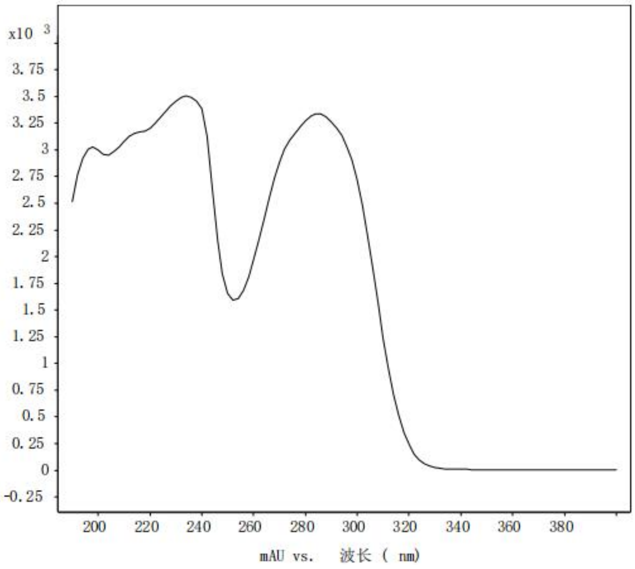

Figure S27. UV spectrum of **3**.

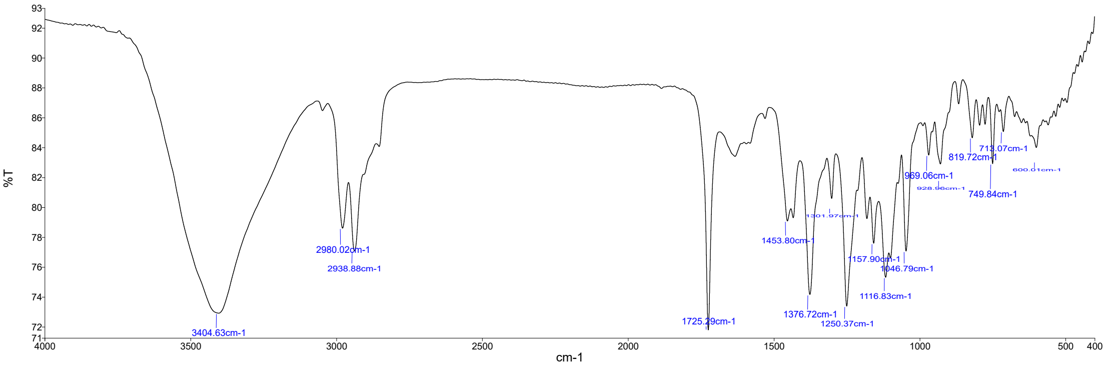

Figure S28. IR spectrum of **3**.

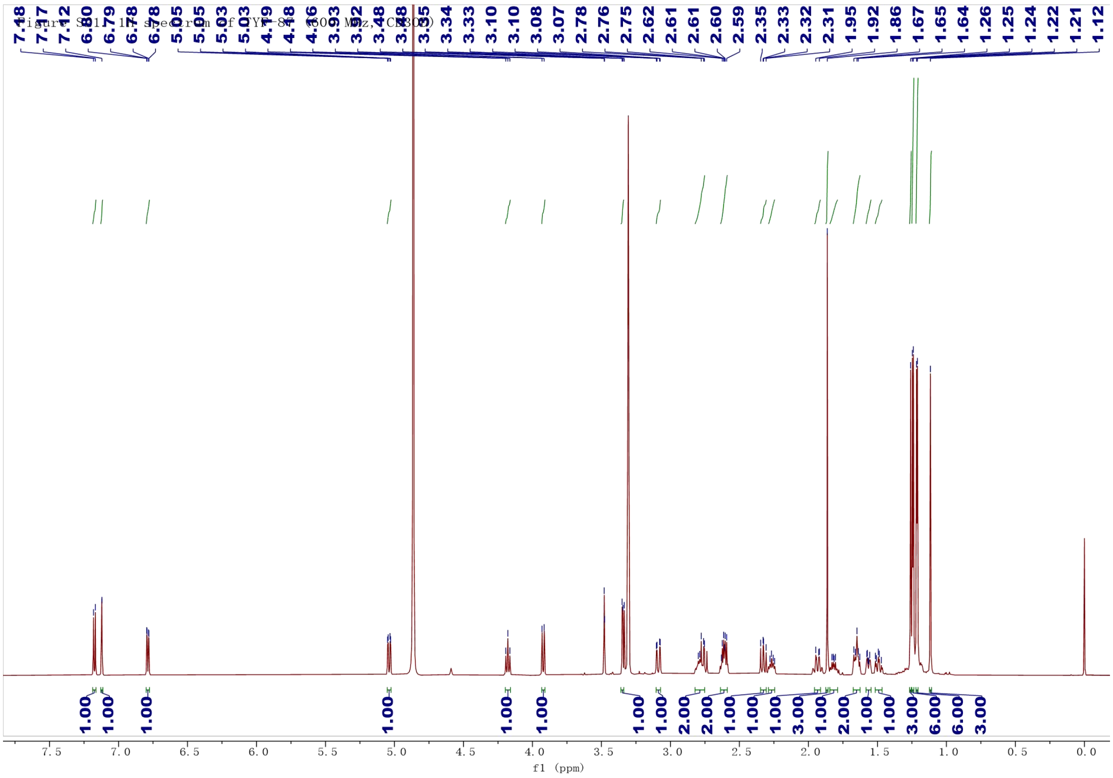

Figure S29. ^{1}H spectrum of **4** (600 MHz, CD_3OD).

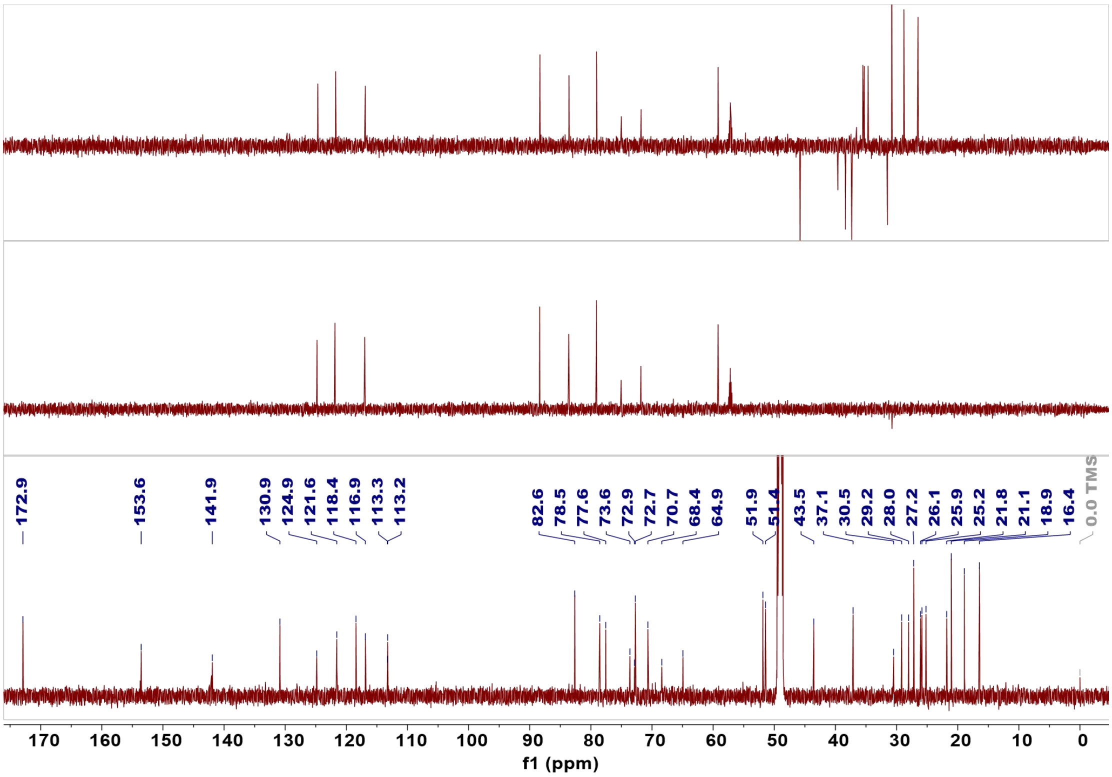

Figure S30. ^{13}C spectrum of **4** (150 MHz, CD_3OD).

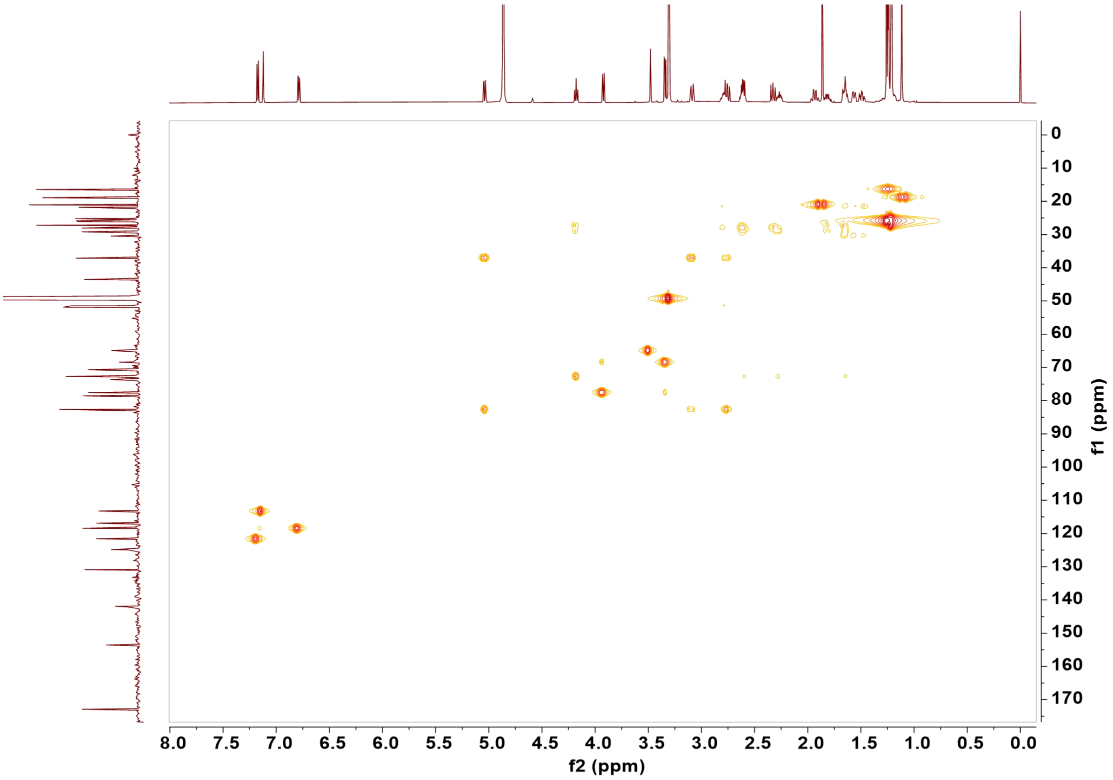

Figure S31. HSQC spectrum of **4** (600 MHz, CD3OD).

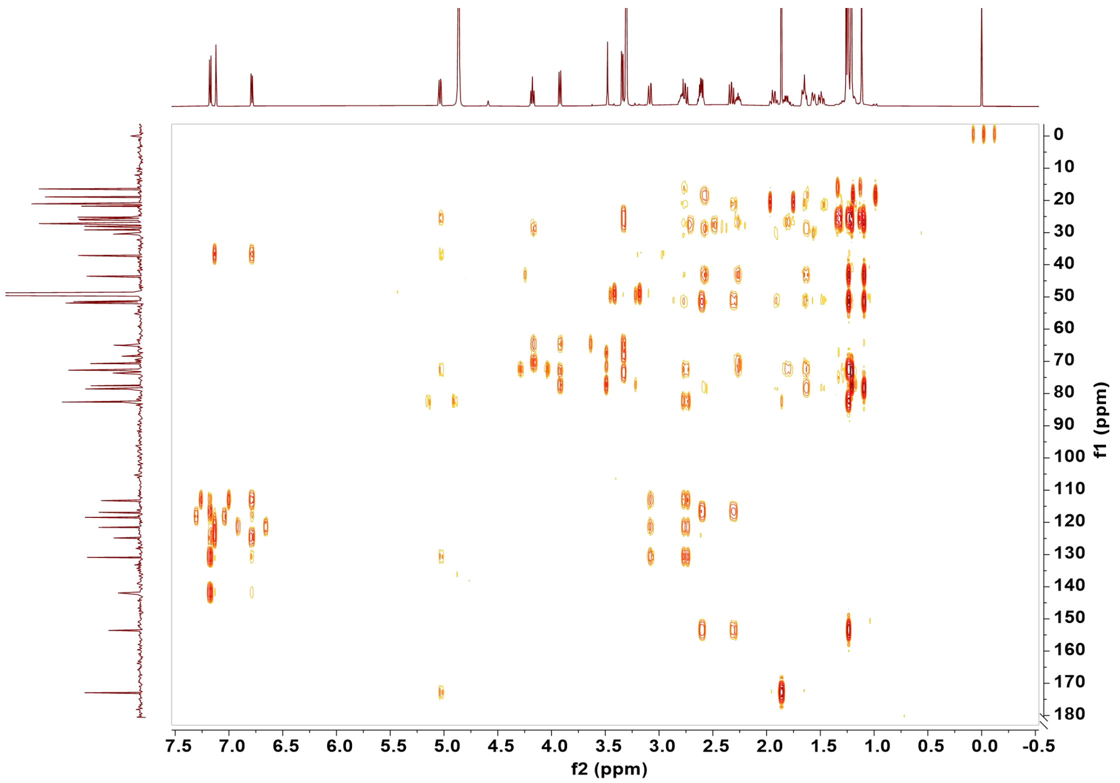

Figure S32. HMBC spectrum of **4** (600 MHz, CD_3OD).

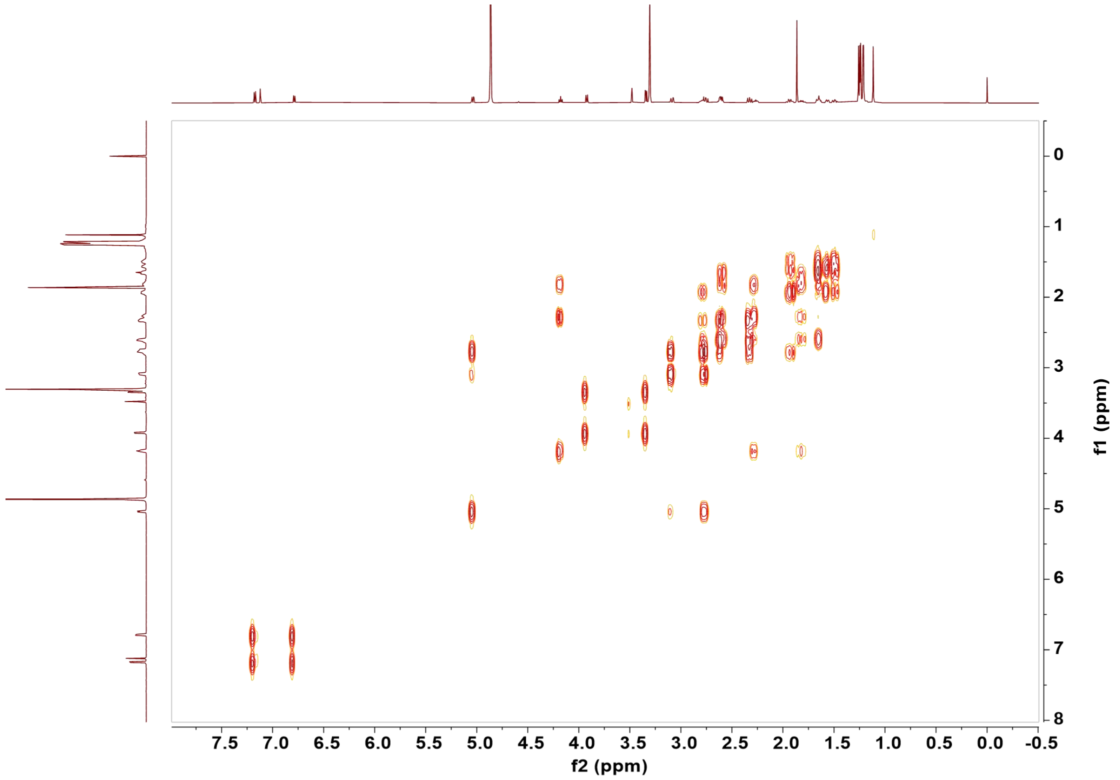

Figure S33. ^{1}H-^{1}H spectrum of **4** (600 MHz, CD_3OD).

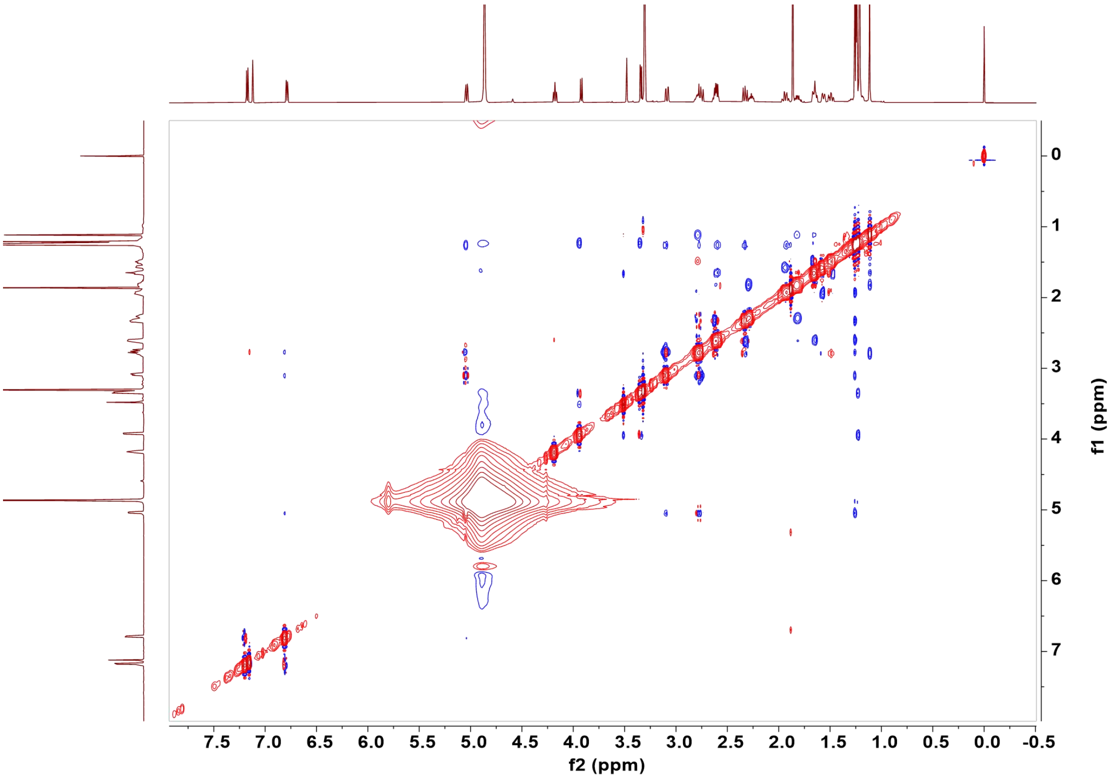

Figure S34. NOESY spectrum of **4** (600 MHz, CD_3OD).

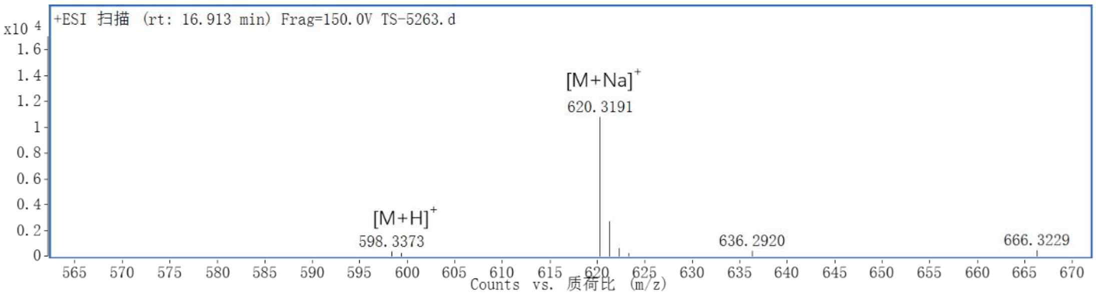

Figure S35. HRESIMS spectrum of **4**

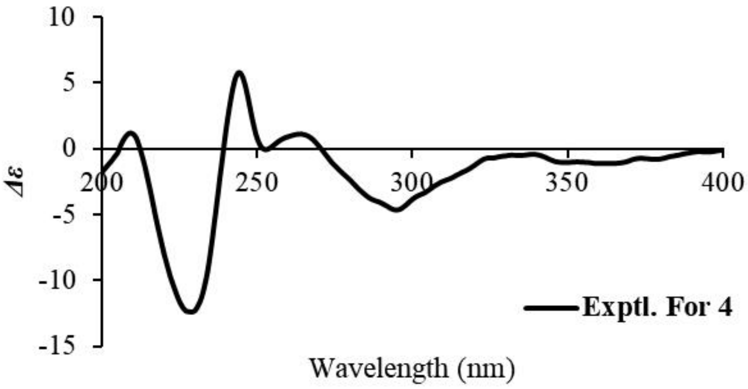

Figure S36. Experimental ECD spectrum of **4**.

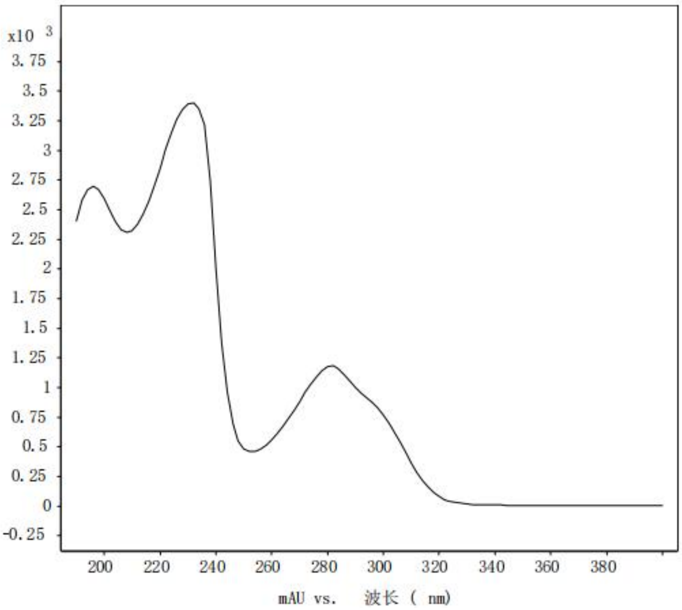

Figure S37. UV spectrum of **4**.

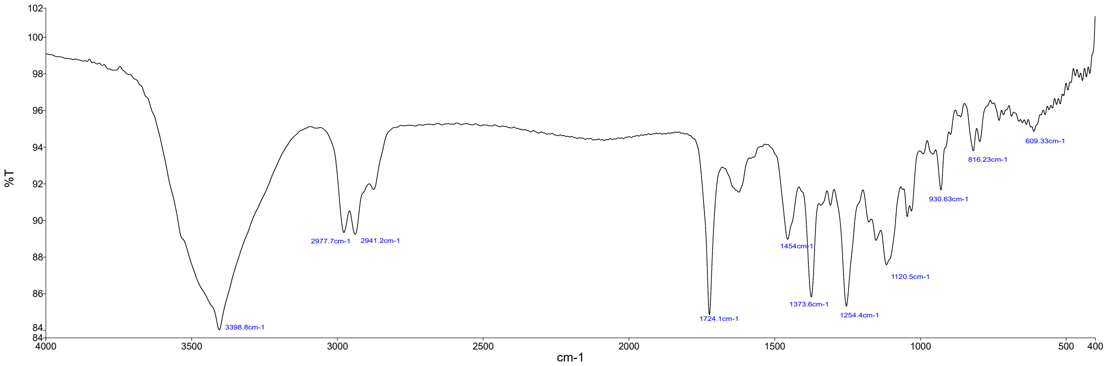

Figure S38. IR spectrum of **4**

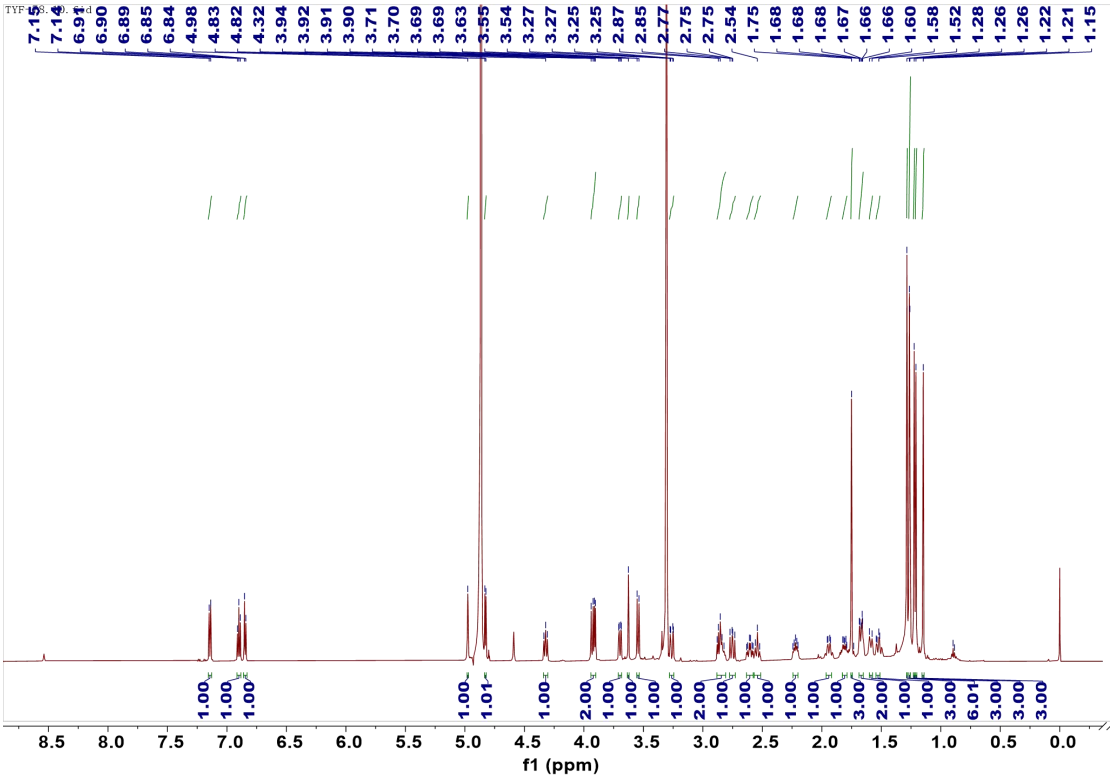

Figure S39. ^{1}H spectrum of **5** (600 MHz, CD_3OD).

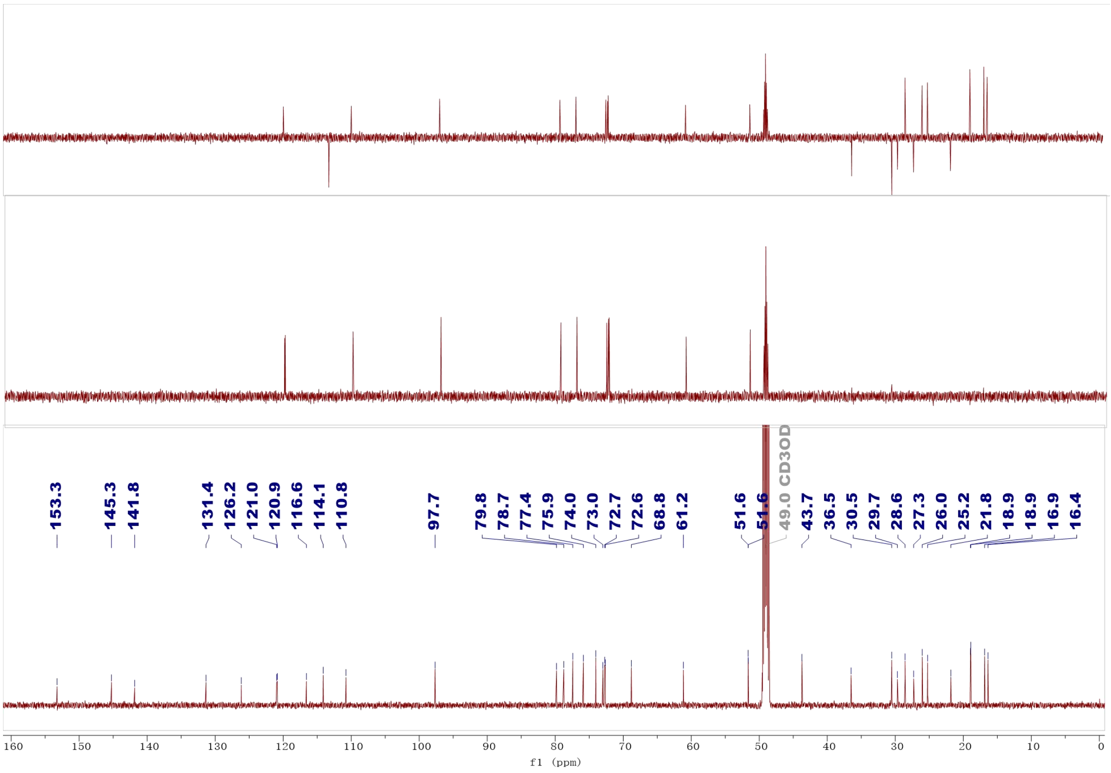

Figure S40. ^{13}C spectrum of **5** (150 MHz, CD_3OD).

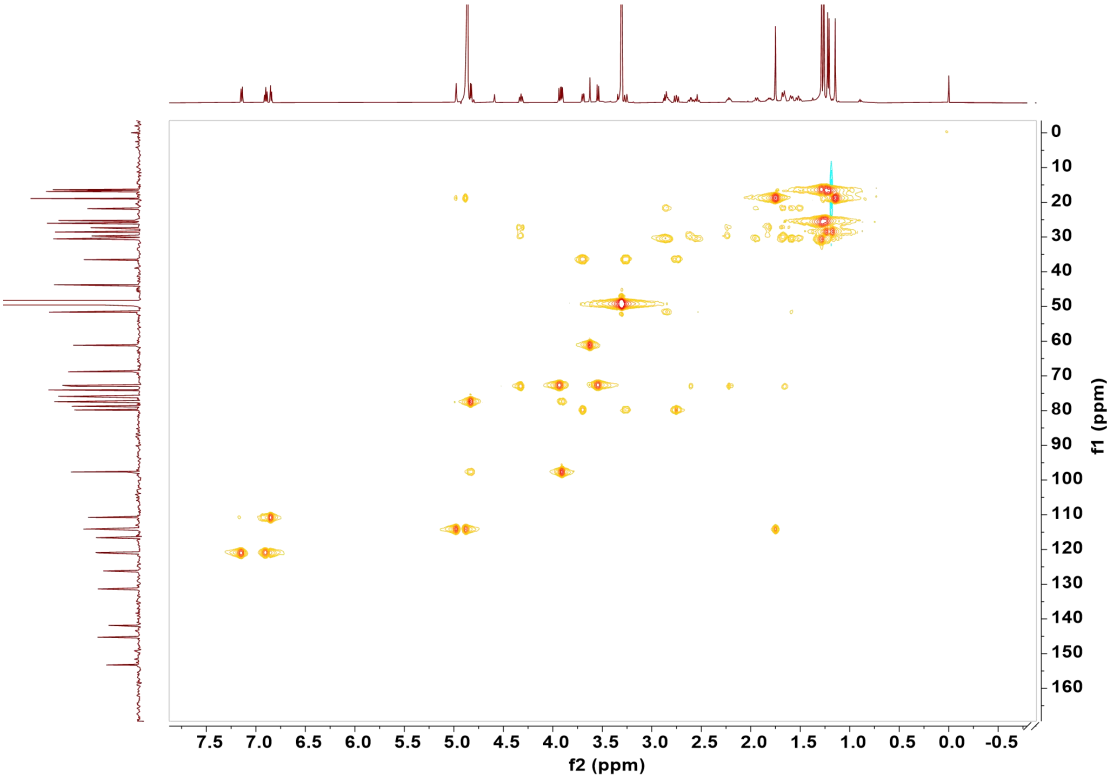

Figure S41. HSQC spectrum of **5** (600 MHz, CD_3OD).

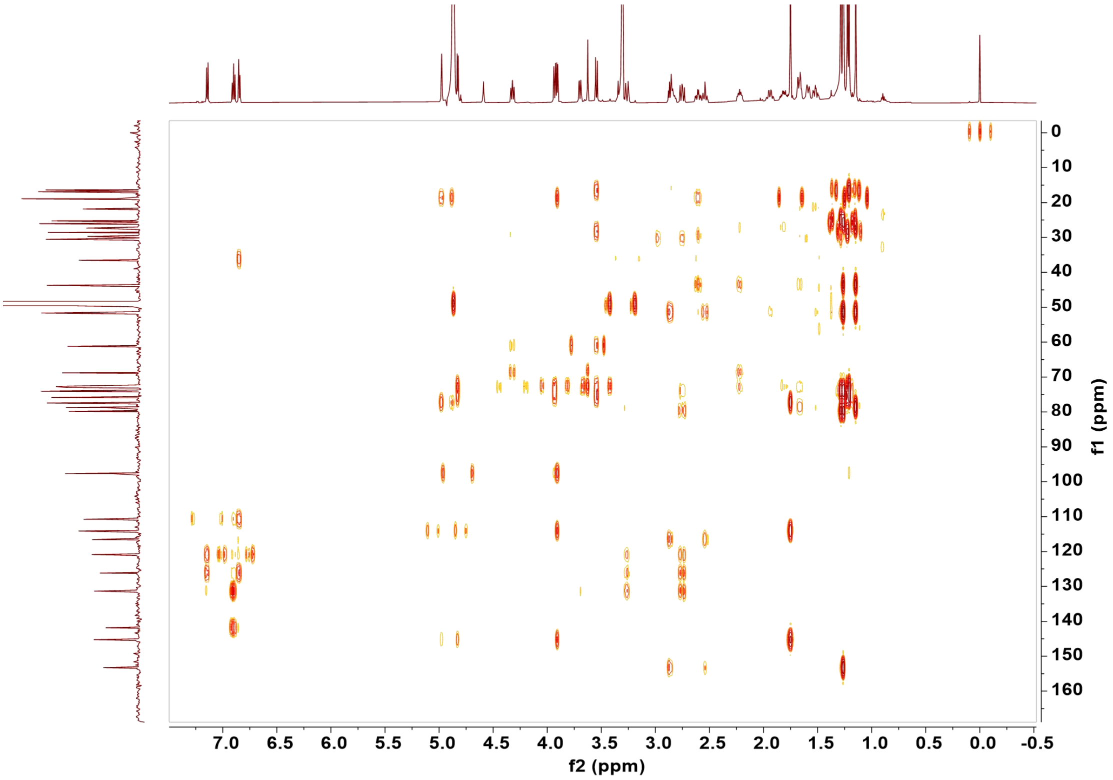

Figure S42. HMBC spectrum of **5** (600 MHz, CD_3OD).

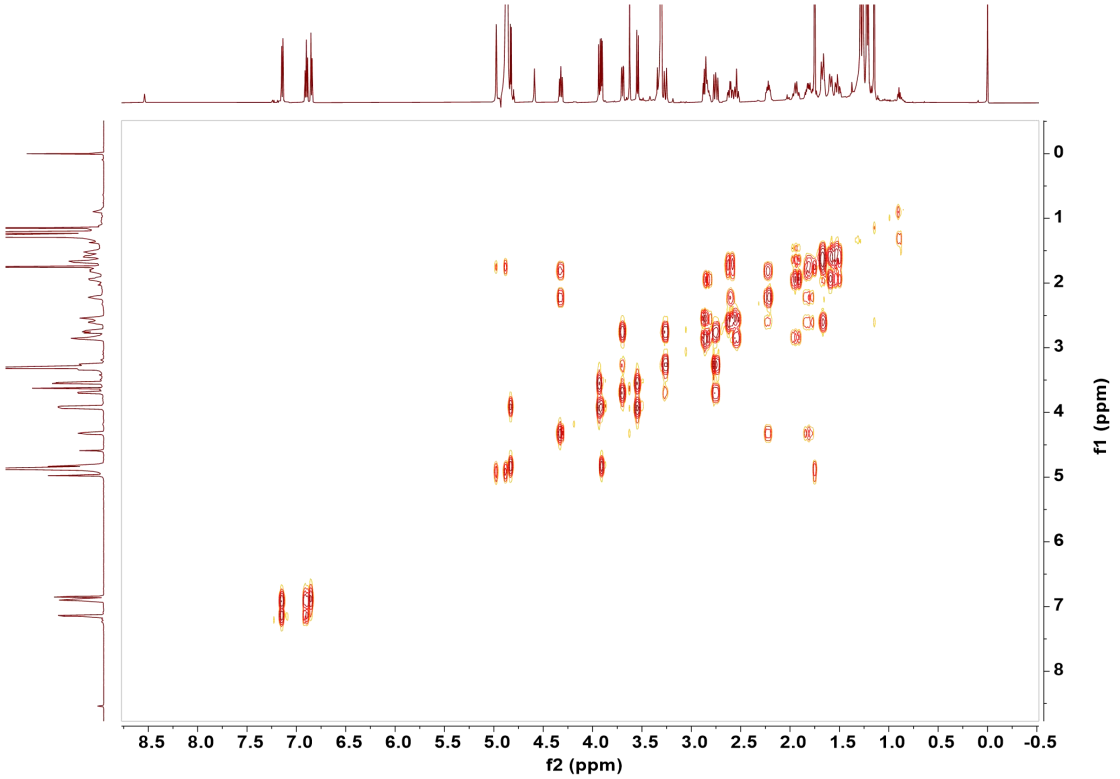

Figure S43. ^1H-^1H spectrum of **5** (600 MHz, CD_3OD).

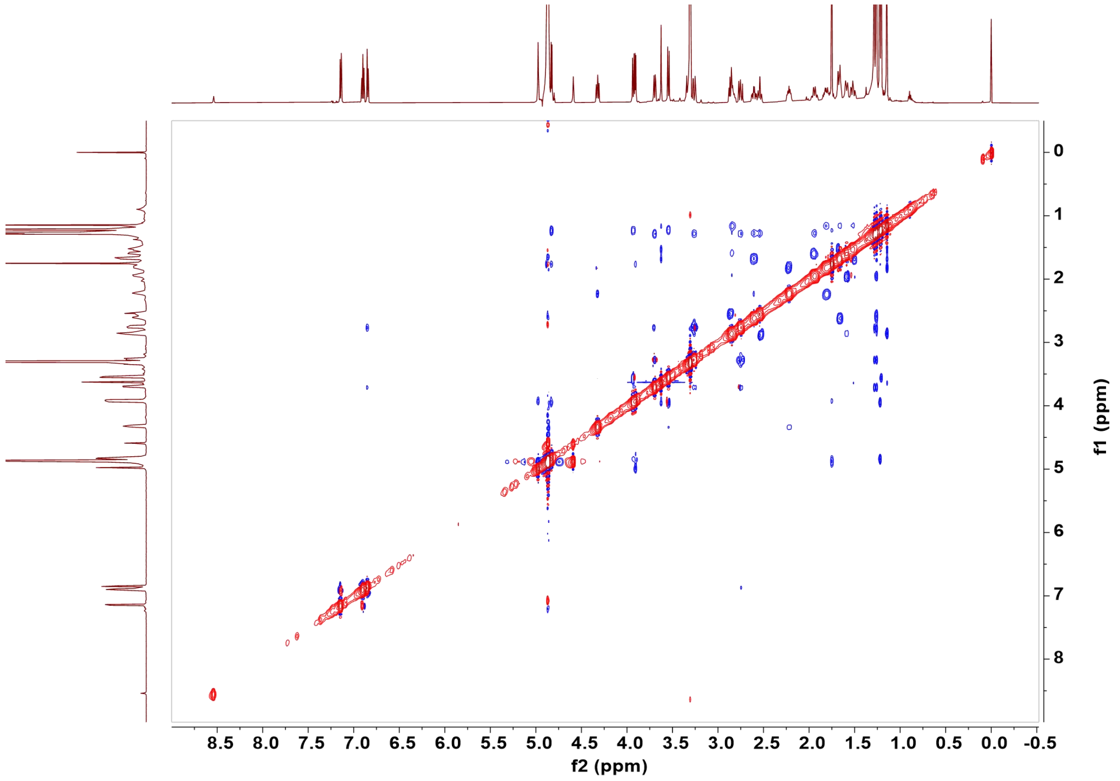

Figure S44. NOESY spectrum of **5** (600 MHz, CD_3OD).

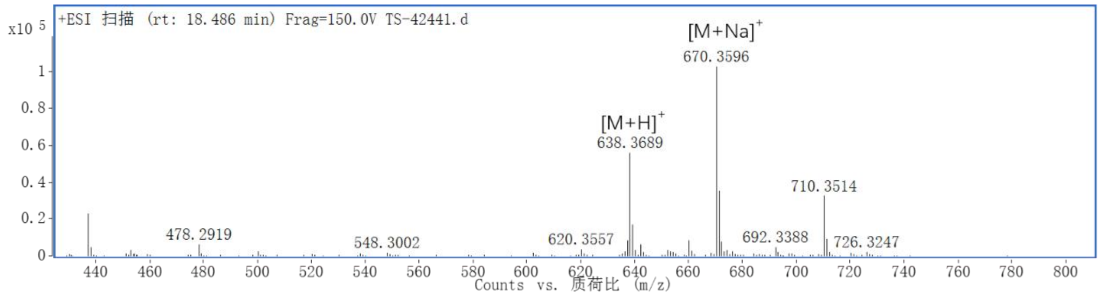

Figure S45. HRESIMS spectrum of **5**.

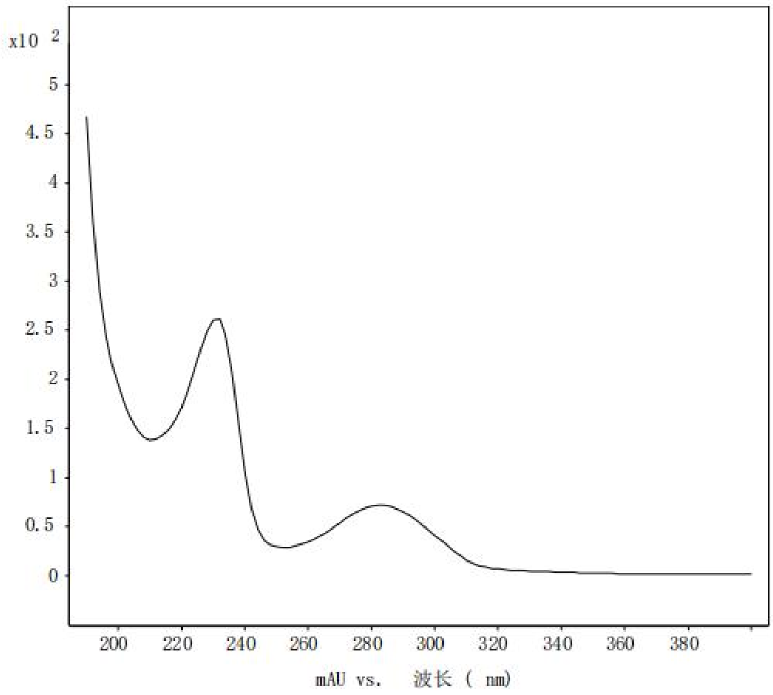

Figure S46. UV spectrum of **5**.

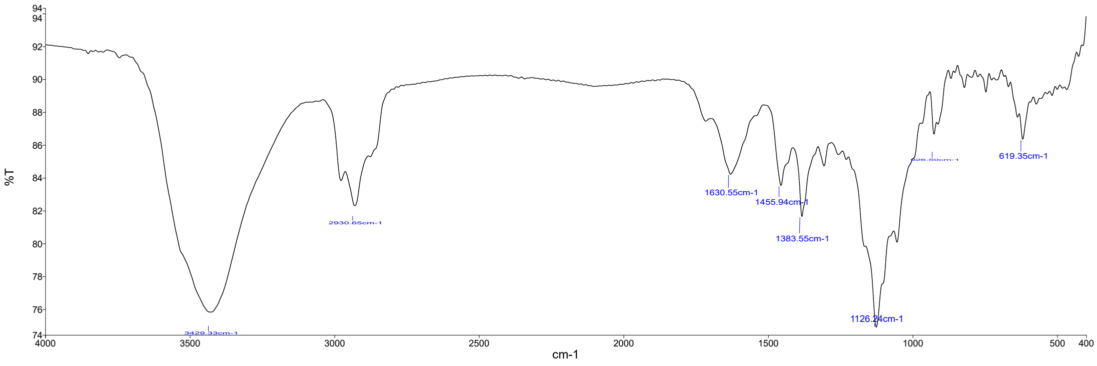

Figure S47. IR spectrum of **5**.

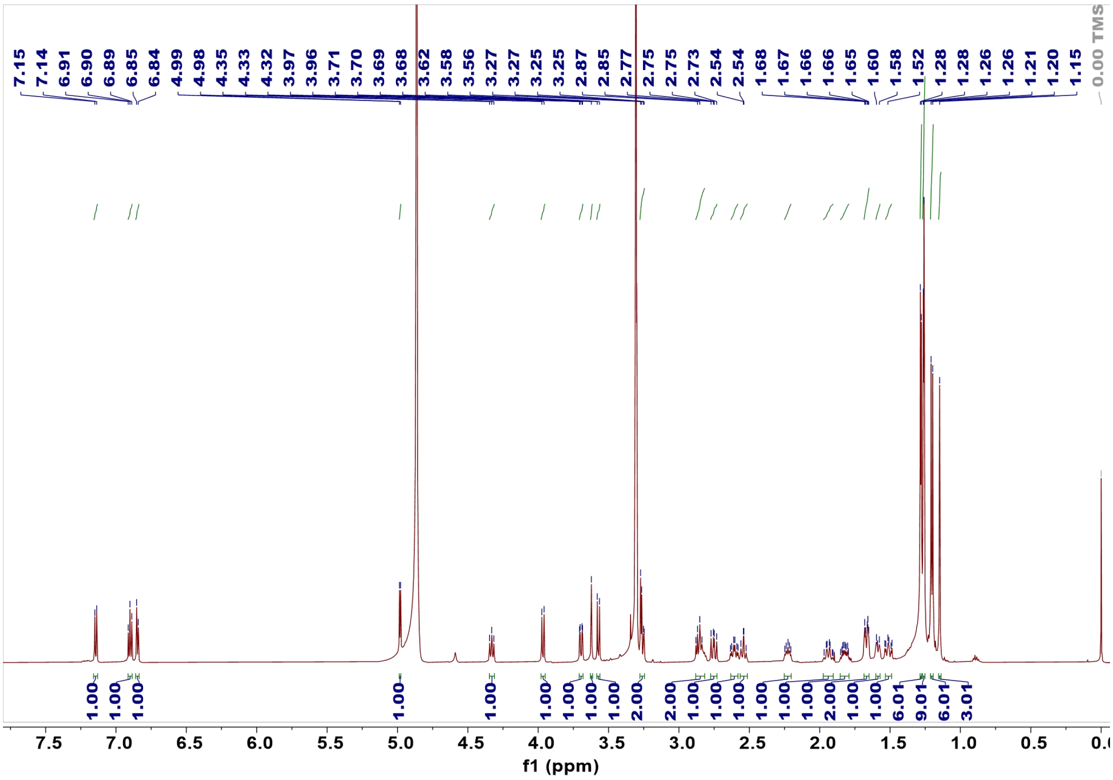

Figure S48. ^{1}H spectrum of **6** (600 MHz, CD_3OD).

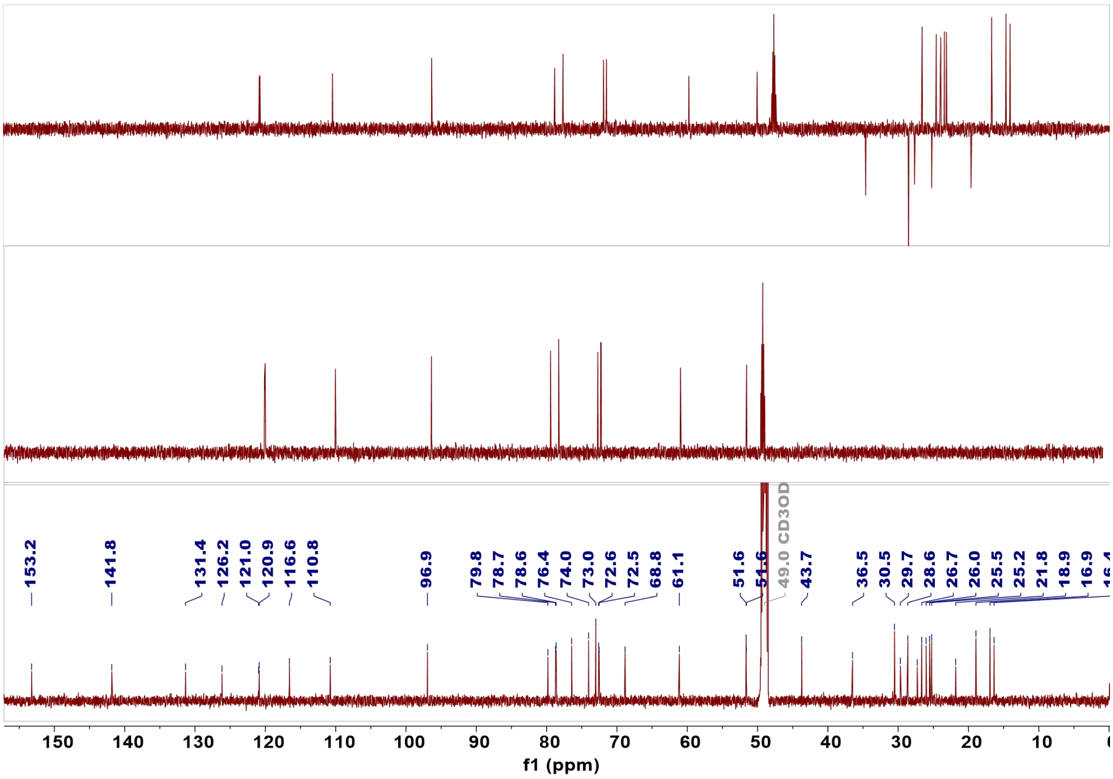

Figure S49. ^{13}C spectrum of **6** (150 MHz, CD_3OD).

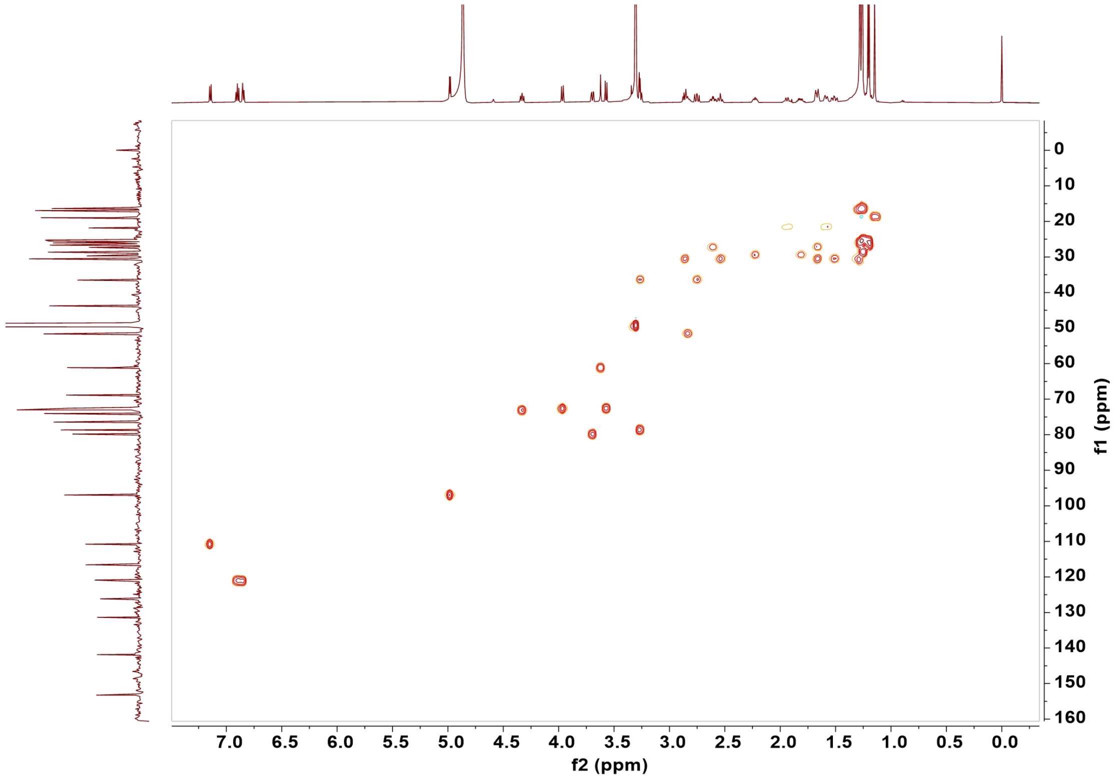

Figure S50. HSQC spectrum of **6** (600 MHz, CD_3OD).

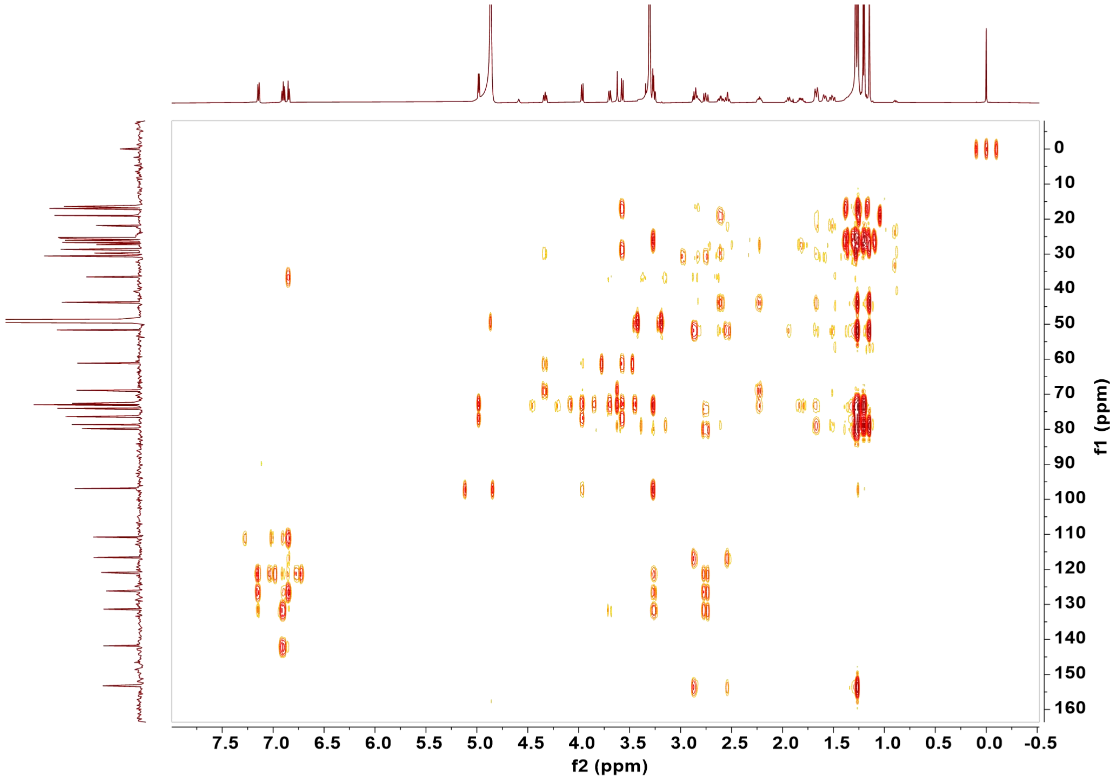

Figure S51. HMBC spectrum of **6** (600 MHz, CD_3OD).

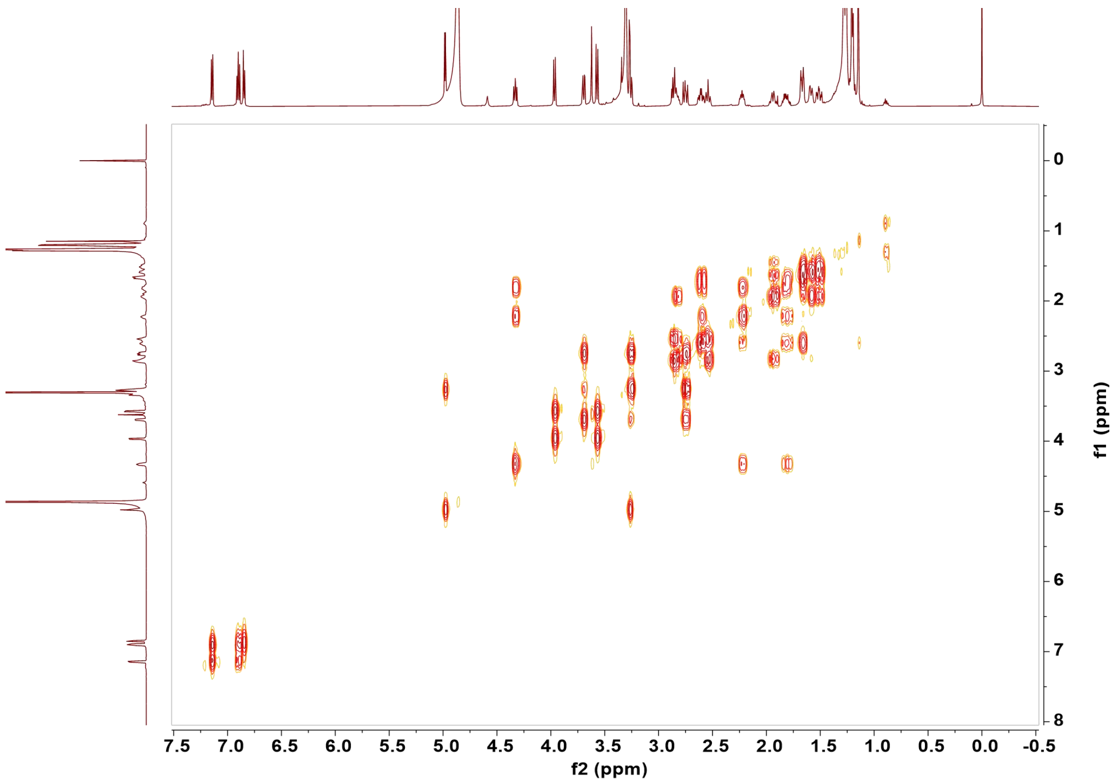

Figure S52. ^{1}H-^{1}H spectrum of **6** (600 MHz, CD_3OD).

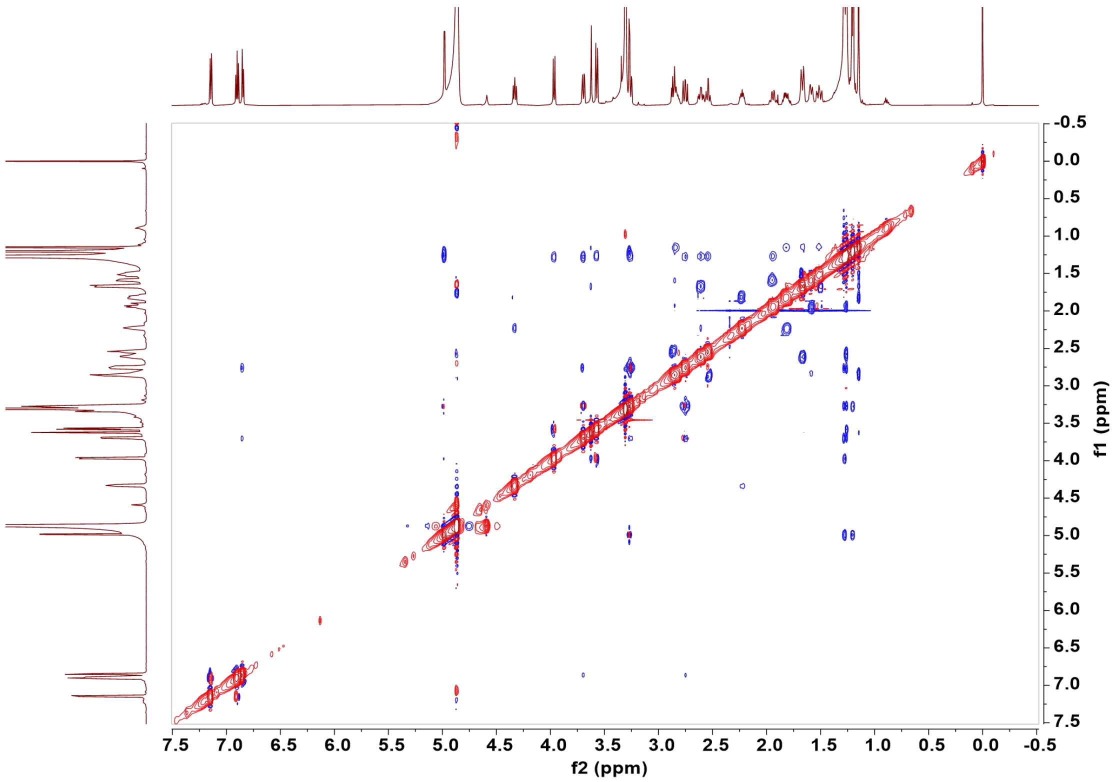

Figure S53. NOESY spectrum of **6** (600 MHz, CD_3OD).

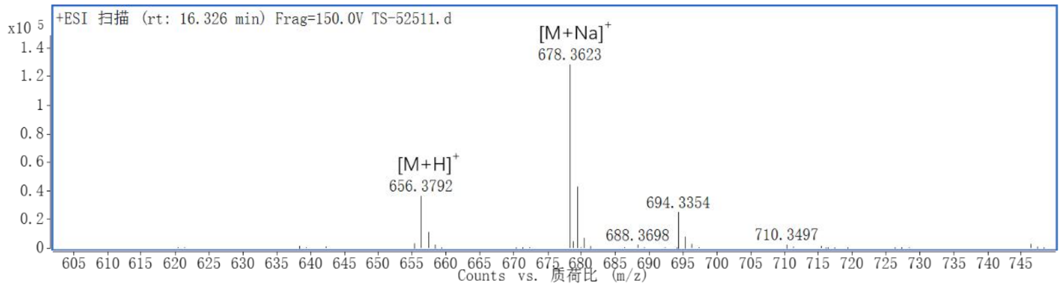

Figure S54. HRESIMS spectrum of **6**.

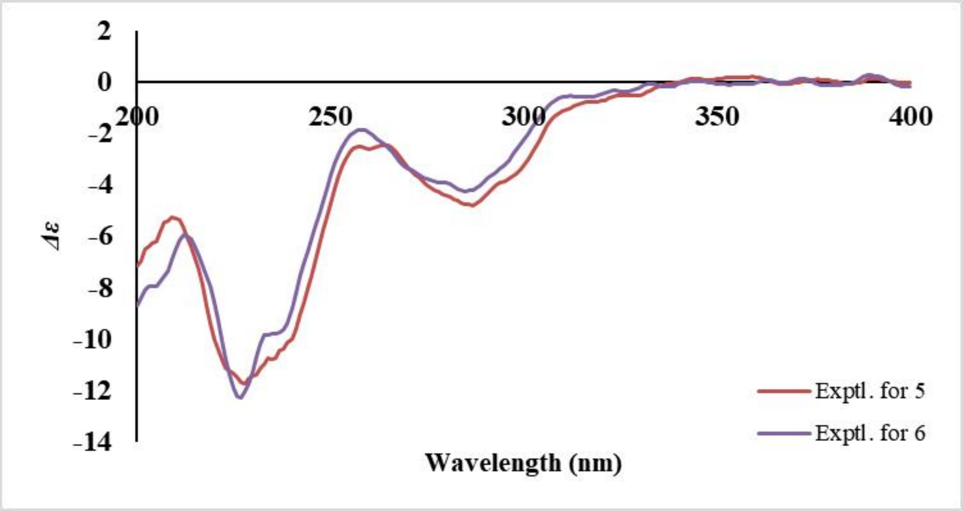

Figure S55. Experimental ECD spectra of compounds **5** and **6**.

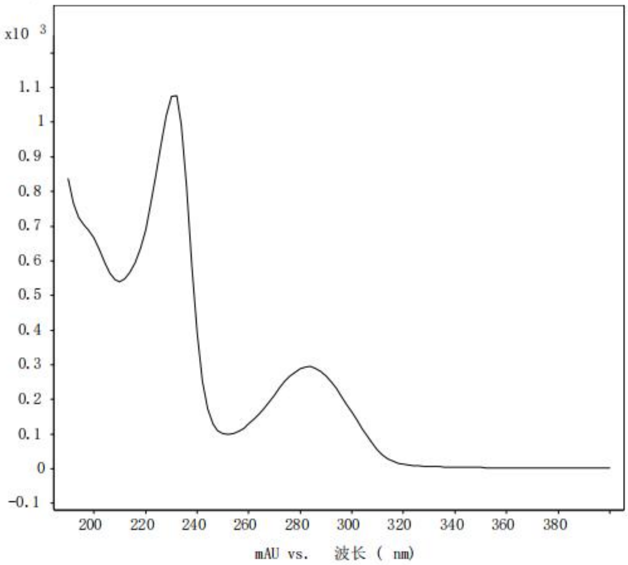

Figure S56. UV spectrum of **6**.

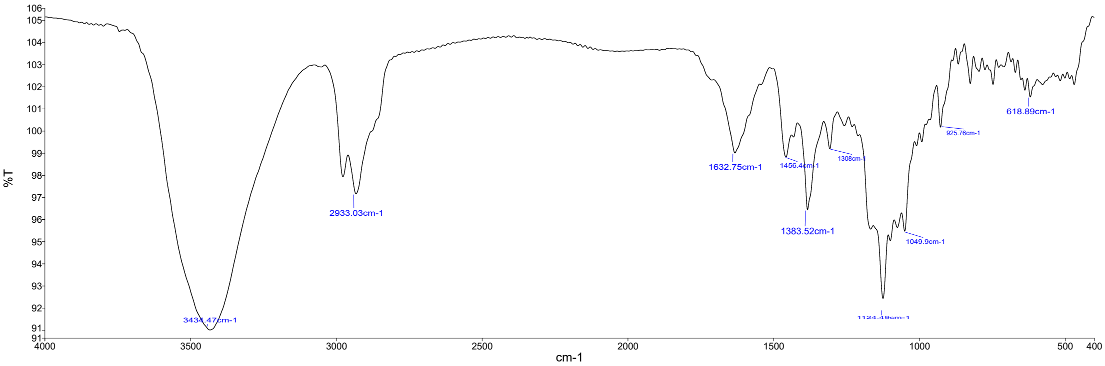

Figure S57. IR spectrum of **6**.

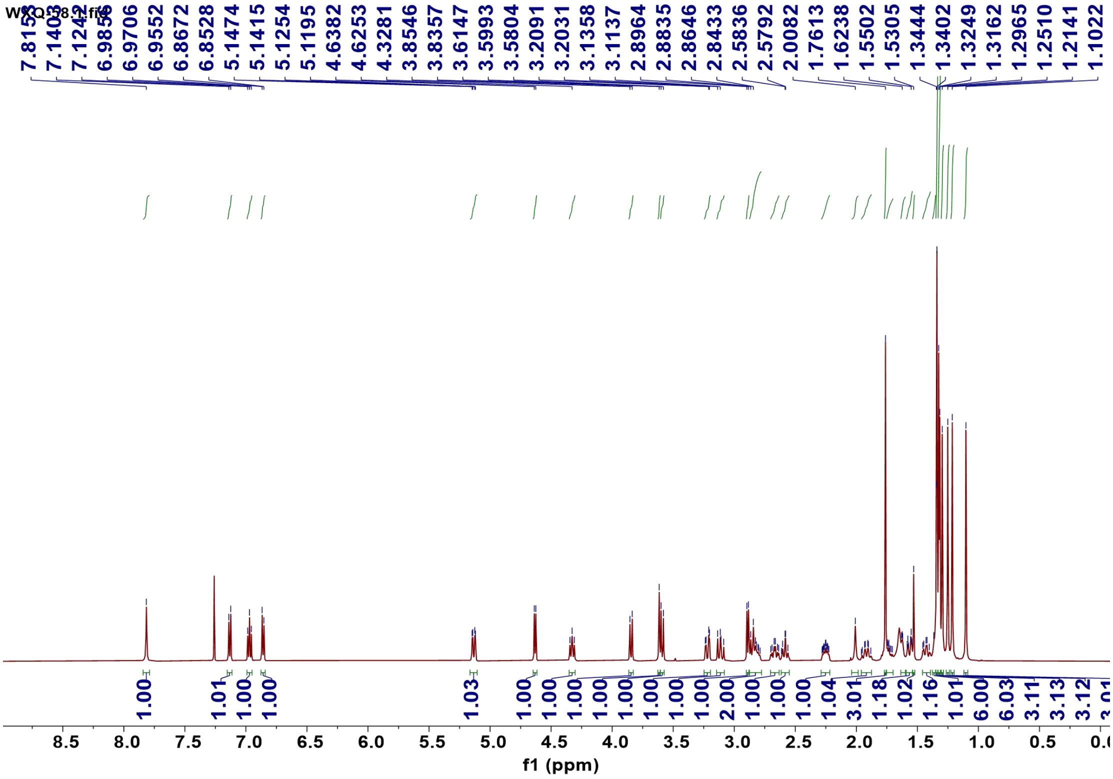

Figure S58. ^{1}H spectrum of **7** (600 MHz, $CDCl_3$).

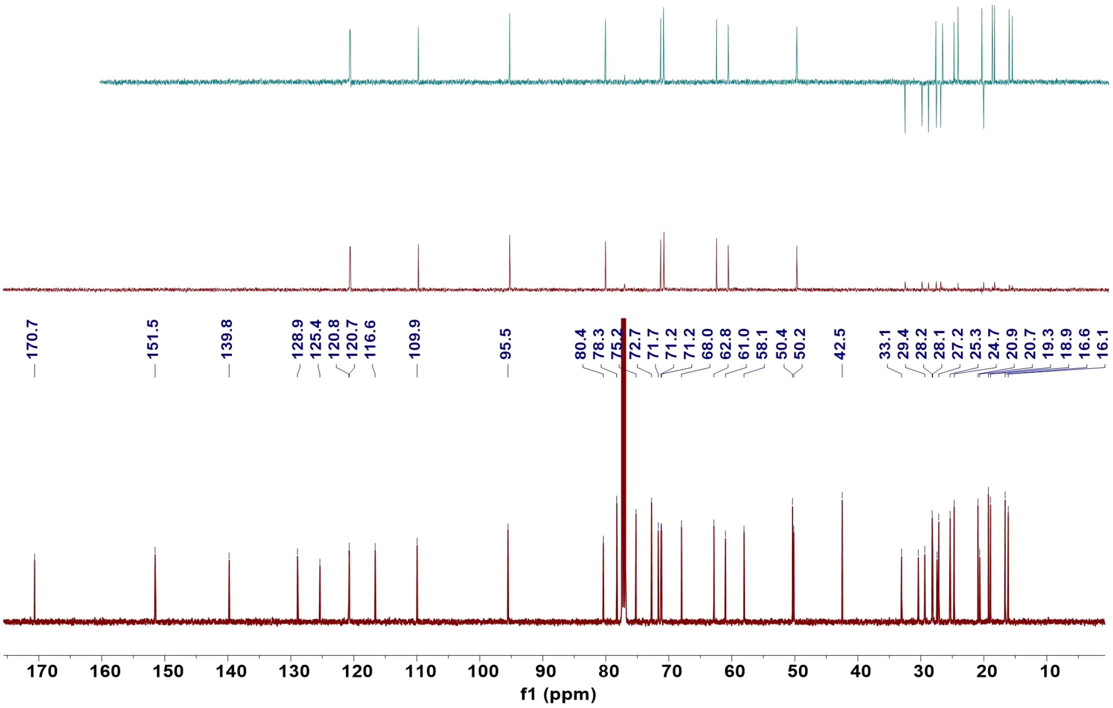

Figure S59. ^{13}C spectrum of **7** (150 MHz, $CDCl_3$).

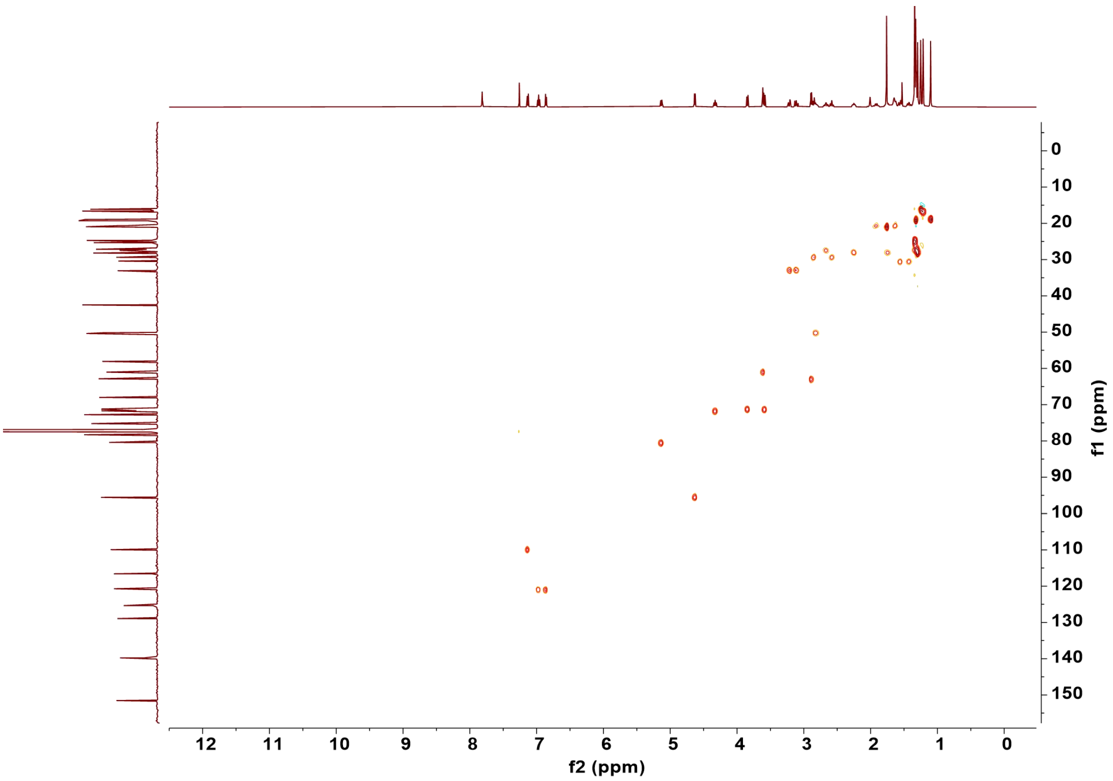

Figure S60. HSQC spectrum of **7** (600 MHz, $CDCl_3$).

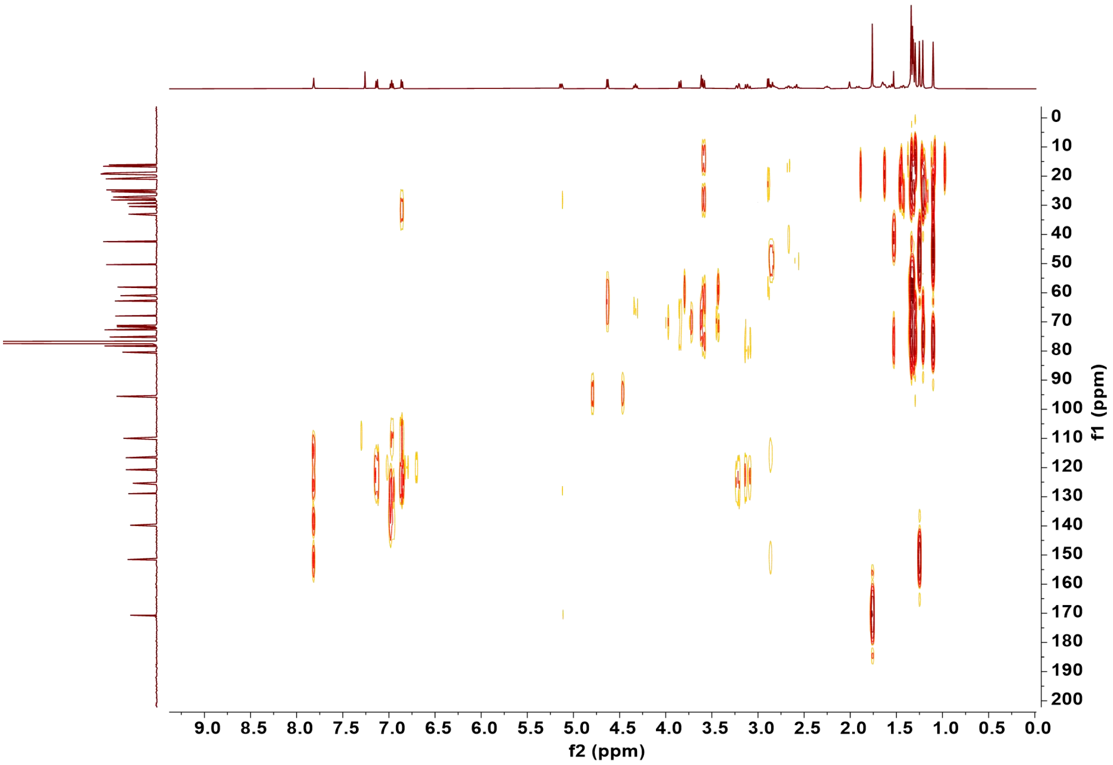

Figure S61. HMBC spectrum of **7** (600 MHz, $CDCl_3$).

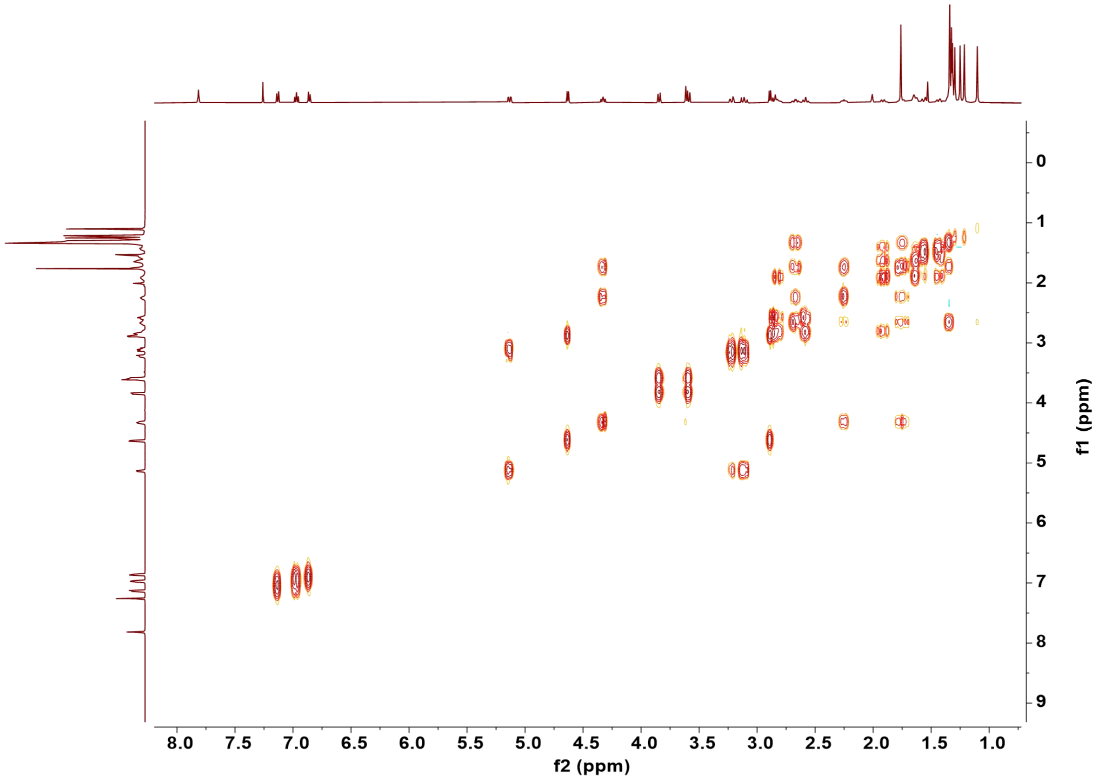

Figure S62. ^{1}H-^{1}H spectrum of **7** (600 MHz, $CDCl_3$).

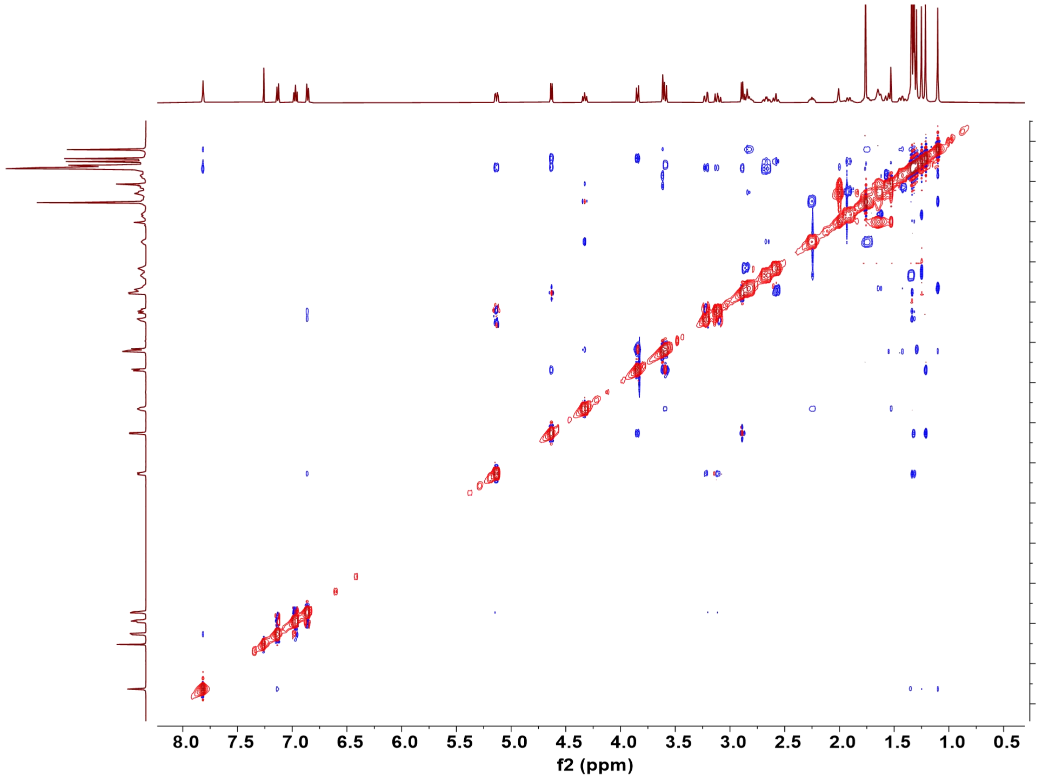

Figure S63. NOESY spectrum of **7** (600 MHz, $CDCl_3$).

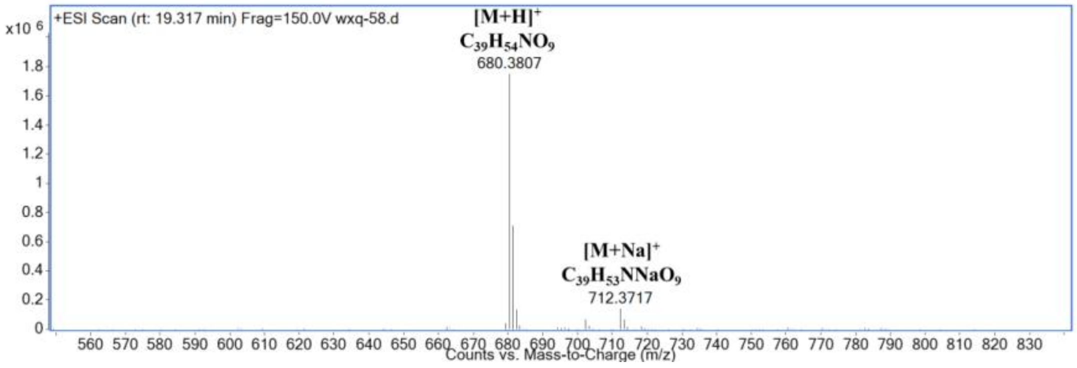

Figure S64. HRESIMS spectrum of **7.**

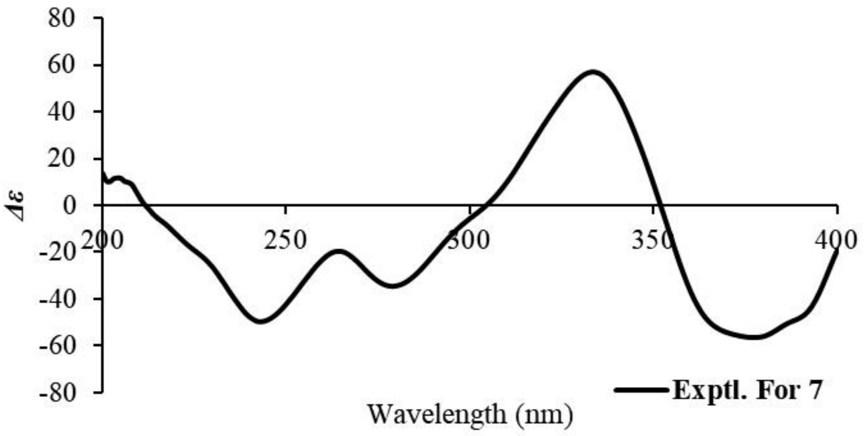

Figure S65. Experimental ECD of **7.**

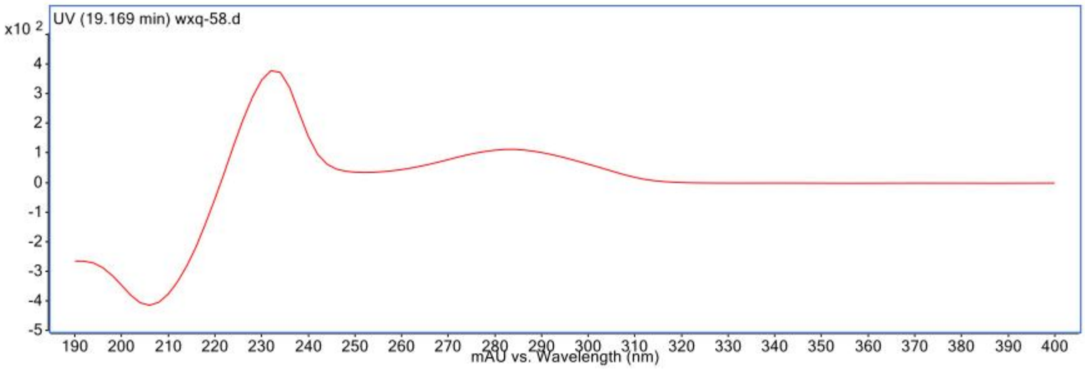

Figure S66. UV spectrum of **7**.

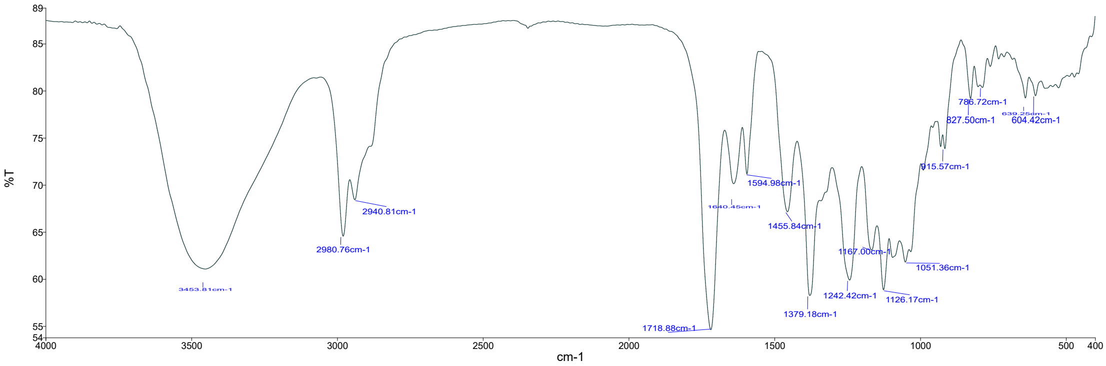

Figure S67. IR spectrum of **7**.

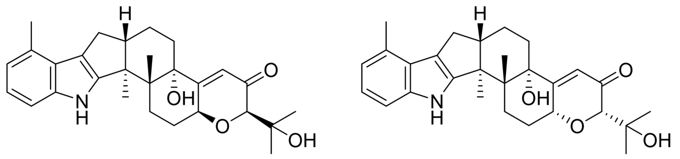

3*S*,4*R*,7*S*,9*R*,13*S*,16*S*-**1a**

MAE :1.06
RMS :1.32
P_{mean}:48.26%
P_{rel} :100.00%

3*S*,4*R*,7*R*,9*S*,13*S*,16*S*-**1b**

MAE :1.61
RMS :2.20
P_{mean}:9.20%
P_{rel} :0.00%

Figure S68. The ^{13}C NMR chemical shifts calculation results of two isomers of **1** (flexible side chain was simplified)

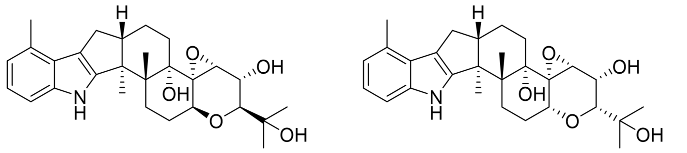

3*S*,4*R*,7*S*,9*S*,10*R*,11*R*,12*S*,13*S*,16*S*-**2a**

MAE :0.79
RMS :1.00
P_{mean}:51.03%
P_{rel} :100.00%

3*S*,4*R*,7*R*,9*R*,10*R*,11*R*,12*S*,13*S*,16*S*-**2b**

MAE :2.31
RMS :3.46
P_{mean}:0.00%
P_{rel} :0.00%

Figure S69. The ^{13}C NMR chemical shifts calculation results of two isomers of **2** (flexible side chain was simplified)

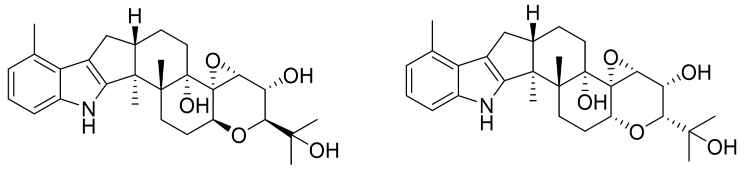

3*S*,4*R*,7*S*,9*S*,10*R*,11*R*,12*S*,13*S*,16*S*-**3a** 3*S*,4*R*,7*R*,9*R*,10*R*,11*R*,12*S*,13*S*,16*S*-**3b**

MAE :0.94	MAE :2.28
RMS :1.23	RMS :3.33
P_{mean}:38.72%	P_{mean}:0.00%
P_{rel} :100.00%	P_{rel} :0.00%

Figure S70. The ^{13}C NMR chemical shifts calculation results of two isomers of **3** (flexible side chain was simplified)

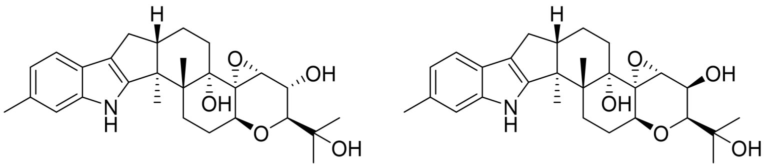

3*S*,4*R*,7*S*,9*S*,10*R*,11*R*,12*S*,13*S*,16*S*-**4a** 3*S*,4*R*,7*S*,9*S*,10*S*,11*R*,12*S*,13*S*,16*S*-**4b**

MAE :0.81	MAE :1.21
RMS :1.01	RMS :1.51
P_{mean}:49.83%	P_{mean}:24.62%
P_{rel} :100.00%	P_{rel} :0.00%

Figure S71. The ^{13}C NMR chemical shifts calculation results of two isomers of **4** (flexible side chain was simplified)

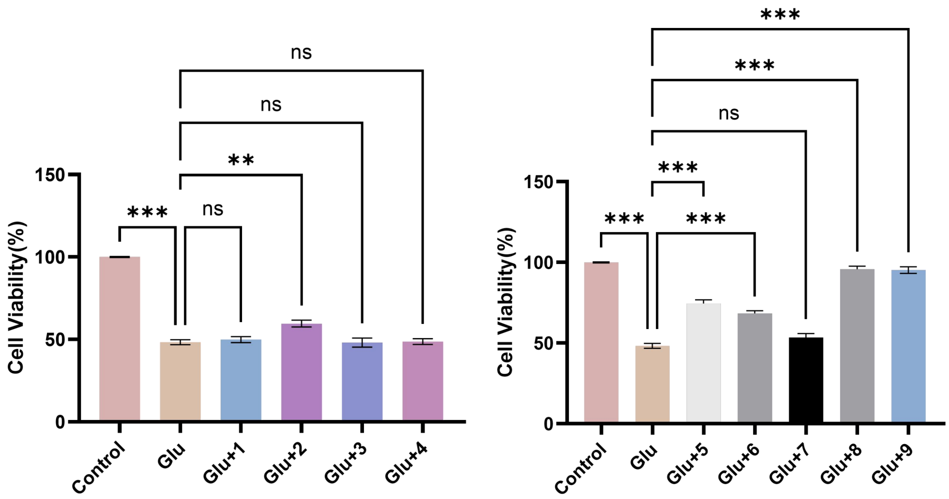

Figure S72. The viability of R28 cells treated with glutamate and 1 μM compounds **1**–**9** for 24 h (n = 3).

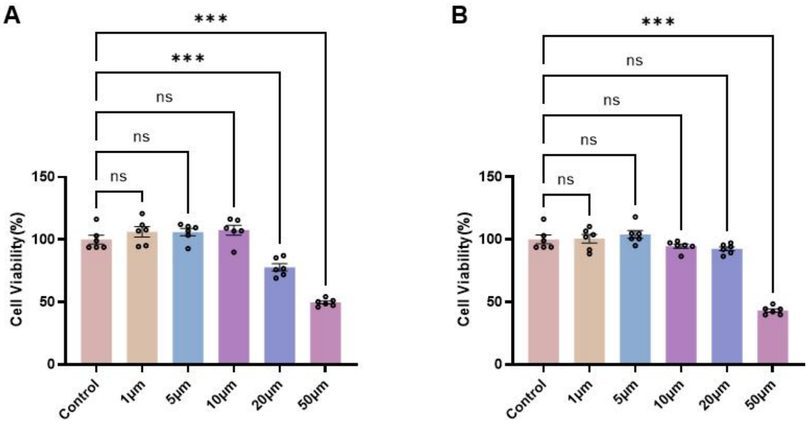

Figure S73. Cytotoxicity evaluation of compounds **8** and **9** on normal R28 cells (n = 3). (A) Cell viability of normal R28 cells treated with various concentrations (1–50 μM) of compound **8** for 24 h. (B) Cell viability of normal R28 cells treated with various concentrations (1–50 μM) of compound **9** for 24 h.

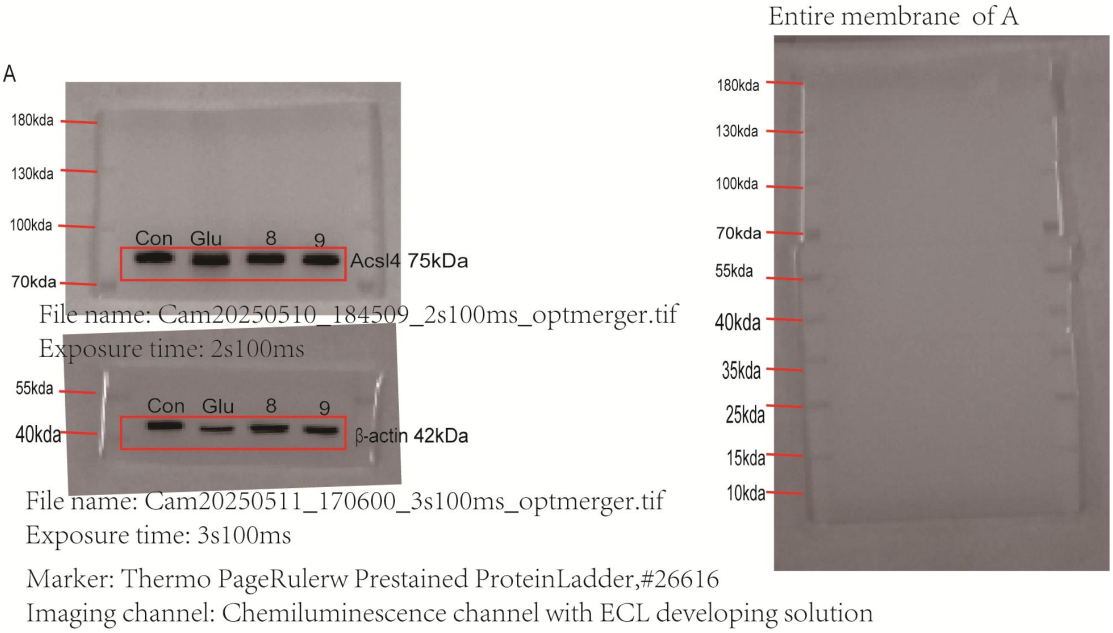

Figure S74. Original Western blot membranes for ACSL4 protein expression.

Entire membrane of B

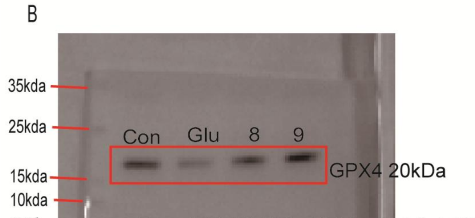

File name: Cam20250506_120049_10s100ms_optmerger.tif
Exposure time: 10s100ms

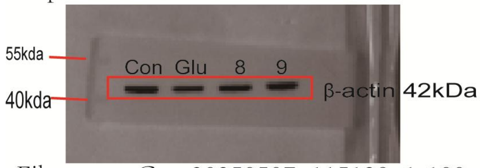

File name: Cam20250507_115129_1s100ms_optmerger.tif
Exposure time: 1s100ms

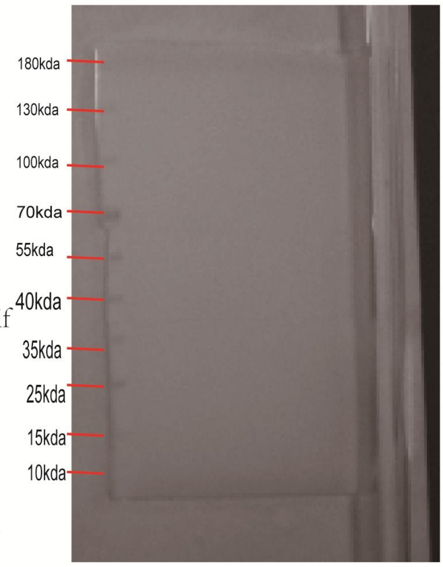

Marker: Thermo PageRulerw Prestained ProteinLadder,#26616
Imaging channel: Chemiluminescence channel with ECL developing solution

Figure S75. Original Western blot membranes for GPX4 protein expression.

Entire membrane of C

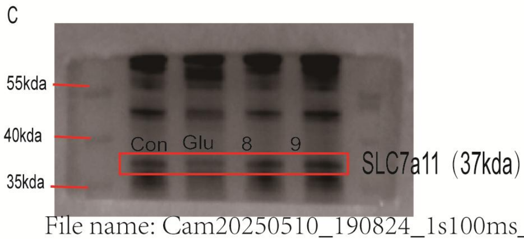

File name: Cam20250510_190824_1s100ms_optmerger.tif
Exposure time: 1s100ms

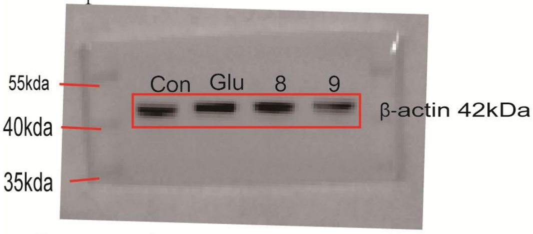

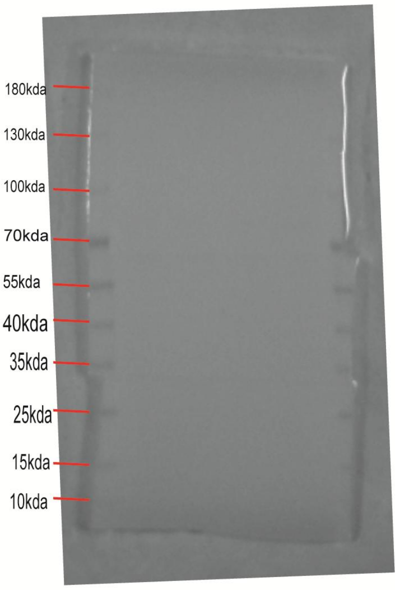

File name: Cam20250511_171008_3s100ms_optmerger.tif
Exposure time: 3s100ms
Marker: Thermo PageRulerw Prestained ProteinLadder,#26616
Imaging channel: Chemiluminescence channel with ECL developing solution

Figure S76. Original Western blot membranes for SLC7a11 protein expression.

Table S1. ^{1}H data for compounds **1–7** (600 MHz, δ in ppm, J in Hz).

No.	**1**[a]	**2**[a]	**3**[a]	**4**[a]	**5**[a]	**6**[a]	**7**[b]
5	1.77–1.80, m<br>2.74–2.78, m	1.64–1.65, m<br>2.61, td (13.5, 5.3)	1.64–1.66, m<br>2.60–2.63, m	1.63–1.67, m<br>2.59–2.63, m	1.65–1.67, m<br>2.61, td (13.6, 5.2)	1.65–1.68, m<br>2.61, td (13.6, 5.2)	1.35, td (13.4, 5.2)<br>2.64–2.70, overlapped
6	1.89–1.92, m<br>2.30–2.32, m	1.83, td (13.5, 3.0)<br>2.24–2.29, m	1.80–1.85, m<br>2.24–2.29, m	1.78–1.84, m<br>2.23–2.29, m	1.79–1.82, m<br>2.20–2.24, m	1.81–1.85, m<br>2.21–2.25, m	1.70–1.76, m<br>2.23–2.28, m
7	4.89–4.91, m	4.18, t (8.9)	4.18, t (8.9)	4.18, t (8.9)	4.32, t (9.0)	4.33, t (8.9)	4.33, t (9.1)
9	3.76, d (10.3)	3.35, d (9.1)	3.35, d (9.1)	3.35, d (9.1)	3.54, d (9.5)	3.57, d (9.5)	3.58, br d (9.5)
10		3.94, d (9.1)	3.93, d (9.1)	3.92, d (9.1)	3.94, d (9.5)	3.96, d (9.5)	3.84, d (9.5)
11	5.86, br s		3.49, br s	3.48, br s	3.63, br s	3.62, br s	3.61, br s
14	1.72–1.75, m<br>2.00–2.03, m	1.45–1.51, m<br>1.66–1.68, m	1.50–1.52, m<br>1.68–1.69, m	1.49, td (13.0, 4.1)<br>1.63–1.67, m	1.51, td (13.0, 4.2)<br>1.65–1.67, m	1.51, td (13.0, 4.2) 1.65–1.68, m	1.42, td (12.9, 4.0) 1.56, overlapped
15	1.72–1.75, m<br>2.05–2.09, m	1.57–1.59, m<br>1.90–1.98, m	1.57–1.60, m<br>1.91–1.98, m	1.56–1.58, m<br>1.93, dd (12.9, 3.0)	1.58–1.60, m<br>1.91–1.97, m	1.58–1.60, m<br>1.91–1.97, m	1.62–1.63, m<br>1.88–1.96, m
16	2.86–2.90, m	2.80–2.84, m	2.85–2.88, m	2.78–2.82, m	2.82–2.88, overlapped	2.84–2.88, overlapped	2.79–2.83, m
17	2.56–2.60, m<br>2.86–2.90, m	2.51–2.57, m<br>2.80–2.84, m	2.56–2.58, m<br>2.85–2.88, m	2.33, dd, (12.9, 10.9)<br>2.59–2.63, m	2.55, m<br>2.82–2.88, overlapped	2.52–2.56, m<br>2.84–2.88, overlapped	2.58, td (14.6, 4.4) 2.84–2.86, m
20				6.79, d (8.0)			
21	6.86, d (7.9)	6.77, d (8.0)	6.74, d (7.9)	7.17, d (8.0)	6.85, d (7.9)	6.85, d (7.1)	6.85, d (7.2)
22	6.90, t (7.9)	6.88, t (8.0)	6.86, t (7.9)		6.90, t (7.9)	6.90, d (7.1)	6.85, d (7.2)
23	7.15, d (7.9)	7.15, d (8.0)	7.11, d (7.9)	7.15, s	7.15, d (7.9)	7.14, d (7.1)	6.97, dd (7.5,7.5)
25	1.33, s	1.27, s	1.28, s	1.25, s	1.26, s	1.26, s	1.25, s
26	1.02, s	1.12, s	1.13, s	1.11, s	1.15, s	1.15, s	1.10, s
28	1.27, s	1.22, d (2.6)	1.21, d (2.8)	1.22, d (3.6)	1.21, s	1.28, d (3.9)	1.30, s
29	1.27, s	1.22, d (2.6)	1.21, d (2.8)	1.22, d (3.6)	1.22, s	1.26, s	1.21, s
30	2.74–2.78, m<br>3.26–3.28, m	3.06, d (6.9)	3.04, dd (13.6, 11.4)<br>3.23, dd (13.6, 2.8)	2.75, dd (14.0, 10.8)<br>3.09, dd (14.0, 2.6)	2.75, dd (13.8, 10.6)<br>3.26, dd (13.8, 2.6)	2.74, dd (13.7, 10.5)<br>3.26, dd (13.7, 2.6)	3.11, dd (13.8, 11.0)<br>3.22, dd (13.8, 3.0)
31	3.71, d (10.3)	4.39, t (6.9)	5.11, dd (11.4, 2.8)	5.04, dd (10.8, 2.6)	3.70, dd (10.6, 2.6)	3.69, dd (10.5, 2.6)	5.14, dd (11.0, 3.0)
33	1.29, s	4.74–4.75, m<br>4.81, m	1.28, s	1.25, s		1.28, d (3.9)	1.34, s
34	1.29, s	1.78, s	1.28, s	1.26, s		1.26, s	1.32, s

36	1.66, s	1.86, s		4.98, d (4.6)	4.63, d (6.4)
38			4.88, overlapped<br>4.98, br s	3.26, dd (13.7, 2.6)	2.88, d (6.4)
39			1.75, s	1.21, d (6.9)	1.34, overlapped
41				1.21, d (6.9)	1.32, overlapped

[a]Measured in CD_3OD. [b]Measured in $CDCl_3$.

Table S2. ^{13}C NMR data for compounds **1–7** (150 MHz, δ in ppm).

No.	**1**[a]	**2**[a]	**3**[a]	**4**[a]	**5**[a]	**6**[a]	**7**[b]
	δ_C, type	δ_C, type	δ_C, type	δ_C, type	δ_C, type	δ_C, type	δ_C, type
2	153.3, C	153.4, C	153.4, C	153.6, C	153.3, C	153.2, C	151.5, C
3	51.8, C	51.6, C	51.6, C	51.9, C	51.6, C	51.6, C	50.4, C
4	44.2, C	43.6, C	43.6, C	43.5, C	43.7, C	43.7, C	42.5, C
5	27.9, CH_2	27.2, CH_2	27.2, CH_2	27.2, CH_2	27.3, CH_2	27.3, CH_2	27.4, CH_2
6	29.9, CH_2	29.3, CH_2	29.3, CH_2	29.2, CH_2	29.7, CH_2	29.7, CH_2	28.1, CH_2
7	74.6, CH	72.8, CH	72.8, CH	72.7, CH	73.0, CH	73.0, CH	71.7, CH
9	84.6, CH	77.6, CH	77.6, CH	77.6, CH	72.7, CH	72.5, CH	71.2, CH
10	200.1, C	68.6, CH	68.6, CH	68.4, CH	72.6, CH	72.5, CH	71.2, CH
11	120.2, C	64.9, CH	64.9, CH	64.9, CH	61.2, CH	61.2, CH	61.0, CH
12	171.8, C	70.5, C	70.5, C	70.7, C	68.8, C	68.8, C	68.0, C
13	77.7, C	78.5, C	78.5, C	78.5, C	78.7, C	78.7, C	78.3, C
14	34.5, CH_2	30.5, CH_2	30.5, CH_2	30.5, CH_2	30.5, CH_2	30.5, CH_2	30.5, CH_2
15	22.2, CH_2	21.8, CH_2	21.9, CH_2	21.8, CH_2	21.8, CH_2	21.8, CH_2	20.7, CH_2
16	51.2, CH	51.6, CH	51.6, CH	51.4, CH	51.6, CH	51.6, CH	50.2, CH
17	30.6, CH_2	30.4, CH_2	30.6, CH_2	28.0, CH_2	30.5, CH_2	30.5, CH_2	29.4, CH_2
18	116.6, C	116.3, C	116.4, C	116.9, C	116.6, C	116.6, C	116.6, C
19	126.2, C	126.0, C	126.5, C	124.9, C	126.2, C	126.2, C	125.4, C
20	131.5, C	130.3, C	129.6, C	118.4, CH	131.4, C	131.4, C	128.9, C
21	121.0, CH	121.0, CH	120.9, CH	121.6, CH	110.8, CH	120.9, CH	120.7, CH
22	121.1, CH	120.8, CH	120.7, CH	130.9, C	120.9, CH	121.0, CH	120.8, CH
23	110.8, CH	111.0, CH	110.9, CH	113.2, CH	121.0, CH	110.8, CH	109.0, CH
24	141.9, C	141.8, C	141.7, C	141.9, C	141.8, C	141.8, C	139.8, C
25	16.6, CH_3	16.4, CH_3	16.6, CH_3	16.4, CH_3	16.4, CH_3	16.4, CH_3	16.1, CH_3
26	19.6, CH_3	18.1, CH_3	18.9, CH_3	18.9, CH_3	18.9, CH_3	18.9, CH_3	18.9, CH_3
27	73.4, C	73.6, C	73.6, C	73.6, C	75.9, C	76.4, C	75.2, C
28	25.3, CH_3	25.0, CH_3	25.0, CH_3	25.2, CH_3	28.6, CH_3	26.0, CH_3	28.2, CH_3
29	25.3, CH_3	27.4, CH_3	27.4, CH_3	27.2, CH_3	16.9, CH_3	16.9, CH_3	16.6, CH_3
30	36.5, CH_2	41.2, CH_2	34.1, CH_2	37.1, CH_2	36.5, CH_2	36.5, CH_2	33.1, CH_2
31	79.9, CH	77.7, CH	81.9, CH	82.6, CH	79.8, CH	79.8, CH	80.4, CH
32	74.1, C	148.9, C	72.9, C	72.9, C	74.0, C, C	74.0, C	72.7, C
33	26.1, CH_3	111.7	25.8, CH_3	25.9, CH_3	25.2	25.5	27.2
34	26.3, CH_3	18.9, CH_3	26.2, CH_3	26.1, CH_3	26.0	28.6	25.3
35			172.3, C	172.9, C	97.7, CH	96.9, CH	95.5, CH
36			20.9, CH_3	21.1, CH_3	77.4, CH	78.6, CH	62.8, CH
37					145.3, C	73.0, CH	58.1, CH
38					114.1, CH_2	25.2, CH_3	24.7, CH_3
39					18.9, CH_3	26.7, CH_3	19.3, CH_3
40							170.7, C
41							20.9, CH_3

[a]Measured in CD_3OD. [b]Measured in $CDCl_3$.

Table S3. The experimental ^{13}C NMR chemical shifts of compound **1** and the GFN2NMR-predicted data of two possible isomers *31S*-**1a** and *31R*-**1a**.

No.	Exptl.	*31S*-**1a**	Deviation	*31R*-**1a**	Deviation
2	153.3	150.94	2.36	149.78	3.52
3	51.8	49.91	1.89	48.65	3.15
4	44.2	45.25	1.05	43.67	0.53

5	27.9	27.78	0.12	28.30	0.40
6	29.9	29.60	0.30	31.26	1.36
7	74.6	71.89	2.71	71.82	2.78
9	84.6	85.34	0.74	85.69	1.09
10	200.1	200.95	0.85	200.94	0.84
11	120.2	121.71	1.51	122.18	1.98
12	171.8	172.02	0.22	168.81	2.99
13	77.7	78.49	0.79	78.95	1.25
14	34.5	32.63	1.87	33.76	0.74
15	22.2	21.90	0.30	23.18	0.98
16	51.2	51.45	0.25	49.96	1.24
17	30.6	29.35	1.25	29.78	0.82
18	116.6	119.23	2.63	118.58	1.98
19	126.2	126.83	0.63	128.02	1.82
20	131.5	129.81	1.69	131.57	0.07
21	121	122.13	1.13	123.41	2.41
22	121.1	122.35	1.25	123.48	2.38
23	110.8	111.62	0.82	111.67	0.87
24	141.9	139.82	2.08	140.11	1.79
25	16.6	19.11	2.51	18.41	1.81
26	19.6	18.70	0.90	18.22	1.38
27	73.4	73.09	0.31	74.28	0.88
28	25.3	26.35	1.05	26.48	1.18
29	25.3	27.39	2.09	26.51	1.21
30	36.5	36.79	0.29	36.15	0.35
31	79.9	77.36	2.54	77.48	2.42
32	74.1	74.02	0.08	74.30	0.20
33	26.1	26.80	0.70	25.53	0.57
34	26.3	26.17	0.13	25.88	0.42
		**MAE**	1.16	**MAE**	1.42
		**RMS**	1.42	**RMS**	1.68
		**P_{mean}**	59.25%	**P_{mean}**	51.45%
		**DP4**	98.92%	**DP4**	1.08%

Table S4. The experimental ^{13}C NMR chemical shifts of compound **2** and the GFN2NMR-predicted data of two possible isomers *31S*-**2a** and *31R*-**2a**.

No.	Exptl.	*31S*-**2a**	Deviation	*31R*-**2a**	Deviation
2	153.4	150.28	3.12	150.41	2.99
3	51.6	47.71	3.89	47.78	3.82
4	43.6	44.07	0.47	44.07	0.47
5	27.2	26.48	0.72	26.43	0.77
6	29.3	26.05	3.25	26.13	3.17
7	72.8	74.53	1.73	74.56	1.76
9	77.6	77.92	0.32	78.11	0.51
10	68.6	70.08	1.48	70.05	1.45
11	64.9	61.01	3.89	61.01	3.89
12	70.5	69.30	1.20	69.46	1.04
13	78.5	79.33	0.83	79.34	0.84
14	30.5	33.79	3.29	33.81	3.31
15	21.8	24.24	2.44	24.29	2.49
16	51.6	48.26	3.34	48.27	3.33
17	30.4	28.63	1.77	28.84	1.56
18	116.3	117.59	1.29	117.78	1.48
19	126	129.13	3.13	128.94	2.94
20	130.3	130.71	0.41	131.04	0.74
21	121	123.57	2.57	123.67	2.67

22	120.8	123.25	2.45	123.37	2.57
23	111	112.04	1.04	112.16	1.16
24	141.8	140.85	0.95	141.19	0.61
25	16.4	18.99	2.59	18.95	2.55
26	18.1	20.28	2.18	20.32	2.22
27	73.6	73.40	0.20	73.43	0.17
28	25	26.46	1.46	26.52	1.52
29	27.4	26.49	0.91	26.43	0.97
30	41.2	40.53	0.67	39.88	1.32
31	77.7	76.30	1.40	75.68	2.02
32	148.9	146.23	2.67	144.57	4.33
33	111.7	111.59	0.11	112.64	0.94
34	18.9	19.30	0.40	19.26	0.36
		**MAE**	1.75	**MAE**	1.87
		**RMS**	2.09	**RMS**	2.20
		$\boldsymbol{P}_{\mathbf{mean}}$	41.35%	$\boldsymbol{P}_{\mathbf{mean}}$	38.49%
		**DP4**	90.83%	**DP4**	9.17%

Table S5. The experimental ^{13}C NMR chemical shifts of compound **3** and the GFN2NMR-predicted data of two possible isomers *31S*-**3a** and *31R*-**3a**.

No.	Exptl.	*31S*-**3a**	Deviation	*31R*-**3a**	Deviation
2	153.4	151.01	2.39	151.20	2.20
3	51.6	47.97	3.63	48.39	3.21
4	43.6	44.35	0.75	44.81	1.21
5	27.2	27.01	0.19	28.17	0.97
6	29.3	27.17	2.13	27.87	1.43
7	72.8	73.94	1.14	74.20	1.40
9	77.6	77.47	0.13	77.19	0.41
10	68.6	69.23	0.63	70.47	1.87
11	64.9	60.43	4.47	61.60	3.30
12	70.5	69.07	1.43	69.91	0.59
13	78.5	79.05	0.55	79.45	0.95
14	30.5	33.52	3.02	31.42	0.92
15	21.9	24.22	2.32	22.49	0.59
16	51.6	49.27	2.33	50.06	1.54
17	30.6	29.84	0.76	29.10	1.50
18	116.4	118.94	2.54	118.52	2.12
19	126.5	127.09	0.59	128.51	2.01
20	129.6	129.22	0.38	129.21	0.39
21	120.9	123.01	2.11	123.82	2.92
22	120.7	123.18	2.48	123.66	2.96
23	110.9	111.69	0.79	112.48	1.58
24	141.7	140.57	1.13	140.26	1.44
25	16.6	19.33	2.73	19.42	2.82
26	18.9	19.82	0.92	20.13	1.23
27	73.6	73.25	0.35	74.30	0.70
28	25	25.98	0.98	26.81	1.81
29	27.4	26.12	1.28	24.65	2.75
30	34.1	33.64	0.46	36.59	2.49
31	81.9	81.14	0.76	76.61	5.29
32	72.9	71.62	1.28	71.37	1.53
33	25.8	27.37	1.57	22.97	2.83
34	26.2	26.07	0.13	28.32	2.12
		**MAE**	1.37	**MAE**	1.80
		**RMS**	1.75	**RMS**	2.09

		***P*_{mean}**	51.44%	***P*_{mean}**	40.72%
		**DP4**	99.96%	**DP4**	0.04%

Table S6. The experimental ^{13}C NMR chemical shifts of compound **4** and the GFN2NMR-predicted data of two possible isomers *31S*-**4a** and *31R*-**4a**.

No.	Exptl.	*31S*-**4a**	Deviation	*31R*-**4a**	Deviation
2	153.6	151.02	2.58	151.43	2.17
3	51.9	48.43	3.47	48.43	3.47
4	43.5	44.25	0.75	43.93	0.43
5	27.2	26.66	0.54	27.38	0.18
6	29.2	26.27	2.93	25.96	3.24
7	72.7	74.22	1.52	73.76	1.06
9	77.6	77.74	0.14	77.77	0.17
10	68.4	70.22	1.82	70.16	1.76
11	64.9	61.11	3.79	61.27	3.63
12	70.7	69.36	1.34	69.57	1.13
13	78.5	79.19	0.69	78.48	0.02
14	30.5	33.92	3.42	34.17	3.67
15	21.8	24.58	2.78	24.47	2.67
16	51.4	48.36	3.04	49.06	2.34
17	28	28.25	0.25	28.48	0.48
18	116.9	118.40	1.50	118.22	1.32
19	124.9	127.02	2.12	125.88	0.98
20	118.4	118.71	0.31	118.33	0.07
21	121.6	123.68	2.08	122.83	1.23
22	130.9	134.02	3.12	134.53	3.63
23	113.2	113.80	0.60	114.44	1.24
24	141.9	139.73	2.17	139.67	2.23
25	16.4	18.74	2.34	19.15	2.75
26	18.9	20.36	1.46	19.60	0.70
27	73.6	73.40	0.20	73.13	0.47
28	25.2	26.95	1.75	26.40	1.20
29	27.2	26.51	0.69	26.91	0.29
30	37.1	35.49	1.61	33.93	3.17
31	82.6	80.84	1.76	80.96	1.64
32	72.9	70.91	1.99	73.02	0.12
33	25.9	26.33	0.43	26.32	0.42
34	26.1	26.38	0.28	26.38	0.28
35	172.9	172.41	0.49	172.76	0.14
36	21.1	20.36	0.74	20.82	0.28
		**MAE**	1.61	**MAE**	1.43
		**RMS**	1.93	**RMS**	1.87
		***P*_{mean}**	45.36%	***P*_{mean}**	48.89%
		**DP4**	7.26%	**DP4**	92.74%

Table S7. The experimental ^{13}C NMR chemical shifts of compound **5** and the GFN2NMR-predicted data of four possible isomers 31*S*,36*S*-**5**, 31*S*,36*R*-**5,** 31*R*,36*R*-**5,** and 31*R*,36*S*-**5,** respectively.

No.	Exptl.	31*S*,36 *S*-**5**	Deviation	31*S*,36 *R*-**5**	Deviation	31*R*,36 *R*-**5**	Deviation	31*R*,36 *S*-**5**	Deviation
2	153.3	151.12	2.18	151.01	2.29	150.38	2.92	150.69	2.61
3	51.6	47.90	3.70	47.98	3.62	47.87	3.73	47.88	3.72
4	43.7	44.24	0.54	44.49	0.79	44.19	0.49	44.18	0.48
5	27.3	26.72	0.58	27.06	0.24	26.82	0.48	26.76	0.54
6	29.7	27.51	2.19	27.56	2.14	27.56	2.14	27.56	2.14
7	73	74.67	1.67	74.76	1.76	74.44	1.44	74.44	1.44

9	72.7	69.74	2.96	68.58	4.12	69.72	2.98	69.62	3.08
10	72.6	70.31	2.29	72.26	0.34	69.96	2.64	70.04	2.56
11	61.2	60.11	1.09	60.39	0.81	60.10	1.10	60.01	1.19
12	68.8	69.09	0.29	69.53	0.73	69.00	0.20	68.99	0.19
13	78.7	79.19	0.49	79.38	0.68	79.13	0.43	79.13	0.43
14	30.5	34.18	3.68	34.49	3.99	34.21	3.71	34.19	3.69
15	21.8	24.16	2.36	24.50	2.70	24.21	2.41	24.23	2.43
16	51.6	49.18	2.42	49.36	2.24	49.24	2.36	49.23	2.37
17	30.5	28.87	1.63	29.33	1.17	28.94	1.56	28.82	1.68
18	116.6	119.23	2.63	118.90	2.30	118.83	2.23	118.70	2.10
19	126.2	128.54	2.34	128.68	2.48	129.07	2.87	129.08	2.88
20	131.4	131.28	0.12	130.91	0.49	131.52	0.12	131.09	0.31
21	110.8	122.58	11.78	123.13	12.33	123.77	12.97	123.30	12.50
22	120.9	123.44	2.54	123.45	2.55	123.88	2.98	123.46	2.56
23	121	112.02	8.98	112.02	8.98	111.91	9.09	111.89	9.11
24	141.8	141.01	0.79	140.94	0.86	140.80	1.00	140.89	0.91
25	16.4	19.13	2.73	19.49	3.09	19.26	2.86	19.20	2.80
26	18.9	19.40	0.50	19.67	0.77	19.54	0.64	19.46	0.56
27	75.9	75.07	0.83	75.94	0.04	75.23	0.67	75.25	0.65
28	28.6	25.10	3.50	22.25	6.35	24.84	3.76	24.10	4.50
29	16.9	22.47	5.57	22.42	5.52	22.81	5.91	23.53	6.63
30	36.5	36.26	0.24	37.01	0.51	36.40	0.10	36.74	0.24
31	79.8	77.59	2.21	77.87	1.93	78.17	1.63	78.47	1.33
32	74	74.55	0.55	74.79	0.79	74.44	0.44	74.74	0.74
33	26	25.81	0.19	25.88	0.12	25.40	0.60	26.17	0.17
34	25.2	25.31	0.11	25.53	0.33	25.81	0.61	26.03	0.83
35	97.7	98.40	0.70	97.56	0.14	98.12	0.42	98.07	0.37
36	77.4	74.86	2.54	75.65	1.75	75.88	1.52	75.59	1.81
37	145.3	142.76	2.54	141.30	4.00	142.96	2.34	143.29	2.01
38	114.1	115.05	0.95	115.01	0.91	113.34	0.76	113.82	0.28
39	18.9	20.45	1.55	18.21	0.69	19.54	0.64	18.65	0.25
		**MAE**	2.21	**MAE**	2.38	**MAE**	2.24	**MAE**	2.22
		**RMS**	3.21	**RMS**	3.42	**RMS**	3.36	**RMS**	3.35
		$\boldsymbol{P}_{\mathbf{mean}}$	22.59%	$\boldsymbol{P}_{\mathbf{mean}}$	19.91%	$\boldsymbol{P}_{\mathbf{mean}}$	20.74%	$\boldsymbol{P}_{\mathbf{mean}}$	20.99%
		**DP4**	89.46%	**DP4**	0.84%	**DP4**	3.78%	**DP4**	5.92%

Table S8. The experimental ^{13}C NMR chemical shifts of compound **7** and the GFN2NMR-predicted data of four possible isomers 31*S*,36*S*-**7**, 31*S*,36*R*-**7**, 31*R*,36*R*-**7**, and 31*R*,36*S*-**7**, respectively.

No.	Exptl.	31*S*,36 *S*-**7**	Deviation	31*S*,36 *R*-**7**	Deviation	31*R*,36 *R*-**7**	Deviation	31*R*,36 *S*-**7**	Deviation
2	151.5	149.25	2.25	148.67	2.83	149.45	2.05	149.89	1.61
3	50.4	46.58	3.82	45.84	4.56	46.68	3.72	46.54	3.86
4	42.5	44.49	1.99	43.88	1.38	44.44	1.94	44.40	1.90
5	27.4	27.50	0.10	27.24	0.16	27.10	0.30	26.78	0.62
6	28.1	27.43	0.67	27.27	0.83	27.97	0.13	27.84	0.26
7	71.7	73.52	1.82	73.54	1.84	74.17	2.47	73.86	2.16
9	71.2	67.49	3.71	68.18	3.02	68.05	3.15	69.55	1.65
10	71.2	72.74	1.54	70.18	1.02	70.01	1.19	70.65	0.55
11	61.0	58.61	2.39	58.31	2.69	58.48	2.52	58.80	2.20
12	68.0	68.44	0.44	68.86	0.86	68.46	0.46	68.24	0.24
13	78.3	78.56	0.26	78.33	0.03	79.15	0.85	79.09	0.79
14	30.5	32.90	2.50	32.83	2.43	33.09	2.69	32.39	1.99
15	20.7	23.47	2.77	22.58	1.88	23.80	3.10	23.23	2.53
16	50.2	48.79	1.41	49.01	1.19	48.52	1.68	48.60	1.60
17	29.4	29.09	0.31	29.01	0.39	29.10	0.30	28.67	0.73
18	116.6	118.65	2.05	117.37	0.77	117.70	1.10	117.86	1.26

19	125.4	126.04	0.64	127.73	2.33	127.03	1.63	126.46	1.06
20	128.9	129.15	0.25	128.30	0.60	127.24	1.66	127.81	1.09
21	120.7	121.17	0.47	121.73	1.03	122.82	2.12	123.07	2.37
22	120.8	121.78	0.98	121.29	0.49	121.32	0.52	121.44	0.64
23	109.0	110.98	1.08	110.75	0.85	111.13	1.23	111.04	1.14
24	139.8	139.07	0.73	138.43	1.37	138.32	1.48	138.90	0.90
25	16.1	17.99	1.89	18.14	2.04	18.60	2.50	18.34	2.24
26	18.9	18.84	0.06	18.40	0.50	18.29	0.61	17.86	1.04
27	75.2	75.37	0.17	75.44	0.24	75.14	0.06	76.10	0.90
28	28.2	21.80	6.40	18.77	9.43	22.54	5.66	23.50	4.70
29	16.6	23.39	6.79	27.45	10.85	22.53	5.93	23.02	6.42
30	33.1	34.37	1.27	34.07	0.97	33.68	0.58	34.11	1.01
31	80.4	79.93	0.47	80.79	0.39	78.35	2.05	77.18	3.22
32	72.7	72.19	0.51	72.32	0.38	72.14	0.56	71.99	0.71
33	27.2	27.29	0.09	24.32	2.88	25.81	1.39	27.42	0.22
34	25.3	25.46	0.16	27.32	2.02	25.74	0.44	25.07	0.23
35	95.5	95.67	0.17	98.46	2.96	98.15	2.65	96.76	1.26
36	62.8	61.03	1.77	63.23	0.43	63.06	0.26	62.29	0.51
37	58.1	59.81	1.71	59.28	1.18	59.72	1.62	58.50	0.40
38	24.7	20.57	4.13	20.80	3.90	20.79	3.91	21.15	3.55
39	19.3	20.26	0.96	21.79	2.49	21.36	2.06	20.69	1.39
40	170.7	170.41	0.29	169.79	0.91	170.58	0.12	170.38	0.32
41	20.9	19.71	1.19	20.09	0.81	19.28	1.62	20.30	0.60
		**MAE**	1.54	**MAE**	1.92	**MAE**	1.75	**MAE**	1.54
		**RMS**	2.21	**RMS**	2.92	**RMS**	2.23	**RMS**	2.02
		P_{mean}	42.40%	P_{mean}	28.40%	P_{mean}	39.53%	P_{mean}	45.13%
		**DP4**	8.02%	**DP4**	0.00%	**DP4**	0.52%	**DP4**	91.45%

Table S9. ITS sequence of *Tolypocladium album* DWS131

GGAAGTAAAAGTCGTAACAAGGTCTCCGTTGGTGAACCAGCGGAGGGATCATTACCG
AGTTATCAACTCCCAAACCCCTGTGAACATACCTGAACGTTGCCTCGGCGGGACCGCC
CCGGCGCCCAACTCGCGGCCCGGACCCAGGCGCCCGCCGGAGGACCCAAACTCTTGC
TTTAAACAGTGGCATACTCTCTGAGTCTCACAAACAAAAAATGAATCAAAACTTTCAA
CAACGGATCTCTTGGCTCTGGCATCGATGAAGAACGCAGCGAAATGCGATAAGTAATG
TGAATTGCAGAATTCAGTGAATCATCGAATCTTTGAACGCACATTGCGCCCGCCAGCAT
TCTGGCGGGCATGCCTGTCCGAGCGTCATTTCAACCCTCAGGGCCCCCCTTCGCGGGG
CGGGACCTGGTGTTGGGGGCCGGCCGCCCTGCGCGCGCCGCCCCCGAAATGCAGTGG
CGACCTCGCCGCAGCCTCCCCTGCGTAGTAGCACAACCTCGCACCGGAGCGCGGAGA
CGGTCACGCCGTAAAACGCCCAACTTTCAAGAGTTGACCTCGGATCAGGTAGGAATAC
CCGCTGAACTTAAGCATATCA
